# Supplementary material for: High‐Throughput Heterospheroid‐Based Screening Identifies Drugs That Reprogram Tumor‐Associated Macrophages
Source: J Immunol Res. 2026 Apr 20;2026:2889509. doi: 10.1155/jimr/2889509 (PMC13581048; doi:10.1155/jimr/2889509)
Supplement: Supplementary file 1 [file JIMR-2026-2889509-s001.pdf]

Supplementary Table 1. Key research resources

Reagents / Resource	Source	RRID	Authentication
**Cell Lines**			
HLF	Japanese Collection of Research Bioresources Cell Bank (August 2018)	JCRB0405	STR (July, 2024)
HuH6	Japanese Collection of Research Bioresources Cell Bank (August 2018)	JCRB0401	STR (July, 2024)
HLF expressing AcGFP	Our group	N/A	STR (July, 2024)
HuH6 expressing AcGFP	Our group	N/A	STR (July, 2024)
THP-1	RIKEN Bioresource Center (April 2021)	RCB1189	STR (July, 2024)
THP-1 expressing hKO1	Our group	N/A	STR (July, 2024)
Hepa 1-6	RIKEN Bioresource Center (June 2019)	RCB1638	STR (July, 2024)
**Mouse**			
C57BL/6J (male, 6 week old)	CLEA Japan	N/A	
**Chemicals and reagents**			
96-well Ultra Low Attachment Multiple Well Pate	Corning	3474	
Accumax	Nacalai Tesque	17087-54	
Accutase	Nacalai Tesque	12679-54	
Albumin, Bovine Serum, General Grade, pH7.0	Nacalai Tesque	01860-65	
All-trans retinoic acid	Santa Cruz Biotechnology	sc-200898	
Alprostadil	Selleck	S1508	
AR-7	Cayman Chemical	22946	
Carbamazepine	Santa Cruz Biotechnology	SC-202518	
CD2665	Cayman Chemical	16031	
CellTracker™ CM-DiI Dye	Thermo Fisher Scientific	C7000	
Cell Counting Kit-8	Dojindo	CK04	
Chlorocresol	Tokyo Chemical Industry	C0150	
Chloroxine	Sigma-Aldrich	D64600	
Chlortetracycline HCl	Sigma-Aldrich	C4881-5G	
Dipyridamole	Sigma-Aldrich	D9766	
DMEM	Shimadzu Diagnostics	05919	
EGF, Human, Recombinant, Animal Free	Peprotech	AF-100-15	
Emtricitabine	Tokyo Chemical Industry	E1007	
FDA-approved drug library	Center for Supporting Drug Discovery and Life Science Research, Osaka University	N/A	
FGF-basic(154a.a.), Human, Recombinant, Animal Free	Peprotech	AF-100-18B	
G418	Nacalai Tesque	08973-14	
HX531	Cayman Chemical	20762	
IFN-gamma, Human, Recombinant, Animal Free	Peprotech	AF-300-02	
IL-13, Human, Recombinant, Animal Free	Peprotech	AF-200-13	
IL-4, Human, Recombinant, Animal Free	Peprotech	AF-200-04	
LE135	Cayman Chemical	14415	
Lipopolysaccharides (LPS), from Escherichia coli O111:B4	Sigma-Aldrich	L2630	

Lubiprostone	Selleck	S1675
M-CSF, Human, Recombinant, Animal Free	Peprotech	AF-300-25
Misoprostol	Selleck	S4415
Monocytes Spin Medium	pluriSelect	60-00095-10
NEBNext Poly(A) mRNA Magnetic Isolation Module	New England Biolabs	E7490
NEBNext Ultra II Directional RNA Library Prep Kit for Illumina	New England Biolabs	E7760
PA452	MedChemExpress	HY-108522
pAcGFP-C1	TaKaRa Bio	632470
Phorbol 12-myristate 13-acetate (PMA)	Nacalai Tesque	27547-14
PrimeSurface shale 35	Sumitomo Bakelite	MS-90350
Relacorilant	Selleck	E1091
ReverTra Ace	TOYOBO	TRT-101
RPMI1640	Shimadzu Diagnostics	05918
Sepasol-RNA I Super G	Nacalai Tesque	09379-55
THUNDERBIRD SYBR qPCR Mix	TOYOBO	QPS-201
Trometamol	Sigma-Aldrich	T1503-1KG
UVI3003	MedChemExpress	HY-107500

**Antibodies**	**Source**	
Neutralization		
Mouse IgG1κ (clone: P3.6.2.8.1)	Thermo Fisher Scientific	14-4714-85
CD24 (clone: SN3)	Thermo Fisher Scientific	MA511828
Clear Back (Human Fc receptor blocking reagent)	MBL	MTG-001
Mouse treatment		
Rat IgG2aκ (clone: 2A3)	Selleck	A2123
Pd-1 (clone: RMP1-14)	Selleck	A2122
Flowcytometry—human		
CD11b (clone: M1/70)—APC	BioLegend	101212
CD14 (clone: 63D3)—Alexa Fluor 647	BioLegend	367128
CD80 (clone: 2D10.4)—APC	Thermo Fisher Scientific	17-0809-42
CD86 (clone: IT2.2)—Alexa Fluor 647	Thermo Fisher Scientific	51-0869-42
CXCR2 (clone: 5E8/CXCR2)—Alexa Fluor 647	BioLegend	320714
HLA-DR (clone: L243)—APC	BioLegend	321116
TREM2 (clone: 237920R)—Alexa Fluor 647	R&D Systems	FAB17291RR
Flowcytometry—mouse		
Cd45 (clone: 30-F11)—FITC	BioLegend	103108
Cd11b (clone: M1/70)—PerCP	BioLegend	101230
F4/80 (clone: BM8)—PE	BioLegend	123110
I-A/I-E (clone: M5/114.15.2)—APC	BioLegend	107614
Cd206 (clone: C068C2)—APC	BioLegend	141708
Cd3ε (clone: 145-2C11)—PerCP	BioLegend	100326
Cd4 (clone: GK1.5)—PE	BioLegend	100408
Cd8a (clone: 53-6.7)—PE	BioLegend	100708

Pd-1 (clone: RMP1-30)—Alexa Fluor 647	BioLegend	109118

**Primers**	**Sequence**	**Reference sequence**
36B4-sense	TGTTCGACAATGGCAGCATC	NM_001002.4
36B4-antisense	TGATGCAACAGTTGGGTAGC	
CCL2-sense	TAGCAGCCACCTTCATTCCC	NM_002982.4
CCL2-antisense	ACACTTGCTGCTGGTGATTC	
CD80-sense	TCACCATCCAAGTGTCCATACC	NM_005191.4
CD80-antisense	AGCGTTGCCACTTCTTTCAC	
CD86-sense	ACCAACACAATGGAGAGGGAAG	NM_175862
CD86-antisense	AAAACACGCTGGGCTTCATC	
CXCL10-sense	GAATCCAGAATCGAAGGCCATC	NM_001565.4
CXCL10-antisense	GCACTGCATCGATTTTGCTC	
CXCR2-sense	ACCTTGAGGCACAGTGAAGAC	NM_001557.4
CXCR2-antisense	TGGGCTTTTCACCTGTAGGAC	
E2F1-sense	ACCTTCGTAGCATTGCAGAC	NM_005225.3
E2F1-antisense	AAAACATCGATCGGGCCTTG	
FOXM1-sense	ATTCGCCATCAACAGCACTG	NM_202002.3
FOXM1-antisense	AACATGTCGTGCAGGGAAAG	
HLA-DRA-sense	TCATGACAAAGCGCTCCAAC	NM_019111.5
HLA-DRA-antisense	TGGTGGGGTGAACTTGTCTATG	
HLA-DRB1-sense	ACCTACTGCAGACACAACTACG	NM_002124.3
HLA-DRB1-antisense	TGCCTGGATAGAAACCACTCAC	
IL10-sense	ACATCAAGGCGCATGTGAAC	NM_000572.3
IL10-antisense	ACGGCCTTGCTCTTGTTTTC	
IL1RN-sense	TCCTGTGTCAAGTCTGGTGATG	NM_173841.3
IL1RN-antisense	GCTTGTCCTGCTTTCTGTTCTC	
KIF20A-sense	TTGCTGCCCTTCGTCAAAAC	NM_005733.3
KIF20A-antisense	GCCTGTGAAGAAACCTTGGAAC	
PDL2-sense	TGGCAGAAACTTCAGCTGTG	NM_025239.4
PDL2-antisense	ATGTGAAGCAGCCAAGTTGG	
TREM2-sense	CGGCTGCTCATCTTACTCTTTG	NM_018965.4
TREM2-antisense	AGTGCTTCATGGAGTCATAGGG	
VEGFA-sense	GGCAAAAACGAAAGCGCAAG	NM_003376.6
VEGFA-antisense	ACGCGAGTCTGTGTTTTTGC	

Supplementary Table 2. Summary of the first screening

Compounds	MFI of hKO1[1),2)] n = 1	n = 2	mean	toxicity[3)]
Afatinib (BIBW2992)	1.32	1.19	1.26	toxic
Bortezomib (PS-341)	0.00	0.59	0.30	toxic
Everolimus (RAD001)	0.78	0.69	0.73	
Dasatinib	1.53	1.02	1.27	toxic
Erlotinib HCl (OSI-744)	1.44	1.26	1.35	
Gefitinib (ZD1839)	1.47	1.20	1.33	
Imatinib Mesylate (STI571)	0.86	1.07	0.96	
Lapatinib (GW-572016) Ditosylate	1.00	0.96	0.98	
Lenalidomide (CC-5013)	1.01	0.99	1.00	
Nilotinib (AMN-107)	0.99	1.00	0.99	
Rapamycin (Sirolimus)	0.94	0.79	0.86	
Sorafenib Tosylate	0.44	0.90	0.67	
Sunitinib Malate	0.61	0.89	0.75	
Temsirolimus (CCI-779, NSC 683864)	0.69	1.07	0.88	
Vandetanib (ZD6474)	1.09	1.05	1.07	
Vorinostat (SAHA, MK0683)	1.33	1.21	1.27	toxic
Masitinib (AB1010)	1.20	0.93	1.07	
Crizotinib (PF-02341066)	0.96	1.01	0.98	
Vismodegib (GDC-0449)	0.95	0.96	0.96	
Cabozantinib (XL184, BMS-907351)	0.82	0.94	0.88	
Abiraterone (CB-7598)	0.81	0.81	0.81	
Pemetrexed	1.10	1.11	1.10	
Malotilate	0.98	0.91	0.94	
Ivacaftor (VX-770)	1.17	0.65	0.91	
Docetaxel	1.29	1.18	1.24	
Gemcitabine HCl (Gemzar)	1.26	1.24	1.25	
Paclitaxel	1.35	1.08	1.22	
Capecitabine	0.97	1.03	1.00	
Cisplatin	0.99	1.11	1.05	
Valproic acid sodium salt (Sodium valproate)	0.97	1.10	1.03	
Ritonavir	0.99	0.97	0.98	
Axitinib	0.78	0.87	0.83	
Aprepitant	1.00	1.07	1.03	
Regorafenib (BAY 73-4506)	0.70	0.53	0.61	toxic
Fulvestrant	0.98	1.00	0.99	
Thalidomide	1.02	0.80	0.91	
Exemestane	0.96	1.05	1.01	
Finasteride	1.11	0.98	1.04	
Irinotecan	1.22	1.10	1.16	
Cladribine	1.04	1.11	1.08	
Dutasteride	0.95	1.28	1.12	
Melatonin	1.01	0.97	0.99	
Bisoprolol fumarate	0.98	1.11	1.04	
Doxorubicin (Adriamycin)	1.76	0.86	1.31	toxic
Fluorouracil (5-Fluoracil, 5-FU)	1.10	1.22	1.16	
Methotrexate	1.34	1.17	1.26	
Imiquimod	1.04	1.04	1.04	
Bendamustine HCl	1.02	1.10	1.06	
Nelarabine	1.04	1.16	1.10	
Bleomycin Sulfate	1.04	1.13	1.08	
Clafen (Cyclophosphamide)	0.97	0.95	0.96	
Clofarabine	1.57	1.17	1.37	toxic
Dacarbazine	1.01	0.90	0.95	
Dexrazoxane HCl (ICRF-187, ADR-529)	1.17	0.94	1.05	
Epirubicin HCl	1.67	0.87	1.27	toxic
Decitabine	1.05	0.86	0.96	
Etoposide	1.10	1.12	1.11	
Raloxifene HCl	0.89	1.13	1.01	

Idarubicin HCl	1.38	0.94	1.16	
Fludarabine Phosphate	2.08	1.00	1.54	toxic
2-Methoxyestradiol (2-MeOE2)	1.10	1.01	1.05	
Pazopanib HCl (GW786034 HCl)	1.06	0.92	0.99	
Leucovorin Calcium	1.06	1.02	1.04	
Temozolomide	1.01	0.95	0.98	
Vincristine	0.58	0.59	0.59	toxic
Agomelatine	1.07	1.28	1.17	
Leflunomide	0.86	0.96	0.91	
Vinblastine	0.81	0.70	0.75	toxic
Carboplatin	0.94	1.00	0.97	
Topotecan HCl	1.56	0.85	1.21	
Nepafenac	1.07	1.09	1.08	
Rufinamide	1.09	0.97	1.03	
Posaconazole	0.96	0.97	0.96	
Prasugrel	1.12	0.99	1.06	
Ramelteon	1.07	1.11	1.09	
Cinacalcet HCl	1.00	1.13	1.07	
Celecoxib	1.02	1.07	1.05	
Vemurafenib (PLX4032, RG7204)	1.14	1.18	1.16	
Acarbose	1.12	1.04	1.08	
Adapalene	0.84	0.77	0.81	
Amisulpride	1.11	1.04	1.08	
Aniracetam	1.20	0.86	1.03	
Artemisinin	1.12	0.78	0.95	
Asenapine	1.09	1.07	1.08	
Benazepril HCl	1.24	1.05	1.15	
Biperiden HCl	1.15	0.66	0.90	
Budesonide	1.33	0.91	1.12	
Bumetanide	1.26	0.99	1.13	
Camptothecin	1.70	1.11	1.40	toxic
Carmofur	1.65	1.18	1.42	
Cilnidipine	1.09	0.94	1.02	
Cilostazol	1.15	1.05	1.10	
Floxuridine	1.33	1.21	1.27	
FT-207 (NSC 148958)	1.12	0.95	1.03	
Ifosfamide	1.13	1.13	1.13	
Megestrol Acetate	1.20	1.03	1.11	
Mercaptopurine (6-MP)	1.18	0.91	1.04	
Pamidronate Disodium	1.14	0.97	1.05	
Streptozotocin (STZ)	1.10	1.11	1.11	
Zoledronic Acid	1.17	1.05	1.11	
Doxazosin Mesylate	1.02	1.07	1.05	
Edaravone	1.15	0.93	1.04	
Ellagic acid	1.11	0.95	1.03	
Etodolac	1.15	1.04	1.10	
Etomidate	1.16	1.03	1.10	
Felbamate	1.19	0.95	1.07	
Fluconazole	1.02	0.99	1.01	
Flumazenil	1.21	1.02	1.11	
Fluoxetine HCl	1.15	0.95	1.05	
Fluvoxamine maleate	1.12	0.98	1.05	
Gatifloxacin	0.99	0.89	0.94	
Genistein	1.09	1.17	1.13	
Glimepiride	1.17	1.11	1.14	
Granisetron HCl	1.15	0.86	1.01	
Ivermectin	1.10	0.87	0.98	
Ketoconazole	1.13	0.99	1.06	
Lansoprazole	1.09	1.02	1.05	
Levetiracetam	1.10	1.10	1.10	
Lidocaine	1.15	1.19	1.17	
Loratadine	1.09	1.18	1.13	

Acitretin	0.69	0.55	0.62
Biapenem	1.11	1.03	1.07
Cefoselis Sulfate	1.06	0.89	0.97
Daptomycin	1.14	0.96	1.05
Doripenem Hydrate	1.14	1.09	1.12
Dorzolamide HCL	1.10	1.09	1.10
Gestodene	1.14	1.06	1.10
Drospirenone	1.22	0.96	1.09
Altretamine	1.17	1.12	1.14
Isotretinoin	0.84	0.73	0.79
Meropenem	0.86	0.96	0.91
Mianserin HCl	1.13	0.90	1.01
Minoxidil	1.19	0.82	1.00
Mizoribine	1.14	0.94	1.04
Mosapride Citrate	1.14	0.95	1.04
Nafamostat Mesylate	1.29	0.97	1.13
Omeprazole	1.15	0.91	1.03
Ondansetron HCl	1.13	1.11	1.12
Oxcarbazepine	1.14	0.88	1.01
Pizotifen Malate	1.23	1.11	1.17
Rocuronium Bromide	1.03	1.01	1.02
Stavudine (d4T)	1.16	0.97	1.06
Teicoplanin	1.15	0.93	1.04
Tenofovir Disoproxil Fumarate	1.05	1.04	1.04
Tenofovir	1.11	0.98	1.05
Tigecycline	1.14	1.07	1.11
Entecavir Hydrate	1.22	1.02	1.12
Vecuronium Bromide	1.18	1.05	1.12
Bimatoprost	1.14	1.03	1.08
Linezolid	1.15	1.07	1.11
Clopidogrel	1.15	1.10	1.13
Prazosin HCl	1.14	0.92	1.03
Ranolazine 2HCl	1.23	1.06	1.14
Repaglinide	1.14	1.10	1.12
Risedronate sodium	1.20	1.11	1.16
Rolipram	1.20	1.03	1.12
Sildenafil Citrate	1.18	1.01	1.09
Sumatriptan Succinate	1.15	1.00	1.07
Tianeptine sodium	1.15	0.94	1.05
Tizanidine HCl	1.21	1.08	1.14
Tranilast	1.07	1.07	1.07
Varenicline Tartrate	1.04	1.06	1.05
Venlafaxine	0.95	1.14	1.05
Voriconazole	0.89	0.95	0.92
Zileuton	1.08	1.04	1.06
Ziprasidone HCl	1.01	1.02	1.01
Zonisamide	1.03	1.05	1.04
Atazanavir Sulfate	1.03	1.15	1.09
Ofloxacin (Floxin)	0.99	1.00	1.00
Marbofloxacin	1.05	1.12	1.08
Calcitriol	0.78	0.71	0.74
Doxercalciferol	0.79	0.98	0.89
Alfacalcidol	1.06	1.04	1.05
Calcifediol	0.65	0.88	0.76
Iloperidone	0.90	0.89	0.89
Naratriptan	0.92	1.07	1.00
Ponatinib (AP24534)	0.79	0.84	0.81
Fludarabine (Fludara)	1.49	0.99	1.24
Pralatrexate	1.29	1.16	1.23
Cefaclor (Ceclor)	1.07	1.07	1.07
Mycophenolate Mofetil	0.91	1.01	0.96
Cephalexin	0.95	0.97	0.96

Dyphylline	0.89	1.05	0.97
Aztreonam	1.00	1.13	1.06
Perindopril Erbumine (Aceon)	1.06	0.98	1.02
Irbesartan	0.92	0.89	0.90
Alprostadil	0.69	0.83	0.76
Norfloxacin (Norxacin)	0.99	1.07	1.03
Tadalafil	0.96	1.02	0.99
Cyclosporine	1.00	1.05	1.02
Natamycin	0.81	1.10	0.96
Ibuprofen Lysine (NeoProfen)	0.90	1.02	0.96
Vinorelbine (Navelbine)	0.85	0.77	0.81
Telaprevir (VX-950)	0.89	0.91	0.90
Cetirizine DiHCl	1.01	0.91	0.96
Dexamethasone (DHAP)	0.84	0.97	0.91
Nebivolol	0.98	0.90	0.94
Pimobendan	0.78	0.90	0.84
Pomalidomide	0.90	1.09	1.00
Tazarotene	0.67	0.81	0.74
Candesartan	0.95	0.94	0.95
Ubenimex (Bestatin)	0.82	0.95	0.89
Apixaban	0.96	1.01	0.98
Reserpine	0.90	0.99	0.94
Furosemide	0.93	0.98	0.95
Olmesartan Medoxomil	0.99	0.97	0.98
Cefdinir	0.96	0.96	0.96
Clotrimazole	0.94	1.14	1.04
Rizatriptan Benzoate	0.92	1.06	0.99
Pyridostigmine Bromide	0.95	1.12	1.03
Metolazone	0.84	1.02	0.93
Cefoperazone	1.04	0.94	0.99
Silodosin	1.02	0.93	0.97
Riluzole	0.92	1.05	0.98
Risperidone	0.90	0.84	0.87
Sulfapyridine	0.99	0.94	0.96
Sulfameter	0.99	1.04	1.01
Prilocaine	0.91	1.02	0.96
Darunavir Ethanolate	1.00	1.06	1.03
Prednisone	0.87	1.05	0.96
Alendronate (Fosamax)	0.90	1.03	0.97
Ethinyl Estradiol	0.97	0.96	0.96
Naproxen	1.03	0.97	1.00
Nitazoxanide	1.09	1.15	1.12
Triamcinolone Acetonide	1.20	0.96	1.08
Orlistat	1.02	0.98	1.00
Allopurinol	0.95	0.98	0.97
Zafirlukast	1.05	0.89	0.97
Erythromycin	0.89	1.10	0.99
Amphotericin B	0.93	1.03	0.98
Ibuprofen	1.12	0.99	1.05
Amprenavir	0.89	0.97	0.93
Albendazole	1.07	1.11	1.09
Chlorothiazide	0.99	0.98	0.99
Methyldopa (Aldomet)	1.03	0.87	0.95
Ursodiol	1.00	1.14	1.07
Nitrofural	0.96	0.97	0.96
Ketoprofen	1.00	1.24	1.12
Ketorolac	1.05	1.13	1.09
Adenosine	1.07	1.18	1.12
Zolmitriptan	1.07	0.90	0.99
Telbivudine	1.03	0.95	0.99
Monobenzone	1.00	1.10	1.05
Tretinoin	0.81	0.90	0.85

Phenylbutazone	0.98	1.04	1.01	
Ezetimibe	1.01	0.93	0.97	
Enalaprilat Dihydrate	1.04	1.08	1.06	
Dofetilide	1.13	1.09	1.11	
Isradipine	1.18	1.02	1.10	
Estrone	1.01	1.04	1.03	
Trichlormethiazide	1.06	0.97	1.02	
Loteprednol etabonate	1.02	0.94	0.98	
(6-) ε- ? Aminocaproic acid	0.97	1.03	1.00	
Aminoglutethimide	1.01	0.98	1.00	
Aminophylline	0.96	1.00	0.98	
Amorolfine HCl	0.96	0.97	0.96	
Chloramphenicol	1.06	1.02	1.04	
Flurbiprofen	0.98	0.99	0.98	
Disulfiram	0.95	1.02	0.99	
Mesalamine	0.97	1.10	1.04	
Sulfanilamide	1.03	0.93	0.98	
Betamethasone Dipropionate (Diprolene)	1.11	1.00	1.06	
Meprednisone	0.96	0.92	0.94	
Betamethasone valerate (Betnovate)	1.00	1.07	1.04	
Praziquantel	0.98	1.02	1.00	
Busulfan	1.04	1.00	1.02	
Carbamazepine	0.80	0.79	0.80	
Hydrocortisone	1.03	0.89	0.96	
Torsemide	1.12	0.98	1.05	
Desonide	1.15	0.97	1.06	
Divalproex Sodium	0.99	0.93	0.96	
Emtricitabine	0.80	0.80	0.80	
Progesterone	0.89	0.98	0.94	
Lamivudine	1.02	0.94	0.98	
Eplerenone	0.97	0.92	0.94	
Hydrochlorothiazide	1.02	0.99	1.00	
Estradiol	1.03	0.98	1.00	
Deferasirox	0.98	1.00	0.99	
Piroxicam	1.06	1.03	1.04	
Gemcitabine	1.57	1.16	1.37	toxic
Glyburide	0.95	1.04	0.99	
Adefovir Dipivoxil	0.77	0.83	0.80	
Zalcitabine	0.86	0.93	0.89	
Azathioprine	0.89	0.98	0.93	
Indomethacin	0.97	1.00	0.99	
Paliperidone (Invega)	0.98	0.81	0.89	
Terbinafine	0.97	1.00	0.99	
Levodopa (Sinemet)	0.85	1.02	0.93	
Levonorgestrel	0.99	0.94	0.96	
Gemfibrozil	0.98	1.07	1.03	
Mitotane	0.94	1.04	0.99	
Methylprednisolone	1.01	0.90	0.96	
Meloxicam	1.07	0.92	0.99	
Mesna	0.94	0.87	0.90	
Methocarbamol	0.90	1.00	0.95	
Prednisolone	1.02	0.99	1.00	
Telmisartan	1.03	0.97	1.00	
Thiabendazole	1.05	0.94	1.00	
Guaifenesin	1.09	0.97	1.03	
Rifabutin	0.99	1.08	1.03	
Esomeprazole magnesium (Nexium)	0.92	0.93	0.92	
Nicotinic Acid	0.97	0.96	0.97	
Nimodipine	1.03	0.84	0.93	
Nisoldipine	1.02	1.07	1.04	
L-Glutamine	0.95	0.93	0.94	
Gadodiamide	0.87	0.90	0.89	

Oxybutynin (Ditropan)	1.06	0.96	1.01
Enoxacin	1.11	1.03	1.07
Pitavastatin Calcium	1.22	0.76	0.99
Rifapentine	1.08	0.86	0.97
Pyrazinamide	0.99	0.95	0.97
Quetiapine Fumarate	0.98	0.92	0.95
Rifampin	1.01	1.06	1.04
Beta Carotene	0.95	1.03	0.99
Cefditoren Pivoxil	0.96	1.02	0.99
Sulfadiazine	0.98	0.98	0.98
Chlorprothixene	0.94	0.90	0.92
Oxytetracycline (Terramycin)	1.03	0.97	1.00
Thioguanine	0.98	1.09	1.03
Toremifene Citrate	1.10	1.02	1.06
Trifluridine	0.85	0.87	0.86
Azacitidine	0.86	0.92	0.89
Vidarabine	0.97	1.00	0.98
Verteporfin (Visudyne)	0.84	1.03	0.93
Teniposide	1.20	1.15	1.17
Rifaximin	0.80	0.89	0.85
Losartan Potassium (DuP 753)	0.95	0.89	0.92
Simvastatin	1.03	1.14	1.08
Ramipril	0.92	0.97	0.95
Fenofibrate	0.87	1.05	0.96
Ranitidine	0.96	0.94	0.95
Acadesine	1.01	0.84	0.93
Acetylcholine Chloride	0.79	0.92	0.85
Acipimox	0.82	0.99	0.90
Aciclovir	0.81	0.84	0.82
Nifedipine	0.81	0.89	0.85
Amiloride HCl	0.89	0.88	0.88
Amlodipine besylate (Norvasc)	0.90	0.96	0.93
Chlorpheniramine Maleate	0.79	0.92	0.85
Clofibrate (Atromid-S)	1.06	0.97	1.02
Erdosteine	0.92	0.93	0.93
Betaxolol hydrochloride (Betoptic)	0.92	0.99	0.96
Proparacaine HCl	0.83	0.86	0.84
Pranlukast	0.93	0.87	0.90
Oxfendazole	0.75	0.98	0.87
Carvedilol	0.85	0.89	0.87
Atracurium Besylate	1.02	0.99	1.00
Butoconazole nitrate	0.92	1.04	0.98
Azithromycin	0.87	1.07	0.97
Albendazole Oxide	0.98	0.93	0.96
Chloroxine	0.84	0.67	0.75
Lomustine	0.77	0.95	0.86
Chenodeoxycholic Acid	0.85	1.03	0.94
Cimetidine	1.00	0.91	0.95
Clemastine Fumarate	0.86	0.91	0.88
Curcumin	0.78	0.85	0.82
Daidzein	0.96	1.02	0.99
Oxibendazole	1.01	0.95	0.98
Penicillamine (Cuprimine)	0.92	0.98	0.95
Bifonazole	0.84	0.99	0.92
Metoprolol Tartrate	1.00	0.96	0.98
Etidronate (Didronel)	0.84	0.98	0.91
Diethylstilbestrol	0.88	0.99	0.93
Diltiazem HCl	0.93	0.97	0.95
Diphenhydramine HCl	0.92	0.91	0.91
Dapoxetine HCl	0.87	0.88	0.87
Risedronic acid (Actonel)	1.00	0.84	0.92
Tranexamic Acid	1.08	0.90	0.99

Valaciclovir HCl	0.76	1.02	0.89
Ganciclovir	0.75	0.97	0.86
Protionamide	0.94	1.02	0.98
Idoxuridine	0.86	0.95	0.90
Sparfloxacin	0.78	0.86	0.82
Felodipine	0.78	0.90	0.84
Deflazacort	0.70	1.01	0.85
Nizatidine	0.90	0.94	0.92
Carbidopa	0.80	0.88	0.84
Valsartan	0.89	0.97	0.93
Dipyridamole	0.65	0.66	0.66
Hydroxyurea	0.92	1.01	0.97
Tropisetron	0.88	0.98	0.93
Nicotinamide (Vitamin B3)	0.89	0.93	0.91
Talc	1.04	1.00	1.02
Gabapentin Hydrochloride	0.90	0.98	0.94
Diclofenac Sodium	0.95	0.94	0.95
Avobenzone	0.79	0.88	0.83
Amlodipine	0.91	1.08	1.00
Metronidazole	0.81	0.85	0.83
Flutamide	0.86	1.00	0.93
Fluvastatin Sodium	1.07	0.87	0.97
Disodium Cromoglycate	0.85	0.92	0.88
Tropicamide	0.87	0.99	0.93
Pregnenolone	0.81	0.80	0.81
Sulfamethoxazole	0.92	0.86	0.89
Sulfisoxazole	0.97	0.94	0.95
Crystal Violet	1.00	1.02	1.01
Haloperidol	0.89	0.96	0.93
Phenindione	0.90	0.96	0.93
Alibendol	0.85	1.08	0.96
Irsogladine	1.02	1.20	1.11
Nystatin (Fungicidin)	0.95	0.93	0.94
Isoniazid	0.90	0.99	0.94
Resveratrol	0.92	1.00	0.96
Levofloxacin	0.89	0.98	0.94
Enalapril Maleate	0.88	1.03	0.95
Menadione	0.76	0.80	0.78
Metformin hydrochloride (Glucophage)	0.87	1.00	0.94
Methoxsalen	0.87	1.02	0.95
Miconazole Nitrate	0.88	0.98	0.93
Sulfamethizole	0.94	1.04	0.99
Tolfenamic Acid	0.84	1.00	0.92
Pranoprofen	0.93	1.04	0.99
Sulphadimethoxine	0.93	0.84	0.89
Rimantadine	0.76	1.02	0.89
Primidone	0.99	0.77	0.88
Nefiracetam	0.89	1.03	0.96
Nicorandil	1.02	1.05	1.03
Tamoxifen Citrate	0.92	1.11	1.02
Meglumine	0.94	0.98	0.96
Aripiprazole	0.85	1.04	0.95
Methscopolamine	0.84	1.02	0.93
Amiodarone HCl	0.91	1.00	0.96
Adenine	1.00	0.85	0.92
Adenine sulfate	0.89	1.00	0.94
Adenine HCl	0.90	0.98	0.94
Ticlopidine HCl	0.92	1.10	1.01
ATP (Adenosine-Triphosphate)	0.88	1.05	0.97
Meclizine 2HCl	0.75	0.97	0.86
Mometasone furoate	1.06	0.93	0.99
Propylthiouracil	0.99	1.08	1.03

Lacidipine	0.87	0.97	0.92	
Procarbazine HCl (Matulane)	0.85	1.02	0.94	
Ondansetron (Zofran)	0.79	1.05	0.92	
Liranaftate	0.87	0.99	0.93	
D-Cycloserine	0.96	1.07	1.02	
Sodium butyrate	0.91	1.11	1.01	
Sodium orthovanadate	0.91	0.98	0.95	
Elvitegravir (GS-9137, JTK-303)	1.00	1.00	1.00	
Maraviroc	0.96	1.05	1.01	
Raltegravir (MK-0518)	0.93	1.06	1.00	
Sulindac	0.78	0.82	0.80	
Taurine	0.81	0.93	0.87	
Pramipexole dihydrochloride monohydrate	0.82	0.89	0.85	
Suplatast Tosylate	0.86	0.89	0.87	
Mirtazapine	0.88	1.03	0.95	
Benidipine HCl	0.90	0.99	0.95	
Formoterol Hemifumarate	0.84	1.03	0.94	
Chlormezanone	0.95	1.01	0.98	
Ketotifen Fumarate	0.88	0.98	0.93	
Urapidil HCl	0.95	1.03	0.99	
Ciprofloxacin (Cipro)	0.84	0.86	0.85	
Lopinavir	0.96	0.94	0.95	
Uridine	0.80	0.79	0.80	
Flunarizine 2HCl	0.88	0.89	0.89	
Fenticonazole Nitrate	0.92	0.90	0.91	
Rebamipide	0.82	1.06	0.94	
Epalrestat	0.88	1.02	0.95	
Aspartame	0.89	1.21	1.05	
Candesartan Cilexetil	0.77	1.23	1.00	
Phentolamine Mesylate	0.97	0.98	0.98	
Dyclonine HCl	0.87	0.92	0.90	
Memantine HCl	0.85	0.96	0.90	
Cyproheptadine HCl	0.84	1.04	0.94	
Doxifluridine	0.91	1.02	0.96	
Pioglitazone HCl	0.81	0.93	0.87	
Lornoxicam	0.92	1.07	1.00	
Clindamycin phosphate	0.84	1.13	0.98	
Alfuzosin HCl	0.89	1.18	1.03	
Captopril	0.90	0.95	0.92	
Oxytetracycline dihydrate	0.81	1.01	0.91	
Orphenadrine Citrate	0.87	0.99	0.93	
Gimeracil	0.79	0.99	0.89	
Cyclophosphamide monohydrate	0.83	1.04	0.94	
Tolnaftate	1.01	1.06	1.04	
Terazosin HCl	0.96	1.08	1.02	
Bromhexine HCl	0.82	0.98	0.90	
Lovastatin	0.88	0.95	0.91	
Tiopronin	0.98	0.97	0.97	
Balofloxacin	0.88	1.06	0.97	
Lafutidine	1.12	1.18	1.15	
Ozagrel HCl	1.03	1.06	1.05	
Argatroban	1.04	0.87	0.96	
Mitiglinide calcium	1.03	1.05	1.04	
Mecarbinate	0.96	0.94	0.95	
Rosiglitazone HCl	1.03	0.99	1.01	
Lisinopril (Zestril)	1.05	0.94	0.99	
Atorvastatin Calcium	1.39	1.16	1.28	toxic
Famotidine	1.05	1.01	1.03	
Moexipril HCl	1.15	0.95	1.05	
Clevidipine Butyrate	1.08	1.04	1.06	
Adiphenine HCl	1.02	0.86	0.94	
Duloxetine HCl	1.09	0.96	1.02	

Trimebutine	1.03	1.02	1.02
Ivabradine HCl	0.96	0.96	0.96
Rivastigmine Tartrate	1.07	0.95	1.01
Topiramate	1.11	1.06	1.09
Dexmedetomidine HCl (Precedex)	1.06	0.98	1.02
Betaxolol	1.14	0.91	1.03
Detomidine HCl	0.93	0.84	0.89
Fosinopril sodium (Monopril)	1.10	1.04	1.07
Ambrisentan	1.10	0.94	1.02
Bexarotene	0.79	0.72	0.76
Temocapril HCl	1.11	0.99	1.05
Gabexate Mesylate	0.99	0.89	0.94
Rasagiline Mesylate	0.97	1.10	1.04
Naltrexone HCl	1.15	0.99	1.07
Levosulpiride	1.04	0.93	0.99
Azasetron HCl (Y-25130)	1.01	0.97	0.99
Mizolastine (Mizollen)	0.94	0.98	0.96
Flunixin Meglumin	1.02	0.95	0.99
Vinpocetine (Cavinton)	0.98	0.90	0.94
Lapatinib	1.00	0.95	0.98
Blonanserin (Lonasen)	1.16	1.00	1.08
Cisatracurium Besylate	1.09	1.01	1.05
Dronedarone HCl	0.91	0.96	0.94
Conivaptan HCl	1.15	0.96	1.06
Ibutilide Fumarate	0.93	1.04	0.98
Probucol	1.16	0.97	1.07
Arbidol HCl	1.09	1.04	1.07
Licofelone	0.90	0.97	0.93
Xylose	1.13	0.99	1.06
Mestranol	1.00	0.87	0.93
Naftopidil	1.06	0.88	0.97
S- (+)-Rolipram	1.09	0.92	1.01
Bazedoxifene HCl	1.06	0.92	0.99
Fudosteine	0.99	1.04	1.02
Atropine	0.86	1.07	0.97
Roflumilast	1.07	1.16	1.12
Gabapentin (Neurontin)	1.02	1.01	1.02
Sitafloxacin Hydrate	1.06	0.97	1.01
Tebipenem Pivoxil	1.07	0.97	1.02
Rosuvastatin Calcium	1.01	1.02	1.02
Dichlorphenamide	1.05	0.92	0.98
BIBR 953 (Dabigatran etexilate, Pradaxa)	1.03	0.97	1.00
Aliskiren Hemifumarate	0.97	1.02	1.00
OSI-420	1.21	1.18	1.20
DAPT (GSI-IX)	1.03	0.93	0.98
Irinotecan HCl Trihydrate	1.06	1.05	1.06
Betamethasone	0.83	0.90	0.87
TAME	1.10	0.92	1.01
Esomeprazole Sodium	1.05	0.98	1.02
Fesoterodine Fumarate	0.97	0.97	0.97
Abiraterone Acetate	1.00	0.86	0.93
Artemether	1.02	0.85	0.94
DL-Carnitine HCl	0.97	0.92	0.94
Nalidixic acid	1.01	0.94	0.97
Ammonium Glycyrrhizinate	1.08	1.00	1.04
D-Mannitol	1.12	1.06	1.09
L-carnitine	1.09	1.05	1.07
Sorbitol	1.09	0.98	1.04
10-Deacetylbaccatin-III	1.10	0.98	1.04
Paeoniflorin	1.02	0.99	1.00
Geniposide	1.06	0.41	0.73
Genipin	1.01	1.01	1.01

Geniposidic acid	1.01	1.24	1.12
Tolbutamide	1.03	1.00	1.02
Moxifloxacin HCl	0.99	1.07	1.03
Amantadine HCl	1.11	0.94	1.02
Amfebutamone HCl	1.03	0.90	0.97
Benserazide HCl	1.12	1.00	1.06
Bethanechol chloride	0.94	0.93	0.93
Chlorpromazine HCl	1.00	0.91	0.95
Clindamycin HCl	1.08	1.04	1.06
Clonidine HCl	0.94	1.01	0.97
Clozapine	1.03	0.99	1.01
Pramipexole	1.05	0.91	0.98
Domperidone	1.03	1.01	1.02
Donepezil HCl (Aricept)	1.04	0.98	1.01
Estriol	1.10	0.92	1.01
Famciclovir	0.97	1.00	0.99
Fleroxacin (Quinodis)	1.07	0.98	1.03
Fluocinolone Acetonide	1.03	1.15	1.09
Gallamine Triethiodide	1.09	0.96	1.02
Imatinib (STI571)	1.01	0.84	0.92
Itraconazole	0.95	0.99	0.97
Lincomycin HCl	1.00	1.03	1.02
Loperamide HCl	0.90	1.00	0.95
Manidipine	1.18	1.01	1.10
Cidofovir (Vistide)	1.12	0.99	1.05
Mitoxantrone HCl	1.14	1.02	1.08
Mycophenolic acid	1.01	1.01	1.01
Nateglinide	1.01	1.03	1.02
Neostigmine bromide (Prostigmin)	1.10	0.94	1.02
Nitrendipine	1.04	1.04	1.04
Novobiocin Sodium	0.98	0.85	0.92
Olanzapine	0.89	0.91	0.90
Olopatadine HCl	0.96	0.98	0.97
Oxymetazoline HCl	1.05	0.98	1.02
Ozagrel	1.00	0.92	0.96
Pancuronium dibromide	1.08	0.95	1.02
Phenoxybenzamine HCl	1.08	0.87	0.98
Propafenone HCl	0.97	0.95	0.96
Quinine HCl Dihydrate	1.05	0.92	0.99
Racecadotril	0.87	1.11	0.99
Ribavirin	1.00	1.04	1.02
Rosiglitazone maleate	0.92	0.93	0.92
Roxithromycin	0.88	0.94	0.91
Salbutamol sulfate (Albuterol)	0.98	1.00	0.99
Scopolamine HBr	1.05	0.95	1.00
Sotalol	1.01	0.83	0.92
Sulfadoxine (Sulphadoxine)	0.96	0.99	0.98
Tenoxicam	0.97	0.91	0.94
Tobramycin	0.93	0.91	0.92
Vardenafil HCl Trihydrate	1.07	0.88	0.98
Xylazine HCl	0.82	0.99	0.90
Maprotiline HCl	0.93	0.98	0.96
Naphazoline HCl	1.05	0.99	1.02
Epinephrine Bitartrate	1.02	0.81	0.91
L-Adrenaline	0.84	0.95	0.89
DL-Adrenaline	0.95	1.00	0.98
Phenytoin	1.01	0.91	0.96
Methacycline HCl	0.93	0.84	0.88
Ciclopirox	0.93	0.86	0.90
Dopamine HCl	0.93	0.75	0.84
Ritodrine HCl	0.92	0.95	0.93
Isoconazole nitrate	1.04	0.98	1.01

Econazole nitrate	1.02	0.85	0.94
Miconazole	0.94	0.92	0.93
Secnidazole	1.15	0.91	1.03
Acetanilide	0.94	0.95	0.94
Riboflavin (Vitamin B2)	0.79	0.73	0.76
Clomipramine HCl	1.00	0.85	0.93
Phenformin HCl	0.99	0.88	0.94
Ceftiofur HCl	1.15	0.98	1.07
Cefprozil hydrate (Cefzil)	0.92	0.90	0.91
Scopine	1.00	0.96	0.98
Tiotropium Bromide hydrate	1.13	0.85	0.99
Trospium chloride	0.96	0.92	0.94
Tolterodine tartrate	1.13	0.89	1.01
Sulbactam sodium	1.06	1.00	1.03
5-Aminolevulinic acid HCl	1.02	0.99	1.00
Clarithromycin	1.04	0.91	0.97
Rosiglitazone	0.80	0.78	0.79
Terbinafine HCl	1.08	0.99	1.03
Cortisone acetate	0.97	1.07	1.02
Amiloride hydrochloride dihydrate	1.04	0.85	0.94
Clomifene citrate	1.08	0.90	0.99
Hydralazine HCl	0.90	0.91	0.91
Oxacillin sodium monohydrate	0.91	0.99	0.95
Cloxacillin Sodium	0.96	1.02	0.99
Isoprenaline HCl	0.91	0.87	0.89
Medroxyprogesterone acetate	0.92	0.85	0.88
Neomycin sulfate	1.00	1.01	1.01
Phenylephrine HCl	0.88	0.92	0.90
Prednisolone acetate (Omnipred)	1.11	0.87	0.99
Streptomycin sulfate	0.85	0.84	0.85
Tetracaine HCl	0.95	0.90	0.92
Tetracycline HCl	0.93	0.83	0.88
Vancomycin HCl (Vancocin)	0.97	0.87	0.92
Xylometazoline HCl	0.98	0.86	0.92
Zidovudine	0.95	0.82	0.88
Quinapril HCl	0.97	0.81	0.89
Trazodone HCl	0.95	0.84	0.90
Thiamphenicol	0.92	0.77	0.85
Clobetasol propionate	0.99	0.76	0.88
Brompheniramine hydrogen maleate	0.99	0.78	0.89
Dimethyl Fumarate	1.23	0.87	1.05
Calcium levofolinate (Calcium Folinate)	1.26	1.25	1.26
Miglitol	1.17	0.89	1.03
Pioglitazone	1.12	0.87	1.00
Pramiracetam	0.98	0.79	0.88
Clindamycin palmitate HCl	0.99	0.84	0.91
Oseltamivir phosphate	0.90	0.82	0.86
L-Thyroxine	1.05	0.85	0.95
Gliclazide	0.91	0.78	0.84
Acemetacin	0.91	0.79	0.85
Tioxolone	1.00	0.77	0.89
Dehydroepiandrosterone (DHEA)	1.00	0.85	0.92
Idebenone	0.80	0.97	0.88
Mifepristone	0.99	0.88	0.94
Fluocinonide	0.87	0.80	0.84
Lonidamine	0.90	0.97	0.93
Ethisterone	0.95	0.76	0.85
Clorsulon	0.86	0.95	0.91
Arecoline	0.87	0.95	0.91
Noradrenaline bitartrate monohydrate	0.90	0.83	0.86
Ibrutinib (PCI-32765)	1.38	1.10	1.24
Peramivir Trihydrate	0.85	0.85	0.85

Methimazole	1.07	0.78	0.92	
Dabrafenib (GSK2118436)	0.97	0.80	0.89	
Carfilzomib (PR-171)	0.60	0.48	0.54	toxic
Cobicistat (GS-9350)	0.90	0.88	0.89	
Hygromycin B	0.87	0.58	0.73	
Carbazochrome sodium sulfonate (AC-17)	0.95	0.73	0.84	
Rivaroxaban	1.04	0.93	0.99	
Paroxetine HCl	0.79	0.92	0.86	
Zanamivir	0.91	0.85	0.88	
Zaltoprofen	1.02	0.85	0.93	
Pazopanib	0.85	0.89	0.87	
Amoxicillin	1.20	0.99	1.09	
Niflumic acid	0.88	0.84	0.86	
Ciclopirox ethanolamine	0.92	0.76	0.84	
Rimonabant	0.85	0.92	0.88	
Cabazitaxel	1.16	0.98	1.07	
Bufexamac	0.98	0.86	0.92	
Lamotrigine	0.89	0.95	0.92	
PMSF	0.87	0.92	0.90	
Fenoprofen calcium hydrate	1.01	0.92	0.97	
Niclosamide (Niclocide)	0.70	0.52	0.61	toxic
Linagliptin	0.97	0.94	0.95	
Sulfasalazine	1.02	0.91	0.96	
Daunorubicin HCl	1.02	0.96	0.99	
Pravastatin sodium	0.96	0.98	0.97	
Bepotastine Besilate	0.82	0.86	0.84	
Fosaprepitant dimeglumine salt	0.80	0.83	0.81	
Droxidopa (L-DOPS)	0.90	0.82	0.86	
Rofecoxib	0.98	0.88	0.93	
Lurasidone HCl	1.05	0.78	0.92	
Cinepazide maleate	1.11	0.89	1.00	
Azilsartan	1.01	0.91	0.96	
Solifenacin succinate	0.88	0.88	0.88	
Palonosetron HCl	0.93	0.88	0.91	
Azelnidipine	1.12	0.97	1.04	
Alverine Citrate	1.01	0.95	0.98	
Besifloxacin HCl (Besivance)	0.89	0.84	0.86	
Azilsartan Medoxomil	0.88	0.87	0.87	
Danofloxacin Mesylate	0.83	0.92	0.87	
Enrofloxacin	0.96	0.94	0.95	
Medetomidine HCl	1.03	0.88	0.95	
Diclofenac Potassium	1.27	0.97	1.12	
Amikacin sulfate	0.92	0.94	0.93	
Naloxone HCl	0.97	0.88	0.93	
(R)-baclofen	0.98	1.01	1.00	
Caspofungin Acetate	1.00	0.92	0.96	
Dexmedetomidine	0.97	1.05	1.01	
Beclomethasone dipropionate	1.26	1.24	1.25	
Atovaquone	0.84	0.92	0.88	
Acetylcysteine	0.97	0.92	0.95	
Ulipristal	1.06	1.03	1.04	
Indacaterol Maleate	1.10	0.81	0.95	
Creatinine	1.00	0.93	0.96	
Moguisteine	1.02	0.89	0.95	
Nadifloxacin	1.01	0.83	0.92	
Pidotimod	0.81	1.06	0.93	
Pyridoxine HCl	0.85	0.92	0.88	
Vitamin C	0.91	0.83	0.87	
Sulfathiazole	1.00	0.82	0.91	
Oxybutynin chloride	0.85	0.97	0.91	
Ornidazole	1.00	0.94	0.97	
Docosanol (Abreva)	0.96	0.99	0.98	

Trimethoprim	1.00	0.80	0.90	
Biotin (Vitamin B7)	0.91	0.95	0.93	
Sulfamerazine	1.01	0.97	0.99	
Sulfamethazine	1.06	0.86	0.96	
Sodium salicylate	0.97	1.01	0.99	
Methylthiouracil	0.89	0.94	0.91	
Methenamine	0.91	0.90	0.91	
Milnacipran HCl	0.92	0.94	0.93	
Darifenacin HBr	0.99	0.91	0.95	
Tripelennamine HCl	1.06	0.89	0.98	
Ibandronate sodium	0.89	0.82	0.85	
Estradiol valerate	0.98	1.03	1.01	
Articaine HCl	1.02	0.84	0.93	
Gliquidone	0.99	0.83	0.91	
Butenafine HCl	0.97	0.90	0.93	
Mepivacaine HCl	0.81	0.91	0.86	
Naftifine HCl	0.83	0.85	0.84	
Ethynodiol diacetate	0.94	0.99	0.97	
Sertaconazole nitrate	1.00	0.90	0.95	
Tylosin tartrate	0.97	0.90	0.94	
Abacavir sulfate	0.84	0.92	0.88	
Altrenogest	0.93	0.88	0.90	
Ampicillin sodium	0.84	0.87	0.86	
Anagrelide HCl	1.02	0.85	0.94	
Antipyrine	0.81	0.98	0.90	
L-Arginine HCl	0.88	0.94	0.91	
Atomoxetine HCl	0.92	0.87	0.89	
Betahistine 2HCl	0.86	0.88	0.87	
Brinzolamide	0.88	0.91	0.90	
Carbenicillin disodium	1.05	0.85	0.95	
Amitriptyline HCl	1.09	0.91	1.00	
Adrenalone HCl	0.97	0.95	0.96	
Azatadine dimaleate	0.99	0.79	0.89	
(+,-)-Octopamine HCl	0.79	0.91	0.85	
Ropinirole HCl	0.79	0.80	0.79	
Azlocillin sodium salt	0.96	0.93	0.94	
Azacyclonol	0.88	0.91	0.89	
Reboxetine mesylate	1.00	0.84	0.92	
Triflusal	0.94	0.88	0.91	
Trifluoperazine 2HCl	0.76	0.83	0.79	
Meptazinol HCl	0.89	0.89	0.89	
Fexofenadine HCl	1.07	1.06	1.07	
Amidopyrine	1.11	0.84	0.97	
Thiamine HCl (Vitamin B1)	1.01	0.79	0.90	
Moclobemide (Ro 111163)	0.93	0.87	0.90	
Pergolide mesylate	1.18	0.97	1.07	
Lithocholic acid	1.02	0.94	0.98	
Ethambutol HCl	0.92	0.87	0.89	
Doxycycline HCl	0.90	1.05	0.98	
Clinafloxacin (PD127391)	1.03	0.86	0.95	
Pemirolast (BMY 26517) potassium	0.93	1.04	0.98	
Mirabegron	1.03	0.88	0.95	
Acebutolol HCl	0.98	0.96	0.97	
Ampiroxicam	0.99	0.85	0.92	
Desloratadine	0.92	0.90	0.91	
Sodium Monofluorophosphate	0.81	0.94	0.88	
Hyoscyamine	0.95	0.94	0.94	
Cyclamic acid	0.91	0.80	0.86	
Ouabain	0.59	0.47	0.53	toxic
Allylthiourea	1.08	0.87	0.98	
Sodium Picosulfate	0.96	0.96	0.96	
Tolcapone	0.93	0.77	0.85	

Probenecid	0.99	0.88	0.94
Procaine HCl	0.89	0.98	0.93
Homatropine Bromide	0.96	0.96	0.96
Hydroxyzine 2HCl	0.90	0.96	0.93
Flavoxate HCl	1.06	1.00	1.03
Colistin Sulfate	0.97	1.06	1.01
Nevirapine	1.13	0.94	1.04
Aclidinium Bromide	1.02	1.08	1.05
Diphemanil Methylsulfate	0.94	0.98	0.96
Vitamin D2	0.96	0.99	0.98
Doxapram HCl	0.89	0.81	0.85
Dibucaine HCl	1.07	0.88	0.98
Methazolamide	0.94	0.85	0.90
Norethindrone	0.99	0.92	0.96
Olsalazine Sodium	1.05	0.96	1.01
Nafcillin Sodium	1.04	0.97	1.00
Tetrahydrozoline HCl	1.06	0.95	1.00
Toltrazuril	1.03	0.94	0.99
Bisacodyl	0.99	1.09	1.04
Carbimazole	1.05	0.90	0.98
Valdecoxib	1.02	0.98	1.00
Valganciclovir HCl	0.85	0.99	0.92
Nabumetone	0.96	0.89	0.92
Netilmicin Sulfate	0.99	0.77	0.88
Sertraline HCl	1.04	0.95	0.99
Spironolactone	0.98	0.98	0.98
Retapamulin	1.01	0.95	0.98
Methyclothiazide	0.96	1.07	1.01
Cytarabine	1.01	0.89	0.95
Erythromycin Ethylsuccinate	0.89	0.85	0.87
Ronidazole	0.98	0.97	0.98
Vitamin D3	0.84	0.89	0.87
Glipizide	0.96	0.83	0.89
Guanabenz Acetate	0.91	0.97	0.94
Dequalinium chloride	0.81	0.76	0.78
Deferiprone	1.04	0.97	1.01
Tinidazole	1.07	1.00	1.04
Hexamethonium bromide	0.99	0.93	0.96
Decamethonium Bromide	0.93	0.95	0.94
Sodium 4-Aminosalicylate	1.02	0.97	1.00
Flucytosine	0.89	1.02	0.95
Ipratropium Bromide	0.82	0.88	0.85
Propranolol HCl	0.90	0.84	0.87
Mequinol	1.04	0.96	1.00
Mefenamic Acid	0.75	0.93	0.84
Ticagrelor	0.95	1.01	0.98
Triamterene	0.83	0.99	0.91
Sulfacetamide Sodium	1.01	0.89	0.95
Lomerizine HCl	1.00	0.95	0.98
Levobetaxolol HCl	0.82	0.98	0.90
Loxapine Succinate	0.93	0.85	0.89
Oxymetholone	0.88	0.95	0.92
Flumethasone	0.79	0.79	0.79
Halobetasol Propionate	0.83	0.66	0.74
Fenspiride HCl	1.03	1.10	1.06
Indapamide	1.07	1.01	1.04
Pramoxine HCl	1.11	0.97	1.04
Bismuth Subcitrate Potassium	1.11	0.95	1.03
Difluprednate	1.05	1.00	1.03
Droperidol	1.07	1.00	1.03
Dydrogesterone	1.00	0.96	0.98
Dexlansoprazole	0.92	0.83	0.88

Esmolol HCl	0.81	0.76	0.78
Voglibose	1.01	0.90	0.95
Eprosartan Mesylate	1.00	0.98	0.99
Diminazene Aceturate	0.99	0.95	0.97
Suprofen	0.91	0.89	0.90
Ethionamide	0.84	0.94	0.89
Clodronate Disodium	0.96	0.97	0.97
Dicloxacillin Sodium	0.92	0.92	0.92
Triclabendazole	0.91	0.86	0.88
Didanosine	1.07	0.93	1.00
Isovaleramide	0.97	0.94	0.96
Histamine Phosphate	0.95	0.94	0.95
Sulconazole Nitrate	1.10	0.92	1.01
Tilmicosin	1.14	0.92	1.03
Troxipide	1.07	1.00	1.03
Ranolazine	1.02	0.87	0.95
Fenoprofen Calcium	0.88	1.05	0.97
Clorprenaline HCl	0.90	0.94	0.92
Carprofen	0.91	0.94	0.93
Eprazinone 2HCl	0.95	0.94	0.95
Dropropizine	1.05	0.93	0.99
Amprolium HCl	0.92	0.91	0.91
Decoquinate	0.98	1.08	1.03
Bacitracin	0.96	1.05	1.00
Azithromycin Dihydrate	1.04	0.93	0.99
Ampicillin Trihydrate	0.86	0.97	0.92
Orbifloxacin	0.83	1.02	0.92
Penfluridol	0.91	0.95	0.93
Ethamsylate	0.85	0.82	0.84
D-Phenylalanine	1.05	0.92	0.98
Flubendazole	0.74	0.95	0.84
Chlorzoxazone	0.73	0.94	0.83
Chlortetracycline HCl	0.79	0.80	0.79
Chloroquine Phosphate	0.88	1.03	0.96
Roxatidine Acetate HCl	0.95	0.99	0.97
Bezafibrate	0.98	1.09	1.04
Benzoic Acid	0.83	1.00	0.92
Benzethonium Chloride	0.81	0.87	0.84
Doxofylline	0.99	0.93	0.96
Benzydamine HCl	0.80	1.01	0.91
Chlorpropamide	0.93	0.94	0.94
Cyromazine	0.89	0.95	0.92
Pefloxacin Mesylate	0.83	0.92	0.87
Coumarin	0.92	1.01	0.97
Choline Chloride	0.87	0.97	0.92
Cetylpyridinium Chloride	0.99	0.94	0.96
Sodium Gluconate	0.85	0.85	0.85
Sulfaguanidine	0.89	0.93	0.91
Trometamol	0.78	0.79	0.78
Uracil	0.82	0.97	0.89
Climbazole	0.87	1.05	0.96
Mezlocillin Sodium	0.84	0.91	0.88
Nefopam HCl	0.87	1.02	0.95
Nicardipine HCl	0.95	0.87	0.91
Nifuroxazide	0.85	0.87	0.86
Paromomycin Sulfate	1.04	0.92	0.98
Tiratricol	0.88	1.11	0.99
Domiphen Bromide	0.98	0.94	0.96
Salicylanilide	0.87	0.96	0.91
Sasapyrine	0.87	1.08	0.97
Cyclandelate	0.86	1.10	0.98
Cinchophen	0.86	1.02	0.94

Betamipron	0.88	0.94	0.91	
Chlorquinaldol	0.92	0.87	0.89	
Azaguanine-8	0.90	1.00	0.95	
Broxyquinoline	0.90	0.85	0.88	
Bemegride	1.06	0.85	0.95	
Aminothiazole	0.93	0.96	0.95	
Antazoline HCl	0.82	1.00	0.91	
Tolperisone HCl	0.90	0.92	0.91	
Florfenicol	0.79	1.02	0.91	
Furaltadone HCl	0.87	0.91	0.89	
Isosorbide	0.93	1.01	0.97	
Dibenzothiophene	0.85	0.97	0.91	
Cysteamine HCl	0.97	0.89	0.93	
Clofibric Acid	0.92	1.02	0.97	
Chlorocresol	0.72	0.87	0.79	
Benzocaine	0.87	1.01	0.94	
Montelukast Sodium	0.86	0.93	0.90	
Dirithromycin	1.05	0.93	0.99	
Sucralose	1.08	0.89	0.99	
Valnemulin HCl	0.98	1.01	1.00	
Liothyronine Sodium	0.82	0.90	0.86	
Amoxapine	0.97	0.91	0.94	
Azaperone	0.95	1.06	1.00	
Benzbromarone	0.69	0.75	0.72	toxic
Mevastatin	0.94	0.95	0.95	
Mexiletine HCl	1.07	1.05	1.06	
Minocycline HCl	0.83	1.04	0.94	
Fidaxomicin	0.96	0.91	0.93	
Fluorometholone Acetate	0.94	0.84	0.89	
Oxybuprocaine HCl	0.91	0.95	0.93	
Oxaprozin	0.93	1.15	1.04	
Pilocarpine HCl	0.93	1.02	0.98	
Nithiamide	0.81	1.02	0.92	
Zoxazolamine	1.17	1.00	1.09	
Phenazopyridine HCl	0.95	0.81	0.88	
Triamcinolone	1.05	0.92	0.99	
Primaquine Diphosphate	1.17	0.84	1.01	
Cepharanthine	1.08	1.12	1.10	
Bergapten	1.02	1.01	1.02	
Doxylamine Succinate	1.16	1.07	1.12	
Cetrimonium Bromide (CTAB)	1.03	0.86	0.95	
Deoxycorticosterone acetate	1.16	1.02	1.09	
Serotonin HCl	1.15	0.96	1.05	
Sodium ascorbate	1.14	0.94	1.04	
Potassium Iodide	0.99	0.95	0.97	
Alexidine HCl	0.38	0.41	0.40	toxic
9-Aminoacridine	0.89	1.08	0.98	
Anisindione	0.93	0.97	0.95	
Anisotropine Methylbromide	0.95	0.84	0.89	
Auranofin	0.83	0.70	0.77	toxic
Benzthiazide	1.20	0.90	1.05	
Bromocriptine Mesylate	0.87	1.06	0.97	
Butacaine	1.28	1.26	1.27	
Calcium Gluceptate	1.22	1.00	1.11	
Ceftazidime Pentahydrate	1.14	0.88	1.01	
Clinafloxacin HCl	1.28	0.89	1.09	
Clopamide	1.08	0.96	1.02	
Clorgyline HCl	1.12	0.96	1.04	
Colistimethate Sodium	0.93	0.91	0.92	
Dibenzepine HCl	1.09	0.98	1.04	
Dimaprit 2HCl	0.99	0.89	0.94	
Diphenylpyraline HCl	1.11	0.90	1.01	

Disopyramide Phosphate	1.14	0.94	1.04	
Emetine	0.51	0.53	0.52	toxic
Ethoxzolamide	1.13	1.00	1.06	
Famprofazone	1.08	0.92	1.00	
Guanethidine Sulfate	1.06	0.89	0.98	
Hemicholinium Bromide	1.13	0.90	1.02	
Isoetharine Mesylate	0.78	0.77	0.77	
Meclocycline Sulfosalicylate	1.02	0.96	0.99	
Medrysone	1.24	1.03	1.14	
Mepiroxol	0.91	0.95	0.93	
Mesoridazine Besylate	1.02	1.02	1.02	
Metaproterenol Sulfate	1.36	1.15	1.25	toxic
Methapyrilene HCl	1.01	1.00	1.01	
Methoxamine HCl	1.09	0.96	1.03	
Meticrane	1.04	0.92	0.98	
Moxalactam Disodium	1.05	0.91	0.98	
Nalmefene HCl	1.05	0.97	1.01	
Nialamide	1.24	1.00	1.12	
Oxethazaine	1.06	1.00	1.03	
Oxprenolol HCl	1.10	1.02	1.06	
Pentoxifylline	1.11	1.09	1.10	
Physostigmine Salicylate	1.09	0.94	1.02	
Piromidic Acid	1.04	0.97	1.01	
Procyclidine HCl	1.22	0.91	1.07	
R-(-)-Apomorphine HCl Hemihydrate	0.75	0.69	0.72	
Ractopamine HCl	1.30	1.27	1.28	
Rolitetracycline	1.00	0.91	0.95	
Terfenadine	1.13	1.07	1.10	
Thiostrepton	1.05	0.94	1.00	
Thonzonium Bromide	1.32	1.20	1.26	
Tolazamide	1.22	0.99	1.11	
Tacrine HCl	1.19	1.05	1.12	
Carbachol	1.06	0.96	1.01	
Tolmetin Sodium	1.04	0.95	0.99	
Cinoxacin	1.02	0.98	1.00	
Glafenine HCl	0.93	1.03	0.98	
Noscapine HCl	1.04	1.05	1.04	
Phenothrin	1.20	0.88	1.04	
Phthalylsulfacetamide	1.25	1.04	1.14	
Pinacidil	1.19	1.05	1.12	
Suxibuzone	1.05	1.02	1.04	
Carbenoxolone Sodium	0.93	1.06	1.00	
Tioconazole	1.07	0.92	0.99	
Pridinol Methanesulfonate	1.12	0.94	1.03	
Triflupromazine HCl	1.11	0.95	1.03	
Dicyclomine HCl	1.04	1.00	1.02	
Thioridazine HCl	1.29	1.28	1.28	
Mepenzolate Bromide	0.93	0.96	0.95	
Aceclidine HCl	1.06	0.96	1.01	
Imipramine HCl	1.12	0.95	1.03	
Methylhydantoin-5-(D)	1.36	1.22	1.29	
Metrizamide	1.28	1.28	1.28	
Proadifen HCl	1.18	0.98	1.08	
Pyrilamine Maleate	0.92	0.98	0.95	
Bekanamycin	1.09	0.95	1.02	
Difloxacin HCl	1.00	0.99	0.99	
Fosfomycin Tromethamine	1.08	1.01	1.05	
Azelastine HCl	1.11	1.12	1.12	
Bendroflumethiazide	1.15	0.94	1.05	
Bentiromide	1.04	1.08	1.06	
Bephenium Hydroxynaphthoate	0.96	0.95	0.95	
Guanidine HCl	1.00	0.98	0.99	

Potassium Canrenoate	0.93	0.94	0.94
Cephapirin Sodium	1.07	0.95	1.01
Clofoctol	0.93	1.06	1.00
Dichlorisone Acetate	1.13	1.06	1.09
Digoxigenin	1.12	0.92	1.02
Diperodon HCl	1.12	1.03	1.08
Isoxicam	0.91	1.03	0.97
Lithium Citrate	0.88	0.97	0.93
Lofexidine HCl	1.03	1.02	1.03
Nifenazone	0.92	0.94	0.93
Sulbactam	0.96	1.01	0.99
Pasiniazid	0.98	1.06	1.02
Picrotoxinin	1.03	0.98	1.00
Pindolol	1.03	0.97	1.00
Prochlorperazine Dimaleate	1.13	0.96	1.04
Procodazole	0.88	0.97	0.93
Quipazine Maleate	1.09	1.10	1.09
Diclofenac Diethylamine	1.25	1.05	1.15
Phosphatidylcholine	0.97	1.09	1.03
Spiramycin	1.27	1.27	1.27
Sodium 4-aminohippurate Hydrate	1.02	0.94	0.98
Misoprostol	0.70	0.85	0.78
Trimipramine Maleate	0.81	0.95	0.88
Eltrombopag	1.17	0.97	1.07
Tacrolimus (FK506)	0.97	1.01	0.99
Pimecrolimus	1.00	1.06	1.03
Plerixafor (AMD3100)	0.81	0.95	0.88
2-Thiouracil	0.87	1.09	0.98
Pentamidine	0.90	1.05	0.97
Clofazimine	1.01	1.18	1.09
Sarafloxacin HCl	1.06	1.03	1.04
Nimesulide	1.08	0.98	1.03
Almotriptan Malate	0.84	1.00	0.92
Cephalomannine	1.13	1.06	1.09
D-Pantothenic acid	1.06	0.98	1.02
Amoxicillin Sodium	1.12	1.03	1.08
Clindamycin	1.00	1.10	1.05
Dexamethasone acetate	1.01	0.97	0.99
Avanafil	1.07	1.09	1.08
Deoxyarbutin	0.95	0.99	0.97
Fluticasone propionate	0.89	0.95	0.92
Cytidine	1.08	0.96	1.02
Imidapril HCl	1.02	0.89	0.95
Bupivacaine HCl	1.00	0.92	0.96
Spectinomycin HCl	0.99	1.02	1.00
Phenacetin	0.95	1.06	1.01
Aspirin	0.93	1.03	0.98
Benztropine mesylate	1.00	0.99	0.99
Bismuth Subsalicylate	1.07	1.05	1.06
Amfenac Sodium Monohydrate	1.14	1.10	1.12
Pyrimethamine	1.05	1.04	1.04
Moxonidine	1.00	1.04	1.02
Dextrose	1.05	1.04	1.05
Fenbendazole	1.11	0.89	1.00
Phenytoin sodium	1.00	0.97	0.99
Tolvaptan	1.12	1.01	1.06
Bindarit	0.89	0.98	0.94
Flumequine	1.13	0.99	1.06
Pheniramine Maleate	0.99	0.98	0.99
Penicillin G Sodium	1.12	1.08	1.10
Ginkgolide A	0.97	0.89	0.93
Cilazapril Monohydrate	0.84	0.92	0.88

Dabigatran Etexilate	1.09	0.99	1.04
Moroxydine HCl	0.94	1.05	0.99
Lomefloxacin HCl	0.87	1.10	0.98
Buflomedil HCl	1.03	0.95	0.99
Otilonium Bromide	1.06	0.98	1.02
Catharanthine	0.94	0.94	0.94
Ropivacaine HCl	1.21	1.00	1.11
1-Hexadecanol	0.95	1.11	1.03
Penciclovir	0.88	1.06	0.97
Pipenzolate Bromide	0.88	1.12	1.00
Ethacridine lactate monohydrate	0.77	0.93	0.85
Pimozide	0.92	1.02	0.97
Chromocarb	1.06	0.96	1.01
Hydrastinine HCl	0.88	1.02	0.95
Piperacillin Sodium	0.87	0.91	0.89
Nomifensine Maleate	0.83	0.86	0.85
Benfotiamine	1.13	0.99	1.06
Camylofin Chlorhydrate	0.92	0.90	0.91
Carbadox	1.15	1.07	1.11
Oxeladin Citrate	1.12	0.95	1.04
Physostigmine Hemisulfate Salt	1.06	1.20	1.13
Metaraminol Bitartrate	0.87	0.99	0.93

1) Two independent experiments were performed.

2) Red indicates more than 1.25 while green indicates less than 0.80.

3) Toxicity was detemined by microscopic observation.

Supplementary Table 3. Summary of the second screening

Compounds	Targets/Remarks	Relative GeoMean of HLA-DR[1),2)]		
		n = 1	n = 2	mean
Chloroxine	Quinoline derivative	1.56	1.59	1.57
Dipyridamole	Phosphodiesterase V inhibitor	1.57	1.47	1.52
Alprostadil	Prostaglandin E1	1.27	1.30	1.28
Emtricitabine	HIV reverse transcriptase inhibitor	1.32	1.24	1.28
Chlortetracycline HCl	Tetracycline analogue	1.29	1.26	1.27
Carbamazepine	Na+ channel blockers	1.33	1.22	1.27
Trometamol	pH buffering agent, also known as Tris	1.26	1.28	1.27
Chlorocresol	Cresol derivative	1.26	1.25	1.26
Isoetharine Mesylate	β2 adrenergic receptor agonist	1.08	1.07	1.08
Esmolol HCl	β1 adrenergic receptor antagonist	1.02	1.08	1.05
Uridine	Pyrimidine synthesis promotion	0.98	1.02	1.00
Isotretinoin	Retinoic acid receptor agonist	1.02	0.93	0.98
Geniposide	Iridoid glycoside from gardenia	0.79	1.13	0.96
Adefovir Dipivoxil	HBV reverse transcriptase inhibitor	1.06	0.84	0.95
Hygromycin B	Aminoglycoside	0.86	1.01	0.94
Riboflavin	Vitamin B2	0.73	1.13	0.93
Misoprostol	Prostaglandin E1	1.08	0.78	0.93
Menadione	Vitamin K	0.87	0.94	0.91
Tazarotene	Retinoic acid receptor agonist	0.86	0.92	0.89
Bexarotene	Retinoid X receptor agonist	0.80	0.95	0.88
Acitretin	Retinoic acid receptor agonist	0.79	0.80	0.79
Sunitinib Malate	Multi-kinase inhibitor	0.85	0.70	0.77
R-(-)-Apomorphine HCl Hemihydrate	Dopamine receptor agonist	0.70	0.82	0.76
Sulindac	Cyclooxidase inhibitor	0.77	0.74	0.76
Ropinirole HCl	Dopamine D2 receptor agonist	0.71	0.75	0.73
Rosiglitazone	Peroxisome proliferator-activated receptor γ agonist	0.69	0.76	0.73
Everolimus (RAD001)	Mechanistic target of rapamycin inhibitor	0.66	0.76	0.71
Trifluoperazine 2HCl	Dopamine D2 receptor antagonist	0.53	0.80	0.67
Calcitriol	Vitamin D receptor agonist	0.56	0.62	0.59
Calcifediol	Vitamin D receptor agonist	0.50	0.67	0.59
Dequalinium chloride	Quinoline derivative, quaternary ammonium	0.27	0.75	0.51
Sorafenib Tosylate	Multi-kinase inhibitor	0.24	0.44	0.34
Halobetasol Propionate	Glucocorticoid receptor agonist	0.20	0.22	0.21
Flumethasone	Glucocorticoid receptor agonist	0.08	0.23	0.16

1) Two independent experiments were performed.

2) Red indicates more than 1.25 while blue indicates less than 0.80.

Supplementary Table 4. DEGs in LC cells and macrophages after HX531 treatment

Gene	HLF cells		THP-1-derived macrophages		UP/DOWN
	fold changes[1),2)]	adjusted P values	fold changes[1),2)]	adjusted P values	
AARS1	1.59	0.022	2.73	0.019	Mac UP
ABCA10	2.87	0.043	1.50	0.676	LC UP
ABCC3	1.70	0.194	2.40	0.038	Mac UP
ABLIM3	2.39	0.023	2.41	0.059	LC UP
ACKR3	0.84	0.797	0.49	0.032	Mac DOWN
ACSL1	2.31	0.136	2.23	0.037	Mac UP
ADM2	1.61	0.088	13.06	0.043	Mac UP
ADORA3	0.42	0.646	0.32	0.043	Mac DOWN
AKAP5	0.82	0.510	2.37	0.038	Mac UP
ALDH1L2	3.78	0.395	13.30	0.024	Mac UP
ALPK2	0.75	0.243	2.41	0.050	Mac UP
ALS2CL	0.88	0.540	2.09	0.011	Mac UP
ANKRD37	0.42	0.008	0.44	0.225	LC DOWN
ANLN	0.79	0.120	0.30	0.029	Mac DOWN
APBB1	0.80	0.269	0.47	0.048	Mac DOWN
APOC1	0.44	0.299	0.26	0.040	Mac DOWN
APOC2	0.46	0.260	0.28	0.027	Mac DOWN
APOC4-APOC2	0.76	0.707	0.42	0.038	Mac DOWN
AQP1	0.47	0.381	0.27	0.027	Mac DOWN
ARAP2	1.36	0.238	3.09	0.044	Mac UP
ARHGAP11A	0.87	0.711	0.46	0.016	Mac DOWN
ARHGAP18	0.94	0.736	0.43	0.037	Mac DOWN
ARHGDIB	0.52	0.187	0.38	0.011	Mac DOWN
ARHGEF35	1.01	0.920	2.36	0.047	Mac UP
ARHGEF5	0.95	0.671	2.23	0.021	Mac UP
ARVCF	0.88	0.611	2.86	0.037	Mac UP
ASF1B	0.34	0.160	0.40	0.012	Mac DOWN
ASNS	2.69	0.016	15.27	0.030	Common UP
ATAD2	0.83	0.130	0.44	0.043	Mac DOWN
ATF4	1.41	0.057	2.45	0.011	Mac UP
ATF5	1.05	0.718	3.22	0.013	Mac UP
ATP8B4	0.25	0.173	0.28	0.040	Mac DOWN
AURKA	0.79	0.240	0.49	0.011	Mac DOWN
AURKB	0.81	0.537	0.31	0.016	Mac DOWN
BBS2	1.14	0.059	2.04	0.015	Mac UP
BCL2L1	0.41	0.007	0.64	0.116	LC DOWN
BEND6	1.28	0.264	2.23	0.025	Mac UP
BEST1	1.41	0.395	2.22	0.018	Mac UP
BIRC5	0.79	0.222	0.39	0.018	Mac DOWN
BIRC7	0.75	0.611	2.42	0.038	Mac UP
BLM	0.78	0.632	0.42	0.046	Mac DOWN
BRPF3	1.22	0.056	2.01	0.033	Mac UP
BST2	0.08	0.102	0.43	0.025	Mac DOWN
BUB1B	0.66	0.436	0.36	0.032	Mac DOWN
BVES	1.51	0.400	2.27	0.015	Mac UP
C1QTNF6	0.69	0.136	3.56	0.017	Mac UP
C1RL-AS1	1.83	0.824	3.18	0.024	Mac UP
CACNA1F	2.04	0.629	2.19	0.042	Mac UP
CACNA1I	0.07	0.026	0.70	0.647	LC DOWN
CAPN5	1.11	0.531	2.58	0.018	Mac UP
CARF	1.37	0.229	2.61	0.035	Mac UP
CARS1	1.26	0.088	2.66	0.015	Mac UP
CBS	1.36	0.089	3.23	0.029	Mac UP
CCDC112	1.42	0.187	2.10	0.034	Mac UP
CCDC15-DT	1.17	0.785	10.01	0.040	Mac UP
CCDC154	0.71	0.764	2.85	0.026	Mac UP
CCDC24	1.14	0.336	2.13	0.020	Mac UP

CCL20	0.78	0.190	**0.42**	**0.012**	Mac DOWN
CCL4	0.63	0.500	**0.41**	**0.038**	Mac DOWN
CCL7	0.25	0.387	**0.31**	**0.037**	Mac DOWN
CCNA2	0.91	0.706	**0.41**	**0.015**	Mac DOWN
CCNB1	0.90	0.612	**0.38**	**0.034**	Mac DOWN
CCNB2	0.96	0.830	**0.42**	**0.042**	Mac DOWN
CCNE2	0.57	0.143	**0.26**	**0.048**	Mac DOWN
CCNF	0.97	0.735	**0.42**	**0.016**	Mac DOWN
CCPG1	1.38	0.254	**2.19**	**0.015**	Mac UP
CCSER2	1.39	0.128	**2.22**	**0.021**	Mac UP
CD14	0.64	0.400	**0.47**	**0.045**	Mac DOWN
CD163	3.10	0.322	**0.38**	**0.014**	Mac DOWN
CD36	0.50	0.231	**0.47**	**0.040**	Mac DOWN
CD74	**0.38**	**0.036**	0.69	0.193	LC DOWN
CD86	0.65	0.728	**0.34**	**0.014**	Mac DOWN
CDC20	0.90	0.743	**0.49**	**0.037**	Mac DOWN
CDC25A	0.71	0.327	**0.38**	**0.030**	Mac DOWN
CDC25C	0.68	0.347	**0.35**	**0.027**	Mac DOWN
CDC45	0.34	0.082	**0.41**	**0.041**	Mac DOWN
CDC7	1.06	0.672	**0.40**	**0.018**	Mac DOWN
CDCA3	0.77	0.406	**0.36**	**0.046**	Mac DOWN
CDCA5	0.81	0.278	**0.41**	**0.018**	Mac DOWN
CDCA8	0.98	0.913	**0.40**	**0.018**	Mac DOWN
CDK1	0.78	0.138	**0.30**	**0.012**	Mac DOWN
CDKN2D	0.66	0.122	**0.49**	**0.025**	Mac DOWN
CDKN3	0.70	0.332	**0.37**	**0.035**	Mac DOWN
CDT1	0.67	0.059	**0.44**	**0.016**	Mac DOWN
CEBPG	1.61	0.062	**2.32**	**0.021**	Mac UP
CELF2	0.45	0.466	**0.48**	**0.047**	Mac DOWN
CENPA	1.30	0.445	**0.41**	**0.025**	Mac DOWN
CENPE	0.89	0.672	**0.49**	**0.049**	Mac DOWN
CENPQ	1.18	0.591	**0.46**	**0.016**	Mac DOWN
CENPW	0.99	0.990	**0.39**	**0.050**	Mac DOWN
CFAP77			**720.77**[3)]	**0.034**	Mac UP
CHAC1	1.60	0.101	**16.46**	**0.050**	Mac UP
CHRNA5	0.74	0.203	**0.33**	**0.020**	Mac DOWN
CKMT2-AS1	1.37	0.297	**2.02**	**0.026**	Mac UP
CKS2	1.04	0.780	**0.44**	**0.020**	Mac DOWN
CLCN5	1.83	0.066	**2.14**	**0.021**	Mac UP
CLGN	4.26	0.302	**9.05**	**0.044**	Mac UP
CLIP4	1.43	0.118	**2.21**	**0.039**	Mac UP
CLSPN	0.67	0.095	**0.37**	**0.029**	Mac DOWN
CMKLR1	0.00	0.615	**0.37**	**0.038**	Mac DOWN
CMPK2	**0.09**	**0.018**	0.10	0.099	LC DOWN
COPB2-DT	1.57	0.484	**2.40**	**0.024**	Mac UP
CORO7	0.72	0.089	**0.48**	**0.037**	Mac DOWN
CPEB1-AS1	0.66	0.646	**3.04**	**0.027**	Mac UP
CSRP1-AS1	1.15	0.732	**2.67**	**0.042**	Mac UP
CST1	0.26	0.209	**0.03**	**0.030**	Mac DOWN
CTNNA3	1.74	0.837	**5.21**	**0.040**	Mac UP
CXCL10	**0.10**	**0.021**	0.18	0.246	LC DOWN
CXCL11	**0.14**	**0.032**	0.05	0.662	LC DOWN
CYP19A1	0.90	0.741	**0.45**	**0.045**	Mac DOWN
CYP27A1	1.08	0.565	**2.93**	**0.034**	Mac UP
CYP4V2	1.55	0.142	**3.01**	**0.039**	Mac UP
D2HGDH	1.03	0.427	**2.00**	**0.024**	Mac UP
DCAF10	1.66	0.032	**2.76**	**0.013**	Mac UP
DENND2B	1.02	0.766	**0.40**	**0.042**	Mac DOWN
DENND2D	0.58	0.517	**2.01**	**0.012**	Mac UP
DEPDC1B	0.74	0.562	**0.41**	**0.027**	Mac DOWN

DEPDC7	1.01	0.974	0.37	0.050	Mac DOWN
DEPP1	0.38	0.150	0.49	0.044	Mac DOWN
DET1	1.20	0.663	3.19	0.034	Mac UP
DHRS11	1.09	0.712	2.89	0.019	Mac UP
DHRS12	1.01	0.970	2.03	0.041	Mac UP
DIAPH3	0.67	0.320	0.40	0.042	Mac DOWN
DLGAP1-AS2	2.23	0.068	2.24	0.026	Mac UP
DLGAP5	0.73	0.547	0.31	0.015	Mac DOWN
DMGDH	2.00	0.728	9.61	0.024	Mac UP
DNAI4	2.18	0.094	2.02	0.025	Mac UP
DSCC1	0.90	0.769	0.42	0.032	Mac DOWN
DTL	0.59	0.053	0.35	0.018	Mac DOWN
DTNB	1.26	0.240	2.14	0.034	Mac UP
E2F1	0.47	0.033	0.55	0.015	LC DOWN
E2F2	0.69	0.398	0.22	0.012	Mac DOWN
ECM1	0.64	0.071	0.47	0.048	Mac DOWN
ELOVL6	1.54	0.102	2.46	0.040	Mac UP
ENSG00000203644	1.33	0.229	2.16	0.031	Mac UP
ENSG00000224404	2.64	0.573	0.00	0.049	Mac DOWN
ENSG00000224810			1354.08[3)]	0.029	Mac UP
ENSG00000228703	6.39	0.514	0.00	0.049	Mac DOWN
ENSG00000230612	4.84	0.710	0.00	0.049	Mac DOWN
ENSG00000232811	0.53	0.833	0.36	0.041	Mac DOWN
ENSG00000233478			348.23[3)]	0.033	Mac UP
ENSG00000236352	1.38	0.853	4.44	0.037	Mac UP
ENSG00000238178			0.18	0.033	Mac DOWN
ENSG00000253821	0.00	0.651	0.41	0.047	Mac DOWN
ENSG00000254587			345.28[3)]	0.033	Mac UP
ENSG00000255491	0.24	0.059	0.44	0.036	Mac DOWN
ENSG00000256568	0.00	0.596	0.27	0.030	Mac DOWN
ENSG00000258561	1.07	0.960	5.05	0.018	Mac UP
ENSG00000259066	4442.23[3)]	0.769	9510.67[3)]	0.038	Mac UP
ENSG00000259661	0.67	0.755	11.92	0.030	Mac UP
ENSG00000260331	0.66	0.790	1891.24[3)]	0.033	Mac UP
ENSG00000261172			2.69	0.048	Mac UP
ENSG00000261625			0.00	0.049	Mac DOWN
ENSG00000266709	0.57	0.138	0.24	0.013	Mac DOWN
ENSG00000267385	0.79	0.852	0.36	0.044	Mac DOWN
ENSG00000267765	2.04	0.693	2154.28[3)]	0.033	Mac UP
ENSG00000270207			5.40	0.049	Mac UP
ENSG00000271698	26.84	0.138	11.89	0.035	Mac UP
ENSG00000272405	1.10	0.709	2.67	0.033	Mac UP
ENSG00000272449	0.00	0.630	0.37	0.032	Mac DOWN
ENSG00000272668	1.09	0.403	2.46	0.032	Mac UP
ENSG00000273192	1.10	0.756	3.91	0.045	Mac UP
ENSG00000273230	0.88	0.550	2.64	0.034	Mac UP
ENSG00000274767	1.00	1.001	2.43	0.022	Mac UP
ENSG00000274922	1.12	0.781	2.09	0.023	Mac UP
ENSG00000274929	1.43	0.907	0.26	0.044	Mac DOWN
ENSG00000275389	1.06	0.948	12.21	0.029	Mac UP
ENSG00000276075	0.97	0.960	18.00	0.018	Mac UP
ENSG00000278730	1.28	0.073	2.19	0.036	Mac UP
ENSG00000279406	0.12	0.105	0.40	0.021	Mac DOWN
ENSG00000280160	0.14	0.041	0.24	0.109	LC DOWN
ENSG00000286153	4.02	0.228	2.13	0.030	Mac UP
ENSG00000286448	1.76	0.658	3.51	0.032	Mac UP
ENSG00000286471	3.09	0.230	2.49	0.042	Mac UP
ENSG00000287069	1.32	0.392	2.31	0.018	Mac UP
ENSG00000287300	1.13	0.903	606.00[3)]	0.033	Mac UP
ENSG00000287566			0.00	0.049	Mac DOWN

ENSG00000287733	0.79	0.705	**0.42**	**0.018**	Mac DOWN
ENSG00000288085	2.08	0.686	**0.00**	**0.049**	Mac DOWN
ENSG00000288973	1.30	0.265	**2.28**	**0.028**	Mac UP
ENSG00000289009	**0.36**	**0.021**	0.78	0.282	LC DOWN
ENSG00000289046	0.81	0.666	**0.15**	**0.047**	Mac DOWN
ENSG00000289142	0.94	0.980	**0.40**	**0.048**	Mac DOWN
ENSG00000289316	0.60	0.724	**0.18**	**0.043**	Mac DOWN
ENSG00000289507	0.70	0.773	**0.47**	**0.030**	Mac DOWN
ENSG00000289754	1.25	0.785	**3.13**	**0.036**	Mac UP
ENSG00000289915	1.64	0.640	**10.22**	**0.040**	Mac UP
ENSG00000290105	1.06	0.912	**0.44**	**0.036**	Mac DOWN
ENSG00000290383	0.80	0.759	**2.39**	**0.026**	Mac UP
ENSG00000290394	0.93	0.760	**2.43**	**0.040**	Mac UP
ENSG00000290396	1.05	0.833	**2.00**	**0.037**	Mac UP
ENSG00000290679			**0.17**	**0.042**	Mac DOWN
ENSG00000290796	1.09	0.664	**2.39**	**0.024**	Mac UP
ENSG00000290890	1.27	0.518	**2.42**	**0.037**	Mac UP
ENSG00000291044	1.53	0.217	**0.27**	**0.041**	Mac DOWN
ENSG00000291207	1.35	0.136	**2.07**	**0.026**	Mac UP
ENSG00000291270	1.17	0.656	**2.03**	**0.034**	Mac UP
ENSG00000291283			**4.28**	**0.050**	Mac UP
EPAS1	1.69	0.054	**2.48**	**0.037**	Mac UP
EPRS1	1.47	0.086	**2.19**	**0.046**	Mac UP
EPSTI1	**0.17**	**0.039**	0.12	0.050	LC DOWN
ERCC6L	0.52	0.252	**0.39**	**0.049**	Mac DOWN
ERMAP	1.28	0.235	**2.15**	**0.014**	Mac UP
ESCO2	0.47	0.157	**0.31**	**0.021**	Mac DOWN
ESPL1	0.72	0.307	**0.41**	**0.036**	Mac DOWN
ETV7	0.27	0.354	**0.49**	**0.040**	Mac DOWN
EXO1	0.82	0.561	**0.33**	**0.013**	Mac DOWN
EZH2	0.96	0.818	**0.47**	**0.035**	Mac DOWN
F5	0.33	0.342	**0.36**	**0.033**	Mac DOWN
FABP5	0.80	0.150	**0.45**	**0.031**	Mac DOWN
FADS2	0.98	0.806	**0.43**	**0.029**	Mac DOWN
FAM210B	0.92	0.516	**2.52**	**0.018**	Mac UP
FAM219B	1.01	0.871	**2.05**	**0.046**	Mac UP
FAM66B	1.79	0.629	**2.04**	**0.046**	Mac UP
FAM72B	1.20	0.625	**0.38**	**0.019**	Mac DOWN
FAM72D	1.57	0.369	**0.36**	**0.041**	Mac DOWN
FAM83A	0.35	0.088	**4.41**	**0.047**	Mac UP
FAM86JP	1.73	0.181	**3.06**	**0.048**	Mac UP
FANCI	0.55	0.100	**0.46**	**0.047**	Mac DOWN
FAP	0.76	0.593	**0.38**	**0.014**	Mac DOWN
FASN	**0.46**	**0.049**	0.50	0.075	LC DOWN
FBXO10	1.25	0.515	**2.59**	**0.030**	Mac UP
FBXO27	1.69	0.460	**2.99**	**0.025**	Mac UP
FBXO44	1.22	0.516	**2.10**	**0.030**	Mac UP
FBXO5	1.13	0.387	**0.44**	**0.015**	Mac DOWN
FBXW4	1.21	0.225	**2.10**	**0.013**	Mac UP
FCGR3A	0.32	0.438	**0.20**	**0.046**	Mac DOWN
FCRLB	1.53	0.510	**0.15**	**0.014**	Mac DOWN
FECH	1.30	0.139	**2.03**	**0.015**	Mac UP
FEN1	0.77	0.231	**0.47**	**0.014**	Mac DOWN
FGR	1.66	0.143	**2.58**	**0.011**	Mac UP
FKBP14	**2.09**	**0.009**	0.99	0.897	LC UP
FLNB-AS1	4.46	0.603	**0.00**	**0.049**	Mac DOWN
FLYWCH1	1.32	0.105	**2.03**	**0.018**	Mac UP
FOXD3			**2.96**	**0.024**	Mac UP
FOXM1	0.59	0.066	**0.42**	**0.011**	Mac DOWN
FPR3	0.00	0.629	**0.13**	**0.024**	Mac DOWN

FTCDNL1	2.71	0.108	**2.65**	**0.027**	Mac UP
FTH1	1.52	0.050	**2.17**	**0.014**	Mac UP
FXYD2	0.50	0.138	**0.11**	**0.016**	Mac DOWN
G0S2	1.02	0.682	**0.47**	**0.043**	Mac DOWN
GAB2	1.58	0.096	**2.34**	**0.020**	Mac UP
GABRG1	**5.80**	**0.050**	1.82	0.707	LC UP
GDF1	1.12	0.868	**2.14**	**0.044**	Mac UP
GGH	0.77	0.111	**0.45**	**0.037**	Mac DOWN
GINS1	0.58	0.109	**0.36**	**0.027**	Mac DOWN
GINS2	0.37	0.205	**0.38**	**0.035**	Mac DOWN
GJD3-AS1	0.96	0.686	**2.67**	**0.025**	Mac UP
GMNN	0.90	0.373	**0.40**	**0.018**	Mac DOWN
GPCPD1	1.68	0.038	**2.43**	**0.014**	Mac UP
GPR15	**0.46**	**0.048**	2.82	0.699	LC DOWN
GPR156	**2.79**	**0.021**	0.66	0.308	LC UP
GPSM2	1.27	0.167	**0.48**	**0.032**	Mac DOWN
GPT2	1.08	0.516	**2.24**	**0.034**	Mac UP
GRB10	2.37	0.401	**3.62**	**0.012**	Mac UP
GRIA2	1.73	0.662	**11.90**	**0.038**	Mac UP
GRIP2	0.37	0.263	**0.38**	**0.015**	Mac DOWN
GSDMB	0.97	0.891	**2.07**	**0.032**	Mac UP
GTF2IRD2B	1.56	0.236	**2.30**	**0.025**	Mac UP
GTPBP2	1.30	0.120	**2.07**	**0.013**	Mac UP
GTSE1	0.57	0.109	**0.37**	**0.014**	Mac DOWN
H2AC18	0.36	0.249	**0.50**	**0.018**	Mac DOWN
H2AC19	0.67	0.428	**0.49**	**0.018**	Mac DOWN
H2BC21	0.66	0.307	**0.40**	**0.024**	Mac DOWN
HAVCR2	1.71	0.338	**0.49**	**0.041**	Mac DOWN
HCG20	1.89	0.636	**3.54**	**0.038**	Mac UP
HDAC4-AS1	0.88	0.726	**0.47**	**0.033**	Mac DOWN
HELLS	0.68	0.106	**0.32**	**0.034**	Mac DOWN
HEMK1	1.50	0.088	**2.29**	**0.044**	Mac UP
HERC2P3	1.10	0.394	**2.01**	**0.033**	Mac UP
HERC5	**0.36**	**0.047**	0.32	0.117	LC DOWN
HERC6	0.38	0.059	**0.32**	**0.049**	Mac DOWN
HILPDA	0.57	0.057	**0.35**	**0.039**	Mac DOWN
HIP1	1.06	0.420	**3.63**	**0.022**	Mac UP
HJURP	0.82	0.160	**0.32**	**0.025**	Mac DOWN
HK3	0.35	0.432	**0.41**	**0.049**	Mac DOWN
HMGB3	1.03	0.661	**0.49**	**0.018**	Mac DOWN
HMMR	0.62	0.258	**0.40**	**0.014**	Mac DOWN
HPSE	1.24	0.249	**0.23**	**0.017**	Mac DOWN
HSF4	1.05	0.840	**2.21**	**0.041**	Mac UP
HSH2D	**0.12**	**0.035**	0.12	0.122	LC DOWN
HSPA1A	0.52	0.112	**0.42**	**0.042**	Mac DOWN
HSPA1B	0.50	0.127	**0.47**	**0.049**	Mac DOWN
HSPA6	**0.10**	**0.038**	**0.16**	**0.014**	Common DOWN
HTD2	0.98	0.980	**2.88**	**0.048**	Mac UP
HTR2C	0.00	0.769	**319.89**[3)]	**0.033**	Mac UP
IARS1	1.78	0.018	**2.22**	**0.012**	Mac UP
ICA1	1.50	0.102	**2.25**	**0.017**	Mac UP
ID1	0.76	0.384	**0.47**	**0.033**	Mac DOWN
IDNK	1.34	0.300	**2.02**	**0.036**	Mac UP
IER5L	**0.42**	**0.023**	0.85	0.222	LC DOWN
IFI35	**0.39**	**0.015**	**0.42**	**0.033**	Common DOWN
IFI44	**0.40**	**0.039**	0.07	0.053	LC DOWN
IFI44L	**0.30**	**0.024**	0.06	0.120	LC DOWN
IFI6	0.13	0.100	**0.16**	**0.024**	Mac DOWN
IFIH1	**0.40**	**0.044**	0.38	0.066	LC DOWN
IFIT5	**0.38**	**0.037**	**0.32**	**0.032**	Common DOWN

IFITM1	0.05	0.127	0.07	0.042	Mac DOWN
IFITM3	0.36	0.048	0.33	0.083	LC DOWN
IFRD1	1.48	0.067	2.09	0.012	Mac UP
IGSF6	0.22	0.603	0.25	0.021	Mac DOWN
IL1RN	0.48	0.147	0.41	0.012	Mac DOWN
IL7R	0.89	0.601	0.43	0.025	Mac DOWN
INAVA	0.66	0.198	0.18	0.033	Mac DOWN
INCENP	1.07	0.820	0.49	0.014	Mac DOWN
INHBE	1.94	0.395	3.12	0.014	Mac UP
IPCEF1	0.94	0.956	0.37	0.045	Mac DOWN
IQGAP3	0.60	0.260	0.42	0.016	Mac DOWN
IRF7	0.93	0.863	0.40	0.023	Mac DOWN
IRF8	0.54	0.261	0.37	0.021	Mac DOWN
IRF9	0.60	0.058	0.48	0.021	Mac DOWN
ISG15	0.16	0.044	0.16	0.042	Common DOWN
ISG20	0.27	0.016	0.25	0.139	LC DOWN
ITGA1	0.74	0.363	0.39	0.047	Mac DOWN
ITGAM	0.63	0.166	0.50	0.018	Mac DOWN
JDP2	1.68	0.100	2.09	0.014	Mac UP
KCNA2	2.88	0.217	2.22	0.036	Mac UP
KCNMB3	1.24	0.707	2.72	0.033	Mac UP
KCTD15	1.75	0.069	2.60	0.036	Mac UP
KDM5D	0.79	0.630	2.06	0.029	Mac UP
KIF15	0.58	0.332	0.45	0.022	Mac DOWN
KIF18A	0.99	0.954	0.37	0.014	Mac DOWN
KIF18B	0.67	0.285	0.32	0.015	Mac DOWN
KIF20A	0.77	0.520	0.25	0.022	Mac DOWN
KIF23	0.83	0.458	0.39	0.048	Mac DOWN
KIF27	1.74	0.123	2.31	0.016	Mac UP
KIF2C	0.75	0.106	0.30	0.022	Mac DOWN
KIF3C	0.94	0.418	0.49	0.037	Mac DOWN
KIF4A	0.91	0.672	0.36	0.015	Mac DOWN
KIFC1	0.63	0.110	0.34	0.011	Mac DOWN
KLF8	1.39	0.745	2.12	0.040	Mac UP
KNL1	0.57	0.337	0.42	0.040	Mac DOWN
LAMB3	0.62	0.189	0.39	0.014	Mac DOWN
LAP3	0.50	0.016	0.48	0.051	LC DOWN
LGALS3	1.04	0.778	2.61	0.015	Mac UP
LGALSL	1.14	0.611	2.25	0.047	Mac UP
LILRA1	2.46	0.778	0.26	0.050	Mac DOWN
LILRB2			0.39	0.014	Mac DOWN
LIMA1	1.36	0.147	0.49	0.032	Mac DOWN
LINC00174	1.73	0.178	2.34	0.047	Mac UP
LINC00638	0.80	0.696	3.05	0.027	Mac UP
LINC00662	1.45	0.303	2.36	0.043	Mac UP
LINC01127	0.63	0.739	0.29	0.015	Mac DOWN
LINC01186	0.30	0.044	0.00	0.730	LC DOWN
LINC01235	4.26	0.455	0.24	0.024	Mac DOWN
LINC01670	0.64	0.643	0.18	0.038	Mac DOWN
LINC01943	1.04	0.926	0.38	0.045	Mac DOWN
LINC02065			798.00[3)]	0.033	Mac UP
LINC02193	2.50	0.249	3.52	0.048	Mac UP
LINC02574	0.23	0.219	0.06	0.036	Mac DOWN
LINC02611	0.45	0.622	0.19	0.013	Mac DOWN
LINC02801	0.56	0.642	0.40	0.031	Mac DOWN
LINC02925	0.97	0.952	2.14	0.036	Mac UP
LINC03025_2	0.05	0.039	0.36	0.592	LC DOWN
LIPT2-AS1	1.45	0.332	2.04	0.043	Mac UP
LMNB1	0.75	0.160	0.27	0.031	Mac DOWN
LNCAROD	0.00	0.629	0.21	0.020	Mac DOWN

LPCAT3	0.26	0.011	0.60	0.016	LC DOWN
LPL	0.39	0.520	0.39	0.036	Mac DOWN
LRRC24	1.74	0.126	2.74	0.042	Mac UP
LY6E	0.47	0.189	0.37	0.014	Mac DOWN
LYZ	0.39	0.089	0.35	0.013	Mac DOWN
MAB21L2	0.42	0.035			LC DOWN
MAD2L1	0.80	0.145	0.36	0.049	Mac DOWN
MAN2A2	1.15	0.279	2.04	0.015	Mac UP
MAP1B	1.40	0.142	2.62	0.013	Mac UP
MAP7	1.00	0.993	2.47	0.032	Mac UP
MARCO	0.53	0.203	0.42	0.031	Mac DOWN
MARS1	1.42	0.022	2.24	0.016	Mac UP
MATN1-AS1	1.61	0.147	2.32	0.040	Mac UP
MCM2	0.64	0.080	0.40	0.012	Mac DOWN
MCM3	0.55	0.057	0.37	0.012	Mac DOWN
MCM4	0.61	0.043	0.40	0.012	Mac DOWN
MCM5	0.56	0.047	0.50	0.022	Mac DOWN
MCOLN3	1.10	0.754	2.81	0.026	Mac UP
MELK	0.77	0.320	0.44	0.044	Mac DOWN
Metazoa_SRP_56	1266.06[3)]	0.769	2879.04[3)]	0.033	Mac UP
MFSD4B-DT	0.00	0.644	0.00	0.049	Mac DOWN
MIA2	1.54	0.190	2.39	0.022	Mac UP
MID1IP1	0.55	0.043	0.45	0.012	Mac DOWN
MIR3142HG	2.14	0.041	1.61	0.313	LC UP
MIR4435-2HG	0.77	0.130	0.49	0.048	Mac DOWN
MKNK2	1.34	0.066	2.69	0.037	Mac UP
MMP2	1.05	0.804	0.30	0.033	Mac DOWN
MMP20	1.26	0.892	0.00	0.049	Mac DOWN
MND1	1.27	0.219	0.45	0.031	Mac DOWN
MT-ATP6	1.41	0.089	0.47	0.024	Mac DOWN
MT-ATP8	1.27	0.190	0.45	0.028	Mac DOWN
MT-CO1	0.86	0.495	0.34	0.013	Mac DOWN
MT-CO2	0.81	0.259	0.29	0.013	Mac DOWN
MT-CO3	1.03	0.772	0.40	0.014	Mac DOWN
MT-CYB	1.16	0.348	0.45	0.018	Mac DOWN
MT-ND4	1.06	0.677	0.42	0.024	Mac DOWN
MT-ND4L	1.09	0.748	0.44	0.024	Mac DOWN
MT-ND5	0.92	0.653	0.35	0.018	Mac DOWN
MT1E	1.61	0.067	5.85	0.045	Mac UP
MT1F	13.08	0.251	7.68	0.035	Mac UP
MT1G	1.54	0.395	8.54	0.045	Mac UP
MTHFD2	1.38	0.064	2.09	0.019	Mac UP
MTSS2	2.65	0.136	3.13	0.015	Mac UP
MUC12	1.30	0.825	5.62	0.030	Mac UP
MX1	0.26	0.015	0.09	0.053	LC DOWN
MXD3	1.11	0.736	0.48	0.038	Mac DOWN
MYB	0.41	0.523	0.31	0.029	Mac DOWN
MYBL2	0.41	0.063	0.33	0.013	Mac DOWN
MYBPH	0.49	0.144	0.28	0.012	Mac DOWN
MYLIP	0.20	0.101	0.14	0.031	Mac DOWN
MYO15B	1.65	0.110	2.61	0.013	Mac UP
NAMPT	1.63	0.259	2.61	0.040	Mac UP
NBL1	0.79	0.232	0.39	0.013	Mac DOWN
NBPF1	1.39	0.162	2.46	0.013	Mac UP
NBPF11	1.42	0.222	2.28	0.018	Mac UP
NBPF15	1.36	0.146	2.04	0.020	Mac UP
NCAPG	0.73	0.224	0.39	0.019	Mac DOWN
NCAPG2	0.74	0.101	0.49	0.034	Mac DOWN
NCAPH	0.62	0.203	0.44	0.014	Mac DOWN
NCKAP1	1.30	0.129	2.11	0.032	Mac UP

NDC80	0.73	0.187	**0.34**	**0.024**	Mac DOWN
NEIL3	0.70	0.506	**0.45**	**0.022**	Mac DOWN
NEK2	1.34	0.199	**0.47**	**0.015**	Mac DOWN
NET1	0.95	0.778	**0.39**	**0.027**	Mac DOWN
NEXN	0.94	0.547	**0.32**	**0.046**	Mac DOWN
NFE2L1	1.34	0.105	**2.03**	**0.033**	Mac UP
NLRC4	3.45	0.564	**2.26**	**0.033**	Mac UP
NLRP12	12.46	0.298	**5.73**	**0.045**	Mac UP
NLRP3	0.93	0.526	**0.33**	**0.037**	Mac DOWN
NMRK1	1.36	0.138	**2.03**	**0.030**	Mac UP
NOXA1	1.71	0.114	**2.92**	**0.018**	Mac UP
NPAS2	1.90	0.072	**3.17**	**0.035**	Mac UP
NPFFR2	1.27	0.085	**5.75**	**0.042**	Mac UP
NPIPB2	1.39	0.470	**2.99**	**0.036**	Mac UP
NPL	2.73	0.479	**2.52**	**0.020**	Mac UP
NR1D2	1.35	0.197	**2.03**	**0.032**	Mac UP
NR4A3	1.84	0.095	**2.23**	**0.013**	Mac UP
NSMAF	1.48	0.050	**2.65**	**0.018**	Mac UP
NTF4	**0.40**	**0.047**	0.91	0.976	LC DOWN
NUF2	0.79	0.464	**0.34**	**0.020**	Mac DOWN
NUPR1	1.67	0.543	**3.04**	**0.018**	Mac UP
NUSAP1	0.69	0.154	**0.30**	**0.020**	Mac DOWN
OAS1	**0.17**	**0.018**	0.10	0.050	LC DOWN
OAS2	**0.08**	**0.032**	0.03	0.096	LC DOWN
OASL	0.17	0.074	**0.11**	**0.016**	Mac DOWN
OLFML3	0.29	0.117	**0.24**	**0.039**	Mac DOWN
OR2A7	1.22	0.519	**3.56**	**0.020**	Mac UP
ORC1	0.47	0.084	**0.32**	**0.048**	Mac DOWN
P2RX4	1.62	0.086	**2.27**	**0.012**	Mac UP
P2RY8_2	**2.23**	**0.043**	0.64	0.629	LC UP
PACERR	3.27	0.472	**0.32**	**0.013**	Mac DOWN
PAG1	1.02	0.953	**2.03**	**0.033**	Mac UP
PAPPA2	**3.78**	**0.037**	**12.31**	**0.011**	Common UP
PAQR6	1.36	0.432	**2.55**	**0.034**	Mac UP
PARD6G-AS1	**0.43**	**0.042**	1.81	0.516	LC DOWN
PARP6	1.21	0.122	**2.33**	**0.014**	Mac UP
PARP9	**0.33**	**0.046**	**0.36**	**0.041**	Common DOWN
PBK	0.66	0.151	**0.32**	**0.048**	Mac DOWN
PCDH1	2.63	0.412	**2.18**	**0.033**	Mac UP
PCDHAC2	1.52	0.836	**3.42**	**0.040**	Mac UP
PCK2	1.41	0.085	**2.76**	**0.018**	Mac UP
PCLAF	0.67	0.179	**0.31**	**0.045**	Mac DOWN
PCNA	0.74	0.110	**0.41**	**0.018**	Mac DOWN
PDCD1LG2	0.70	0.108	**0.45**	**0.027**	Mac DOWN
PDE4B	0.93	0.723	**0.41**	**0.018**	Mac DOWN
PDE8A	1.15	0.329	**2.13**	**0.029**	Mac UP
PDK4	5.25	0.322	**3.05**	**0.031**	Mac UP
PER3	1.56	0.136	**2.76**	**0.045**	Mac UP
PGGHG	0.97	0.808	**2.07**	**0.027**	Mac UP
PHF1	1.21	0.236	**2.16**	**0.025**	Mac UP
PHGDH	1.95	0.036	**5.96**	**0.012**	Mac UP
PIK3CD	1.23	0.342	**0.45**	**0.037**	Mac DOWN
PIK3R6	0.00	0.615	**0.47**	**0.044**	Mac DOWN
PKD1P6-NPIPP1	1.07	0.875	**2.14**	**0.039**	Mac UP
PKMYT1	0.53	0.208	**0.45**	**0.013**	Mac DOWN
PLCB4	0.70	0.279	**2.08**	**0.015**	Mac UP
PLIN2	1.46	0.194	**2.13**	**0.020**	Mac UP
PLK1	0.99	0.947	**0.46**	**0.018**	Mac DOWN
PLK4	0.94	0.714	**0.40**	**0.039**	Mac DOWN
PLSCR1	**0.42**	**0.033**	0.45	0.087	LC DOWN

PNPLA1	3.73	0.567	2.52	0.035	Mac UP
PPIL6	3.22	0.154	3.08	0.011	Mac UP
PRC1	0.88	0.440	0.34	0.014	Mac DOWN
PRKACB	1.40	0.151	2.08	0.018	Mac UP
PROCR	0.71	0.057	0.35	0.045	Mac DOWN
PRSS23	0.39	0.029	0.26	0.078	LC DOWN
PRSS27	1.21	0.668	2.35	0.023	Mac UP
PRSS36	0.39	0.023	0.62	0.216	LC DOWN
PRSS8	0.30	0.023	0.62	0.590	LC DOWN
PSAT1	2.07	0.044	9.33	0.020	Common UP
PSRC1	0.88	0.692	0.48	0.043	Mac DOWN
PTER	1.17	0.671	2.99	0.012	Mac UP
PTTG1	0.90	0.278	0.34	0.024	Mac DOWN
PYCR1	2.60	0.283	2.23	0.049	Mac UP
PYGO1	1.46	0.197	2.04	0.015	Mac UP
RAB3IP	1.28	0.079	2.39	0.032	Mac UP
RACGAP1	1.02	0.791	0.48	0.016	Mac DOWN
RAD51	0.75	0.539	0.46	0.032	Mac DOWN
RAD54B	0.91	0.696	0.42	0.026	Mac DOWN
RAD54L	0.47	0.136	0.44	0.022	Mac DOWN
RASGRP3	0.85	0.818	0.36	0.017	Mac DOWN
RCC1	0.89	0.313	0.45	0.018	Mac DOWN
RELN	2.09	0.044	0.87	0.917	LC UP
RFFL	1.06	0.299	2.02	0.022	Mac UP
RFNG	1.21	0.203	2.18	0.030	Mac UP
RGS1	0.38	0.469	0.33	0.033	Mac DOWN
RGS3	0.66	0.094	0.48	0.018	Mac DOWN
RILPL1	1.08	0.451	2.00	0.027	Mac UP
RMI2	0.47	0.119	0.42	0.019	Mac DOWN
RNASE2	1358.45[3)]	0.575	0.16	0.019	Mac DOWN
RNF170	1.26	0.518	2.03	0.034	Mac UP
RNF187	1.04	0.377	2.39	0.020	Mac UP
ROPN1	0.00	0.769	685.67[3)]	0.033	Mac UP
RPS6KA2	3.10	0.134	4.30	0.020	Mac UP
RRM2	0.43	0.020	0.24	0.053	LC DOWN
RTL3	0.00	0.630	0.26	0.034	Mac DOWN
RUFY3	1.38	0.266	2.14	0.027	Mac UP
S100A10	0.60	0.045	0.45	0.018	Mac DOWN
S100A12	0.00	0.629	0.44	0.025	Mac DOWN
S100A4	0.39	0.219	0.28	0.030	Mac DOWN
SAMHD1	0.44	0.022	0.51	0.046	LC DOWN
SARS1	1.65	0.021	2.12	0.040	Mac UP
SBSN	1.74	0.047	0.28	0.016	Mac DOWN
SCD	0.25	0.051	0.38	0.016	Mac DOWN
SDSL	1.61	0.212	2.38	0.021	Mac UP
SEL1L3	1.17	0.186	2.05	0.018	Mac UP
SENP6	1.33	0.118	2.10	0.021	Mac UP
SERPINA1	1.25	0.102	0.38	0.031	Mac DOWN
SERPINB2	3.52	0.039	1.59	0.434	LC UP
SERPINB7	1.76	0.104	3.25	0.046	Mac UP
SESN2	1.90	0.070	3.25	0.026	Mac UP
SETD9	1.33	0.561	2.11	0.042	Mac UP
SGIP1	0.84	0.347	0.42	0.050	Mac DOWN
SH2D2A	0.80	0.334	0.42	0.042	Mac DOWN
SH3GLB1	1.08	0.359	2.28	0.015	Mac UP
SHCBP1	0.64	0.256	0.33	0.023	Mac DOWN
SHROOM2	0.51	0.769	0.11	0.014	Mac DOWN
SIGLEC1	0.10	0.312	0.03	0.014	Mac DOWN
SIGLEC15	0.95	0.229	0.32	0.044	Mac DOWN
SIPA1L2	1.60	0.128	2.30	0.012	Mac UP

SIRPB2	0.10	0.016	0.37	0.112	LC DOWN
SKA2	0.88	0.122	0.42	0.016	Mac DOWN
SLAMF9	0.65	0.553	0.33	0.013	Mac DOWN
SLC1A4	1.59	0.102	2.06	0.015	Mac UP
SLC1A5	1.39	0.062	2.54	0.012	Mac UP
SLC22A15	2.47	0.209	2.69	0.020	Mac UP
SLC25A19	0.92	0.551	0.44	0.022	Mac DOWN
SLC25A34	0.90	0.755	2.19	0.028	Mac UP
SLC2A10	0.59	0.768	2.77	0.032	Mac UP
SLC30A1	2.15	0.087	2.81	0.024	Mac UP
SLC38A1	1.34	0.125	2.74	0.016	Mac UP
SLC38A6	1.28	0.516	2.64	0.029	Mac UP
SLC39A10	0.42	0.071	0.27	0.026	Mac DOWN
SLC41A2	1.23	0.233	2.45	0.036	Mac UP
SLC48A1	1.19	0.133	2.16	0.041	Mac UP
SLC7A10			2.32	0.034	Mac UP
SLC7A11	3.00	0.132	4.64	0.029	Mac UP
SLC7A2	3.52	0.044	1.89	0.096	LC UP
SLFN5	0.98	0.884	2.49	0.014	Mac UP
SLIT2	2.14	0.044	0.46	0.551	LC UP
SLITRK6			6.36	0.040	Mac UP
SMARCD3	1.18	0.630	2.23	0.038	Mac UP
SMPDL3A	1.01	0.971	0.33	0.015	Mac DOWN
SNHG12	0.50	0.038	1.44	0.376	LC DOWN
SNHG7	1.23	0.197	2.36	0.022	Mac UP
SNN	1.56	0.136	2.53	0.046	Mac UP
SNRNP25	0.70	0.147	0.45	0.033	Mac DOWN
SNX20	1.04	0.962	0.48	0.028	Mac DOWN
SOBP	1.38	0.195	0.39	0.040	Mac DOWN
SP110	0.49	0.035	0.42	0.025	Common DOWN
SPAG5	0.89	0.485	0.38	0.027	Mac DOWN
SPARC	0.89	0.140	0.48	0.018	Mac DOWN
SPATA18	0.80	0.890	3.51	0.048	Mac UP
SPC24	0.33	0.116	0.33	0.021	Mac DOWN
SPC25	0.28	0.302	0.26	0.015	Mac DOWN
SPEG	1.06	0.743	2.36	0.029	Mac UP
SPIRE1	1.37	0.059	2.17	0.015	Mac UP
SPOPL-DT	1.11	0.752	6.40	0.039	Mac UP
SPP1	1.02	0.938	0.42	0.024	Mac DOWN
SQSTM1	1.60	0.045	3.30	0.013	Mac UP
SREBF1	0.33	0.021	0.34	0.048	Common DOWN
SRGN	0.85	0.490	0.48	0.021	Mac DOWN
STAR	0.56	0.734	0.21	0.049	Mac DOWN
STARD7-AS1	1.08	0.760	2.38	0.024	Mac UP
STAT1	0.44	0.047	0.90	0.664	LC DOWN
STC2	0.77	0.138	2.11	0.040	Mac UP
STK38L	0.78	0.197	0.35	0.040	Mac DOWN
STMN1	0.75	0.090	0.34	0.016	Mac DOWN
STRADB	1.22	0.403	2.05	0.029	Mac UP
STRBP	1.26	0.172	2.04	0.044	Mac UP
STRC	2.43	0.352	2.68	0.038	Mac UP
SUSD5	0.96	0.946	2.23	0.012	Mac UP
SYBU	1.96	0.256	12.59	0.033	Mac UP
SYCE2	2.24	0.048	0.40	0.215	LC UP
SYNE1	1.30	0.184	0.46	0.041	Mac DOWN
SYTL1	1.78	0.667	6.71	0.020	Mac UP
TACC3	0.65	0.171	0.47	0.023	Mac DOWN
TAGAP	1.20	0.872	0.45	0.027	Mac DOWN
TAL1	0.13	0.697	0.27	0.048	Mac DOWN
TAPT1-AS1	1.95	0.397	3.81	0.027	Mac UP

TAS2R4	1.71	0.651	**5.94**	**0.049**	Mac UP
TBL1X	1.42	0.086	**3.80**	**0.022**	Mac UP
TBX15	6.79	0.606	**2.46**	**0.030**	Mac UP
TCEA1	1.83	0.174	**2.85**	**0.031**	Mac UP
TCF19	0.50	0.073	**0.37**	**0.012**	Mac DOWN
TDRD3	1.36	0.248	**2.10**	**0.018**	Mac UP
TDRD9	2.17	0.711	**0.45**	**0.016**	Mac DOWN
TESK1	1.26	0.108	**2.10**	**0.033**	Mac UP
TFPI	0.77	0.118	**0.42**	**0.022**	Mac DOWN
TGM3	0.12	0.528	**0.35**	**0.038**	Mac DOWN
THBD	0.67	0.461	**0.49**	**0.043**	Mac DOWN
THNSL2	1.17	0.921	**2.38**	**0.015**	Mac UP
TIFAB	0.62	0.731	**0.42**	**0.048**	Mac DOWN
TK1	0.43	0.151	**0.37**	**0.014**	Mac DOWN
TLCD2	1.39	0.149	**3.29**	**0.018**	Mac UP
TLR8	2.33	0.570	**2.18**	**0.046**	Mac UP
TLX2	501.53[3)]	0.517	**1102.44[3)]**	**0.033**	Mac UP
TM4SF19-DYNLT2B	2.03	0.545	**7.14**	**0.037**	Mac UP
TMEM119	0.50	0.699	**0.42**	**0.045**	Mac DOWN
TMEM140	**0.42**	**0.039**	0.70	0.122	LC DOWN
TMEM150A	1.22	0.197	**2.63**	**0.024**	Mac UP
TMEM182	1.61	0.160	**2.08**	**0.043**	Mac UP
TMEM232	1.67	0.618	**3.25**	**0.035**	Mac UP
TMEM254-AS1	1.63	0.333	**2.66**	**0.033**	Mac UP
TMEM42	1.29	0.176	**2.12**	**0.031**	Mac UP
TMIE			**2.88**	**0.050**	Mac UP
TNIP3	2.14	0.375	**2.83**	**0.039**	Mac UP
TNNC1	0.61	0.753	**4.89**	**0.028**	Mac UP
TP73	0.52	0.089	**0.35**	**0.018**	Mac DOWN
TPX2	0.78	0.201	**0.33**	**0.013**	Mac DOWN
TRAF3IP3	1.50	0.727	**0.33**	**0.014**	Mac DOWN
TRAM2-AS1	1.18	0.570	**2.50**	**0.025**	Mac UP
TREM1	0.17	0.291	**0.18**	**0.013**	Mac DOWN
TRIB2	1.28	0.338	**0.48**	**0.014**	Mac DOWN
TRIM14	**0.38**	**0.035**	0.58	0.017	LC DOWN
TRIM16L	1.09	0.684	**3.14**	**0.014**	Mac UP
TRIM34	0.50	0.156	**0.41**	**0.036**	Mac DOWN
TRIP13	0.94	0.514	**0.45**	**0.030**	Mac DOWN
TROAP	0.79	0.330	**0.44**	**0.022**	Mac DOWN
TSEN15	1.31	0.120	**2.09**	**0.020**	Mac UP
TSTD3	1.01	0.949	**2.04**	**0.016**	Mac UP
TTK	0.94	0.721	**0.38**	**0.032**	Mac DOWN
TTLL4	1.29	0.089	**2.18**	**0.018**	Mac UP
TUBA1A	0.70	0.094	**0.39**	**0.018**	Mac DOWN
TUBA1B	0.58	0.100	**0.47**	**0.015**	Mac DOWN
TUBB3	0.60	0.048	**0.41**	**0.018**	Mac DOWN
TUBE1	1.52	0.180	**2.51**	**0.018**	Mac UP
TYMS	0.53	0.023	**0.28**	**0.014**	Mac DOWN
UBE2C	1.01	0.977	**0.40**	**0.017**	Mac DOWN
UBE2T	0.80	0.247	**0.41**	**0.032**	Mac DOWN
UCN2	0.72	0.158	**0.31**	**0.037**	Mac DOWN
UHRF1	0.52	0.101	**0.25**	**0.015**	Mac DOWN
ULBP1	2.68	0.147	**3.36**	**0.015**	Mac UP
UNC13A	0.89	0.601	**2.26**	**0.024**	Mac UP
UNC5B	6.43	0.129	**5.24**	**0.025**	Mac UP
UST-AS2	1.04	0.959	**0.31**	**0.038**	Mac DOWN
UVSSA	1.53	0.113	**2.37**	**0.030**	Mac UP
VCAM1	**0.33**	**0.040**	0.35	0.160	LC DOWN
VPS13C	1.35	0.230	**2.24**	**0.030**	Mac UP
VTN	1.06	0.973	**0.32**	**0.021**	Mac DOWN

VWA7	0.69	0.498	**4.24**	**0.043**	Mac UP
WARS1	1.53	0.175	**2.27**	**0.044**	Mac UP
WARS2-AS1	1.24	0.044	**2.31**	**0.011**	Mac UP
WDR31	1.44	0.042	**2.08**	**0.049**	Mac UP
WDR76	0.84	0.148	**0.36**	**0.024**	Mac DOWN
WIPI1	1.72	0.071	**2.02**	**0.031**	Mac UP
XAF1	**0.21**	**0.034**	**0.41**	**0.034**	Common DOWN
XPOT	1.70	0.054	**3.05**	**0.024**	Mac UP
ZBED3	1.31	0.591	**3.18**	**0.024**	Mac UP
ZDHHC1	1.45	0.737	**2.28**	**0.024**	Mac UP
ZFAND3	1.22	0.134	**2.16**	**0.037**	Mac UP
ZFP41	1.34	0.253	**2.22**	**0.046**	Mac UP
ZNF181	1.82	0.181	**2.35**	**0.020**	Mac UP
ZNF22-AS1	1.15	0.962	**2.49**	**0.033**	Mac UP
ZNF252P	1.34	0.147	**2.17**	**0.015**	Mac UP
ZNF367	0.61	0.322	**0.38**	**0.019**	Mac DOWN
ZNF385C	2.90	0.087	**5.93**	**0.020**	Mac UP
ZNF425	1.66	0.445	**2.34**	**0.028**	Mac UP
ZNF853	1.82	0.485	**2.23**	**0.014**	Mac UP
ZWINT	0.41	0.151	**0.40**	**0.012**	Mac DOWN

The numbers in blue, red and purple indicate |log2FC| > 1 and Padj < 0.05 in HLF cells, macrophages and common, respectively, where FC and Padj mean fold change and adjusted P value, respectively.

1) Fold changes were calculated as the ratio of mean expression levels (TPM) in HX531 treatment group to those in control group.

2) Blank indicates no expression.

3) Zero TPM in control group was replaced with 0.0001, and then fold changes were calculated.

Supplementary Table 5. Pathways predicted by GoTerm Biological Process

Pathways enriched from DEGs in HLF cells

Term	Count	Fold Enric	FDR	Genes
GO:0051607~defense response to virus	15	22.9	0.000	IFITM3, STAT1, MX1, IFIT5, ISG15, PARP9, SAMHD1, IFI44L, IFIH1, ISG20, HERC5, PLSCR1, OAS1, OAS2, BCL2L1
GO:0045071~negative regulation of viral genome replication	9	72.0	0.000	ISG20, IFIH1, IFITM3, PLSCR1, OAS1, OAS2, MX1, IFIT5, ISG15
GO:0009615~response to virus	9	29.5	0.000	ISG20, IFIH1, IFITM3, OAS1, OAS2, MX1, IFI44, ISG15, BCL2L1
GO:0070106~interleukin-27-mediated signaling pathway	4	205.9	0.000	OAS1, STAT1, OAS2, MX1
GO:0045087~innate immune response	12	6.8	0.000	ISG20, IFIH1, HERC5, OAS1, OAS2, MX1, IFIT5, TRIM14, ISG15, IFI35, PARP9, SAMHD1
GO:0035456~response to interferon-beta	4	131.0	0.000	IFITM3, PLSCR1, STAT1, XAF1
GO:0060337~type I interferon signaling pathway	5	32.2	0.001	IFIH1, IFITM3, OAS1, STAT1, OAS2
GO:0032728~positive regulation of interferon-beta production	4	33.5	0.015	IFIH1, OAS1, OAS2, ISG15
GO:0060339~negative regulation of type I interferon-mediated signaling pathway	3	49.1	0.099	OAS1, ISG15, SAMHD1
GO:0034341~response to interferon-gamma	3	40.0	0.135	IFITM3, CD74, STAT1
GO:0006508~proteolysis	6	4.7	0.402	RELN, LAP3, PRSS36, PRSS8, PRSS23, PAPPA2
GO:0060700~regulation of ribonuclease activity	2	144.1	0.622	OAS1, OAS2
GO:0032020~ISG15-protein conjugation	2	120.1	0.688	HERC5, ISG15
GO:0034340~response to type I interferon	2	72.0	0.993	MX1, ISG15
GO:0098586~cellular response to virus	3	11.4	0.993	IFIH1, CXCL10, OAS1
GO:0032760~positive regulation of tumor necrosis factor production	3	10.0	0.993	IFIH1, OAS1, OAS2
GO:0010818~T cell chemotaxis	2	48.0	0.993	CXCL10, CXCL11
GO:0032897~negative regulation of viral transcription	2	45.0	0.993	IFITM3, TRIM14
GO:0060384~innervation	2	42.4	0.993	NTF4, VCAM1
GO:0006955~immune response	5	3.5	0.993	IFITM3, CD74, IFI44, SAMHD1, IFI44L
GO:0051259~protein oligomerization	2	34.3	0.993	IFIH1, OAS1
GO:0071398~cellular response to fatty acid	2	31.3	0.993	SREBF1, E2F1
GO:0046597~negative regulation of viral entry into host cell	2	30.0	0.993	IFITM3, CD74
GO:0032727~positive regulation of interferon-alpha production	2	27.7	0.993	IFIH1, STAT1
GO:0035458~cellular response to interferon-beta	2	25.7	0.993	OAS1, STAT1
GO:0007214~gamma-aminobutyric acid signaling pathway	2	24.8	0.993	GPR156, GABRG1
GO:0051281~positive regulation of release of sequestered calcium ion into cytosol	2	23.2	0.993	CXCL10, CXCL11
GO:0042127~regulation of cell proliferation	3	6.2	0.993	CXCL10, CXCL11, STAT1
GO:0045664~regulation of neuron differentiation	2	22.5	0.993	NTF4, RELN
GO:0045089~positive regulation of innate immune response	2	22.5	0.993	PLSCR1, IFI35
GO:0008283~cell proliferation	3	6.1	0.993	CD74, MAB21L2, BCL2L1
GO:0008015~blood circulation	2	21.2	0.993	CXCL10, STAT1
GO:0006401~RNA catabolic process	2	21.2	0.993	ISG20, OAS2
GO:0051085~chaperone mediated protein folding requiring cofactor	2	21.2	0.993	CD74, HSPA6
GO:0002230~positive regulation of defense response to virus by host	2	20.6	0.993	STAT1, PARP9
GO:0045648~positive regulation of erythrocyte differentiation	2	20.6	0.993	STAT1, ISG15
GO:0000077~DNA damage checkpoint	2	19.5	0.993	E2F1, PARP9
GO:0007616~long-term memory	2	19.0	0.993	NTF4, RELN

Pathways enriched from DEGs in macrophages

Term	Count	Fold Enric	FDR	Genes

GO:0051301~cell division	51	5.3	0.000	GPSM2, ERCC6L, CCNF, NCAPG2, BUB1B, CDC20, TUBA1B, TUBA1A, PTTG1, NUF2, RCC1, NEK2, FBXO5, HELLS, KNL1, CDC25C, CDC25A, CCNA2, PSRC1, CCNE2, KIFC1, CKS2, BIRC5, MCM5, KIF2C, CDCA3, CDCA5, NCAPG, CDCA8, NCAPH, AURKB, AURKA, SKA2, CCNB2, CCNB1, CDT1, CENPW, SPAG5, UBE2C, CDC7, NDC80, ZWINT, CENPE, TPX2, KIF18B, PRC1, CDK1, TACC3, SPC24, SPC25, MAD2L1
GO:0007052~mitotic spindle organization	18	12.3	0.000	GPSM2, PLK1, CDCA8, TTK, NDC80, AURKB, AURKA, CENPE, CCNB1, INCENP, KIF4A, NUF2, STMN1, TACC3, BIRC5, RCC1, DLGAP5, SPC25
GO:0000070~mitotic sister chromatid segregation	15	15.7	0.000	SPAG5, PLK1, NCAPG2, CDCA8, KNL1, NDC80, ZWINT, KIF18A, KIF18B, ESPL1, INCENP, KIFC1, NUSAP1, NEK2, MAD2L1
GO:0000278~mitotic cell cycle	25	6.5	0.000	CDCA5, CDCA8, PKMYT1, AURKB, AURKA, SKA2, KIF15, TUBA1B, TUBA1A, TUBB3, MYB, PBK, NEK2, MYBL2, PLK4, CDT1, CENPW, PLK1, TUBE1, NDC80, CENPE, TPX2, KIF18B, INCENP, BIRC5
GO:0007094~mitotic spindle assembly checkpoint	13	16.2	0.000	PLK1, BUB1B, TTK, NDC80, ZWINT, AURKB, CDC20, NUF2, BIRC5, TRIP13, SPC24, MAD2L1, SPC25
GO:0007059~chromosome segregation	18	7.4	0.000	CDT1, CENPW, SPAG5, HJURP, TTK, ESCO2, NDC80, SKA2, CENPE, INCENP, NUF2, BIRC5, RCC1, NEK2, CENPQ, SPC24, DLGAP5, SPC25
GO:0051256~mitotic spindle midzone assembly	8	28.9	0.000	RACGAP1, INCENP, PRC1, KIF4A, CDCA8, BIRC5, KIF23, AURKB
GO:0000281~mitotic cytokinesis	14	8.2	0.000	PLK1, CDCA8, KIF23, CENPA, AURKB, ANLN, ESPL1, RACGAP1, INCENP, KIF4A, STMN1, NUSAP1, BIRC5, KIF20A
GO:0006268~DNA unwinding involved in DNA replication	9	16.3	0.000	GINS1, GINS2, BLM, RAD51, CDC45, MCM3, MCM4, MCM5, MCM2
GO:0000727~double-strand break repair via break-induced replication	7	23.2	0.000	GINS2, CDC45, MCM3, MCM4, CDC7, MCM5, MCM2
GO:0000079~regulation of cyclin-dependent protein serine/threonine kinase activity	11	8.1	0.000	CDKN2D, CCNA2, CCNB2, BLM, CCNB1, CCNE2, CCNF, PKMYT1, CDC25C, CDC25A, CDKN3
GO:0000086~G2/M transition of mitotic cell cycle	11	7.5	0.000	CCNA2, CCNB1, MELK, PLK1, CDK1, BIRC5, PKMYT1, FOXM1, CDC25C, CDC25A, AURKA
GO:0007049~cell cycle	27	2.9	0.000	ERCC6L, CDCA3, UHRF1, CDCA5, HJURP, CDCA8, AURKA, CDC45, NUF2, E2F2, CDKN2D, FANCI, CDT1, HELLS, STRADB, PLK1, CDC25C, CDC25A, CCPG1, MCM3, BIRC5, MCM4, MCM5, SPC24, CDKN3, MCM2, TP73
GO:0010971~positive regulation of G2/M transition of mitotic cell cycle	8	11.0	0.001	CCNB1, SMARCD3, CDK1, CDC7, FBXO5, CDC25C, DTL, CDC25A
GO:0090267~positive regulation of mitotic cell cycle spindle assembly checkpoint	6	19.9	0.001	INCENP, CDCA8, BIRC5, NDC80, AURKB, MAD2L1
GO:0007018~microtubule-based movement	12	5.4	0.002	DNAI4, CENPE, KIF18A, KIF18B, KIFC1, KIF4A, KIF23, KIF2C, KIF20A, KIF3C, KIF27, KIF15
GO:0030174~regulation of DNA-dependent DNA replication initiation	6	17.0	0.002	CDT1, GMNN, MCM3, MCM4, MCM5, MCM2
GO:0006270~DNA replication initiation	7	9.9	0.007	CDC45, ORC1, CCNE2, MCM3, MCM4, MCM5, MCM2
GO:0006260~DNA replication	13	4.2	0.007	GINS2, BLM, FEN1, RMI2, PCNA, DSCC1, ORC1, PCLAF, CDK1, MCM4, MCM5, DTL, MCM2
GO:0007057~spindle assembly involved in female meiosis I	4	39.8	0.007	CCNB2, FBXO5, NDC80, AURKA
GO:1901970~positive regulation of mitotic sister chromatid separation	4	39.8	0.007	INCENP, CDCA8, BIRC5, AURKB
GO:0008608~attachment of spindle microtubules to kinetochore	6	13.3	0.007	NUF2, KNL1, NDC80, SPC24, AURKB, SPC25
GO:0007080~mitotic metaphase plate congression	8	7.6	0.008	CENPE, KIF18A, CCNB1, PSRC1, KIFC1, CDCA5, CDCA8, KIF2C
GO:0051315~attachment of mitotic spindle microtubules to kinetochore	5	16.6	0.016	CDT1, CENPE, NUF2, KIF2C, NDC80
GO:1903490~positive regulation of mitotic cytokinesis	4	26.5	0.028	INCENP, CDCA8, BIRC5, AURKB
GO:0051310~metaphase plate congression	5	14.2	0.028	CENPE, INCENP, KIF2C, CENPQ, NDC80
GO:0000082~G1/S transition of mitotic cell cycle	9	5.2	0.028	CCNA2, CCNE2, CDK1, RCC1, CDC7, IQGAP3, CDC25A, EZH2, CDKN3

GO:0044772~mitotic cell cycle phase transition	6	9.5	0.029	CCNA2, CCNB2, CCNB1, CCNE2, CCNF, CKS2
GO:0042773~ATP synthesis coupled electron transport	4	22.7	0.042	MT-ND4L, MT-ND4, MT-ND5, MT-CO2
GO:0032465~regulation of cytokinesis	7	6.6	0.046	SH3GLB1, PLK4, PRC1, PLK1, KIF20A, AURKB, AURKA
GO:1902425~positive regulation of attachment of mitotic spindle microtubules to kinetochore	4	19.9	0.061	INCENP, CDCA8, BIRC5, AURKB
GO:0007076~mitotic chromosome condensation	5	10.5	0.081	CDCA5, PLK1, NUSAP1, NCAPG, NCAPH
GO:0030593~neutrophil chemotaxis	9	4.3	0.081	LGALS3, CCL7, CCL20, ITGA1, CCL4, PDE4B, S100A12, PIK3CD, TREM1
GO:0006868~glutamine transport	4	17.7	0.082	SLC38A1, SLC38A6, SLC1A4, SLC1A5
GO:0001578~microtubule bundle formation	6	7.2	0.087	PSRC1, PRC1, MAP1B, PLK1, CCSER2, KIF20A
GO:0007346~regulation of mitotic cell cycle	8	4.7	0.093	CDC20, PLK1, GMNN, CKS2, BIRC5, FBXO5, DLGAP5, TP73
GO:0044267~cellular protein metabolic process	5	9.5	0.107	FBXO27, MMP2, D2HGDH, FBXO44, PAPPA2
GO:0007088~regulation of mitotic nuclear division	5	8.6	0.148	RCC1, NEK2, FBXO5, PKMYT1, CDC25C
GO:0030218~erythrocyte differentiation	7	5.1	0.150	RACGAP1, FECH, EPAS1, TAL1, MYB, NCAPG2, SLC1A5
GO:0006418~tRNA aminoacylation for protein translation	5	8.3	0.165	AARS1, EPRS1, MARS1, IARS1, WARS1
GO:0034501~protein localization to kinetochore	4	13.3	0.168	CDK1, TTK, KNL1, AURKB
GO:1905342~positive regulation of protein localization to kinetochore	3	29.8	0.191	CDT1, CENPQ, NDC80
GO:0015990~electron transport coupled proton transport	3	29.8	0.191	MT-ND4, MT-ND5, MT-CO1
GO:0000226~microtubule cytoskeleton organization	11	3.0	0.191	TUBA1B, TTLL4, TUBA1A, TUBB3, PRC1, MAP1B, CDK1, TACC3, MAP7, BIRC5, TUBE1
GO:0051444~negative regulation of ubiquitin-protein transferase activity	4	12.2	0.191	CDC20, BUB1B, FBXO5, MAD2L1
GO:1900264~positive regulation of DNA-directed DNA polymerase activity	4	12.2	0.191	GINS1, GINS2, PCNA, DSCC1
GO:0009636~response to toxic substance	8	3.9	0.212	SNN, RAD51, CCL4, CDK1, NUPR1, SLC7A11, TYMS, ATF4
GO:0045842~positive regulation of mitotic metaphase/anaphase transition	4	11.4	0.229	CDC20, ESPL1, UBE2C, DLGAP5
GO:0007165~signal transduction	49	1.5	0.250	ARHGAP11A, IL1RN, ECM1, PIK3CD, RGS3, RGS1, ADORA3, RPS6KA2, NAMPT, TAGAP, STMN1, CAPN5, PDE4B, GRB10, PDE8A, PRKACB, ARAP2, GAB2, THNSL2, NBL1, NSMAF, GRIA2, CHRNA5, EPAS1, SH2D2A, ARHGAP18, FPR3, AKAP5, IQGAP3, RACGAP1, CCL7, OLFML3, CCL4, SPP1, NLRP3, APBB1, PAG1, FAM83A, CCL20, GDF1, CDC7, LILRB2, INHBE, NET1, NLRP12, P2RX4, NR4A3, IL7R, HSPA1A
GO:1902975~mitotic DNA replication initiation	3	23.9	0.283	MCM3, MCM4, MCM2
GO:0006275~regulation of DNA replication	6	5.1	0.291	CCNA2, PCNA, DSCC1, GMNN, ESCO2, FBXO5
GO:0009615~response to virus	9	3.3	0.296	FGR, BST2, IFITM1, STMN1, CCL4, IRF7, TLR8, ISG15, OASL
GO:0032731~positive regulation of interleukin-1 beta production	7	4.2	0.296	NLRP12, NLRP3, LPL, TLR8, NLRC4, CD36, INAVA
GO:0007019~microtubule depolymerization	4	9.9	0.296	KIF18A, KIF18B, STMN1, KIF2C
GO:0051382~kinetochore assembly	4	9.9	0.296	CENPE, CENPW, CENPA, DLGAP5
GO:0090102~cochlea development	5	6.4	0.304	CCNA2, TIFAB, SLITRK6, SOBP, MCM2
GO:0031536~positive regulation of exit from mitosis	3	19.9	0.366	UBE2C, CDCA5, BIRC5
GO:0007131~reciprocal meiotic recombination	5	6.0	0.367	RAD51, RAD54L, MND1, TRIP13, RAD54B
GO:0019538~protein metabolic process	4	8.8	0.388	HEMK1, MMP2, D2HGDH, PAPPA2
GO:0045931~positive regulation of mitotic cell cycle	5	5.9	0.394	CCNB1, TAL1, ASNS, BIRC5, AURKA
GO:0045648~positive regulation of erythrocyte differentiation	5	5.7	0.423	FAM210B, TAL1, ISG15, HSPA1B, HSPA1A
GO:0007051~spindle organization	4	8.4	0.423	SPAG5, TTK, AURKB, AURKA
GO:0042692~muscle cell differentiation	4	8.4	0.423	SMARCD3, IFRD1, SPEG, SYNE1
GO:0051383~kinetochore organization	3	17.0	0.431	CDT1, NUF2, NDC80
GO:0000132~establishment of mitotic spindle orientation	5	5.5	0.431	GPSM2, PLK1, CENPA, NDC80, MAD2L1
GO:0006882~cellular zinc ion homeostasis	5	5.5	0.431	MT1F, SLC39A10, MT1G, SLC30A1, MT1E
GO:0097421~liver regeneration	5	5.5	0.431	VTN, PCNA, TYMS, EZH2, AURKA

GO:2001240~negative regulation of extrinsic apoptotic signaling pathway in absence of ligand	5	5.4	0.467	UNC5B, STRADB, IFI6, HSPA1B, HSPA1A
GO:0006915~apoptotic process	26	1.7	0.471	NCKAP1, HIP1, IFI6, BUB1B, NLRC4, AURKA, NLRP3, APBB1, CD14, RFFL, SH3GLB1, SRGN, HELLS, UNC5B, FBXO10, TPX2, NLRP12, MELK, ESPL1, CDK1, BIRC5, BIRC7, XAF1, SQSTM1, MCM2, TP73
GO:0032729~positive regulation of interferon-gamma production	7	3.5	0.483	CEBPG, PDE4B, IRF8, TLR8, ISG15, CD14, HAVCR2
GO:0007017~microtubule-based process	5	5.2	0.483	TUBA1B, TUBA1A, TUBB3, TUBE1, GTSE1
GO:0006954~inflammatory response	20	1.8	0.483	IL1RN, ECM1, CCL20, PIK3CD, FPR3, NLRC4, LYZ, CCL7, ADORA3, TNIP3, CCL4, SPP1, S100A12, TLR8, NLRP3, CD14, HPSE, SIGLEC1, HAVCR2, CMKLR1
GO:0060236~regulation of mitotic spindle organization	4	7.6	0.483	GPSM2, TPX2, PSRC1, TACC3
GO:0002244~hematopoietic progenitor cell differentiation	7	3.5	0.487	DNAI4, PYGO1, ANLN, TIFAB, ESCO2, BVES, HERC6
GO:0015825~L-serine transport	3	14.9	0.494	SLC1A4, SLC1A5, SLC7A10
GO:0045143~homologous chromosome segregation	3	14.9	0.494	PTTG1, ESPL1, PLK1
GO:0007095~mitotic G2 DNA damage checkpoint	5	5.1	0.494	BLM, PLK1, CDK1, CLSPN, DTL
GO:0051607~defense response to virus	13	2.2	0.506	IFITM1, IFI6, IFIT5, ISG15, PARP9, RNASE2, OASL, BST2, IRF7, TLR8, NLRP3, IRF9, TRIM34
GO:1900182~positive regulation of protein localization to nucleus	5	5.0	0.525	SESN2, PLK1, CDK1, TESK1, PARP9
GO:0018105~peptidyl-serine phosphorylation	11	2.4	0.532	RPS6KA2, PLK1, MKNK2, PBK, CDK1, STK38L, TTK, CDC7, CLSPN, PRKACB, AURKA
GO:0034644~cellular response to UV	6	3.9	0.535	PCNA, PBK, CDC25A, AURKB, ATF4, AQP1
GO:0090307~mitotic spindle assembly	5	4.9	0.545	CDC20, TPX2, KIFC1, MYBL2, NEK2
GO:0006974~cellular response to DNA damage stimulus	15	2.0	0.545	BLM, UHRF1, PHF1, WDR76, NPAS2, RAD51, UBE2T, PCLAF, IRF7, FLYWCH1, APBB1, FBXO5, SPATA18, DTL, TP73
GO:0007166~cell surface receptor signaling pathway	16	1.9	0.552	CD86, IFITM1, IGSF6, LILRB2, LILRA1, PDCD1LG2, FCRLB, MARCO, FCGR3A, MKNK2, CD14, CD36, IL7R, IRF9, PAPPA2, LY6E
GO:0007091~metaphase/anaphase transition of mitotic cell cycle	3	13.3	0.552	PLK1, TACC3, BUB1B
GO:1905821~positive regulation of chromosome condensation	3	13.3	0.552	NCAPG2, NCAPG, NCAPH
GO:0006865~amino acid transport	5	4.7	0.552	SLC38A1, SLC1A4, SLC7A11, SLC1A5, SLC7A10
GO:0045333~cellular respiration	5	4.7	0.552	NR4A3, MT-CO1, MT-CO2, MT-CO3, MT-CYB
GO:0008203~cholesterol metabolic process	7	3.2	0.552	CYP27A1, SREBF1, LIMA1, STAR, FECH, APOC1, NFE2L1
GO:0071294~cellular response to zinc ion	4	6.6	0.552	P2RX4, MT1F, MT1G, MT1E
GO:1901673~regulation of mitotic spindle assembly	4	6.6	0.552	PLK1, HSPA1B, KIF15, HSPA1A
GO:0048260~positive regulation of receptor-mediated endocytosis	4	6.6	0.552	VTN, HIP1, SGIP1, SYNE1
GO:0034080~CENP-A containing nucleosome assembly	3	11.9	0.629	CENPW, HJURP, CENPA
GO:0045071~negative regulation of viral genome replication	5	4.4	0.646	BST2, IFITM1, IFIT5, ISG15, OASL
GO:0002548~monocyte chemotaxis	5	4.4	0.646	LGALS3, CCL7, CCL20, CCL4, S100A12
GO:0031214~biomineral tissue development	4	5.9	0.683	SRGN, ECM1, TMEM119, SPP1
GO:0071280~cellular response to copper ion	4	5.9	0.683	MT1F, MT1G, AQP1, MT1E
GO:0070301~cellular response to hydrogen peroxide	6	3.5	0.683	NET1, PCNA, MYB, CDK1, EZH2, AQP1
GO:1905820~positive regulation of chromosome separation	3	10.8	0.683	NCAPG2, NCAPG, NCAPH
GO:0035456~response to interferon-beta	3	10.8	0.683	BST2, IFITM1, XAF1
GO:1904668~positive regulation of ubiquitin protein ligase activity	3	10.8	0.683	CDC20, UBE2C, PLK1
GO:0051988~regulation of attachment of spindle microtubules to kinetochore	3	10.8	0.683	SPAG5, RACGAP1, NEK2
GO:0000076~DNA replication checkpoint	3	10.8	0.683	CDT1, CDC45, CLSPN

GO:0045087~innate immune response	25	1.6	0.699	ITGAM, IFIT5, IFI6, HMGB3, PIK3CD, IFI35, NLRC4, TREM1, INAVA, OASL, LGALS3, S100A12, NLRP3, CD14, HAVCR2, ISG15, PARP9, FGR, BST2, MARCO, IRF7, SPIRE1, TLR8, TRIM34, RNF187
GO:0007098~centrosome cycle	5	4.1	0.713	TUBA1A, PLK1, CDK1, PCLAF, TUBE1
GO:0097009~energy homeostasis	5	4.1	0.713	NR4A3, SGIP1, NR1D2, CD36, SQSTM1
GO:0045740~positive regulation of DNA replication	4	5.7	0.714	CDT1, PCNA, BRPF3, CDK1
GO:0016310~phosphorylation	14	1.9	0.731	TESK1, PIK3CD, BUB1B, TTK, CDC7, PKMYT1, NMRK1, RPS6KA2, PBK, PDK4, SPEG, IDNK, TK1, ALPK2
GO:0006955~immune response	21	1.6	0.731	CD86, IL1RN, IGSF6, CCL20, CEBPG, IFI6, PIK3CD, LILRB2, PDCD1LG2, PIK3R6, VTN, FCGR3A, RGS1, FTH1, CCL4, IRF8, ACKR3, CD36, IL7R, ULBP1, CMKLR1
GO:0051056~regulation of small GTPase mediated signal transduction	8	2.6	0.735	ARHGAP11A, NET1, RACGAP1, TAGAP, ARAP2, ARHGAP18, SIPA1L2, ARHGEF5
GO:1901224~positive regulation of NIK/NF-kappaB signaling	6	3.3	0.736	CD86, NLRP12, NLRP3, CD14, IFI35, HAVCR2
GO:0034599~cellular response to oxidative stress	7	2.9	0.745	SESN2, PYCR1, SLC7A11, HSPA1B, NFE2L1, ATF4, HSPA1A
GO:0051726~regulation of cell cycle	13	1.9	0.827	PLK1, CCNF, FOXM1, CCNE2, FAP, PCLAF, BIRC5, E2F2, BIRC7, DTL, PRKACB, CDKN3, TP73
GO:0071248~cellular response to metal ion	3	9.2	0.846	MT1F, MT1G, MT1E
GO:0003333~amino acid transmembrane transport	4	5.0	0.915	SLC38A1, SLC38A6, SLC7A11, SLC7A10
GO:0051276~chromosome organization	4	5.0	0.915	CENPW, PTTG1, RAD54L, CDCA8
GO:0051984~positive regulation of chromosome segregation	3	8.5	0.915	NCAPG2, NCAPG, NCAPH
GO:0019985~translesion synthesis	3	8.5	0.915	PCNA, PCLAF, DTL
GO:0030195~negative regulation of blood coagulation	3	8.5	0.915	THBD, VTN, TFPI
GO:0016567~protein ubiquitination	20	1.6	0.915	MYLIP, DET1, CDCA3, UHRF1, UBE2C, FBXO27, HERC2P3, PLK1, CCNF, FBXO10, FBXO44, CDC20, UVSSA, RNF170, DCAF10, BIRC7, FBXO5, HERC6, RNF187, TRIM34
GO:0009410~response to xenobiotic stimulus	13	1.9	0.915	SREBF1, FECH, MMP2, LPL, TYMS, NPAS2, RAD54B, RAD51, MAP1B, CDK1, RAD54L, PLIN2, TP73
GO:0031334~positive regulation of protein complex assembly	5	3.6	0.915	SH3GLB1, CDT1, LGALS3, TAL1, WARS1
GO:2000104~negative regulation of DNA-dependent DNA replication	2	39.8	0.915	CDT1, GMNN
GO:2000521~negative regulation of immunological synapse formation	2	39.8	0.915	LGALS3, HAVCR2
GO:0000022~mitotic spindle elongation	2	39.8	0.915	PRC1, KIF23
GO:2000866~positive regulation of estradiol secretion	2	39.8	0.915	SPP1, CYP19A1
GO:0071163~DNA replication preinitiation complex assembly	2	39.8	0.915	CDT1, GMNN
GO:0008315~meiotic G2/MI transition	2	39.8	0.915	CCNB2, NDC80
GO:0043547~positive regulation of GTPase activity	10	2.1	0.941	ARHGAP11A, NET1, CCL7, CCL20, RGS1, CCL4, ARAP2, EZH2, RASGRP3, S100A10
GO:0001932~regulation of protein phosphorylation	5	3.6	0.941	RAD51, SESN2, TLR8, ROPN1, EZH2
GO:0002221~pattern recognition receptor signaling pathway	3	8.0	0.952	NLRP3, NLRC4, INAVA
GO:0006636~unsaturated fatty acid biosynthetic process	3	8.0	0.952	FADS2, SCD, ELOVL6
GO:0032436~positive regulation of proteasomal ubiquitin-dependent protein catabolic process	6	2.9	0.987	DET1, PLK1, TRIB2, HSPA1B, AURKA, HSPA1A
GO:0001525~angiogenesis	12	1.9	0.987	ECM1, FAP, UNC5B, EPAS1, TAL1, ADM2, SH2D2A, MMP2, ID1, ACKR3, PIK3R6, WARS1
GO:0015909~long-chain fatty acid transport	3	7.5	0.987	FABP5, PLIN2, CD36
GO:0010273~detoxification of copper ion	3	7.5	0.987	MT1F, MT1G, MT1E
GO:0035563~positive regulation of chromatin binding	3	7.5	0.987	CDT1, GMNN, PARP9
GO:0030071~regulation of mitotic metaphase/anaphase transition	4	4.4	0.987	CENPE, SMARCD3, UBE2C, PLK1
GO:0071222~cellular response to lipopolysaccharide	10	2.0	0.987	CD86, TNIP3, PDE4B, IRF8, NLRP3, LILRB2, CD14, CD36, PDCD1LG2, HAVCR2

GO:0071383~cellular response to steroid hormone stimulus	3	7.0	0.987	TFPI, HSPA1B, HSPA1A
GO:0043525~positive regulation of neuron apoptotic process	5	3.2	0.987	ITGA1, MYB, MYBL2, NUPR1, ATF4
GO:0042149~cellular response to glucose starvation	5	3.2	0.987	SH3GLB1, SESN2, ASNS, PLIN2, ATF4
GO:2001189~negative regulation of T cell activation via T cell receptor contact with antigen bound to I	2	26.5	0.987	LGALS3, HAVCR2
GO:0034421~post-translational protein acetylation	2	26.5	0.987	DSCC1, ESCO2
GO:1902850~microtubule cytoskeleton organization involved in mitosis	2	26.5	0.987	CDK1, TACC3
GO:0009056~catabolic process	2	26.5	0.987	TMEM150A, PTER
GO:0032461~positive regulation of protein oligomerization	2	26.5	0.987	ISG15, ZDHHC1
GO:0034371~chylomicron remodeling	2	26.5	0.987	APOC2, LPL
GO:0055096~low-density lipoprotein particle mediated signaling	2	26.5	0.987	LPL, CD36
GO:0045926~negative regulation of growth	3	6.6	0.987	MT1F, MT1G, MT1E
GO:0071392~cellular response to estradiol stimulus	4	4.0	0.987	FAM210B, CCNA2, KIF18A, MMP2
GO:0006281~DNA repair	13	1.7	0.987	FANCI, BLM, FEN1, RMI2, UHRF1, FOXM1, RAD51, PTTG1, EXO1, UBE2T, CDK1, RAD54L, CLSPN
GO:0008283~cell proliferation	9	2.0	0.987	MELK, SH2D2A, CDK1, CKS2, TACC3, CDC25C, CDC25A, AURKB, FAM83A
GO:0042776~mitochondrial ATP synthesis coupled proton transport	5	3.1	0.987	MT-ATP6, MT-ND4L, MT-ND4, MT-ND5, MT-ATP8
GO:0042326~negative regulation of phosphorylation	3	6.3	0.987	CDKN2D, GRB10, TESK1
GO:0033280~response to vitamin D	3	6.3	0.987	CDKN2D, STC2, SPP1
GO:0007064~mitotic sister chromatid cohesion	3	6.3	0.987	CDC20, CDCA5, ESCO2
GO:0035094~response to nicotine	4	3.9	0.987	MT-ND4, CHRNA5, MMP2, SLC7A11
GO:0007596~blood coagulation	6	2.6	0.987	PROCR, THBD, SERPINA1, CD36, TFPI, F5
GO:1901796~regulation of signal transduction by p53 class mediator	4	3.8	0.987	SETD9, RFFL, AURKB, AURKA
GO:0071168~protein localization to chromatin	3	6.0	0.987	PLK1, ESCO2, EZH2
GO:0015813~L-glutamate transport	3	6.0	0.987	SLC38A6, SLC1A4, SLC7A11
GO:0010165~response to X-ray	3	6.0	0.987	THBD, BLM, RAD51
GO:0009060~aerobic respiration	5	2.9	0.987	MT-ND4L, MT-ND4, MT-ND5, MT-CO1, MT-CO3
GO:0016477~cell migration	12	1.7	0.987	LIMA1, NCKAP1, VTN, DEPDC1B, LAMB3, MMP2, SHROOM2, CDK1, PIK3CD, CTNNA3, CORO7, PARP9
GO:0015804~neutral amino acid transport	3	5.7	0.987	SLC38A1, SLC1A5, SLC7A10
GO:0002526~acute inflammatory response	3	5.7	0.987	NLRP3, NUPR1, TREM1
GO:1905322~positive regulation of mesenchymal stem cell migration	2	19.9	0.987	ACKR3, FBXO5
GO:0000915~actomyosin contractile ring assembly	2	19.9	0.987	ANLN, RACGAP1
GO:0017085~response to insecticide	2	19.9	0.987	FECH, MAP1B
GO:0072757~cellular response to camptothecin	2	19.9	0.987	BLM, RAD51
GO:0032375~negative regulation of cholesterol transport	2	19.9	0.987	APOC2, APOC1
GO:1905448~positive regulation of mitochondrial ATP synthesis coupled electron transport	2	19.9	0.987	CCNB1, CDK1
GO:0007079~mitotic chromosome movement towards spindle pole	2	19.9	0.987	CENPE, DLGAP5
GO:0010916~negative regulation of very-low-density lipoprotein particle clearance	2	19.9	0.987	APOC2, APOC1
GO:2000479~regulation of cAMP-dependent protein kinase activity	2	19.9	0.987	NPFFR2, SESN2
GO:0016321~female meiosis chromosome segregation	2	19.9	0.987	PLK1, TTK
GO:0070098~chemokine-mediated signaling pathway	5	2.8	0.987	CCL7, CCL20, CCL4, ACKR3, CMKLR1

Red indicates FDR < 0.05.

Supplementary Table 6. Pathways predicted by Reactome

Pathways enriched from DEGs in HLF cells

Term	Count	Fold Enric	FDR	Genes
R-HSA-909733~Interferon alpha/beta signaling	11	36.7	0.000	ISG20, IFITM3, OAS1, STAT1, OAS2, MX1, IFIT5, ISG15, IFI35, XAF1, SAMHD1
R-HSA-913531~Interferon Signaling	14	13.4	0.000	IFITM3, VCAM1, STAT1, MX1, IFIT5, ISG15, IFI35, SAMHD1, ISG20, HERC5, OAS1, OAS2, TRIM14, XAF1
R-HSA-1280215~Cytokine Signaling in Immune system	17	5.5	0.000	IFITM3, VCAM1, SERPINB2, STAT1, MX1, IFIT5, ISG15, IFI35, SAMHD1, ISG20, HERC5, CXCL10, OAS1, OAS2, TRIM14, XAF1, BCL2L1
R-HSA-168256~Immune System	20	2.5	0.004	IFITM3, CD74, VCAM1, SERPINB2, STAT1, HSPA6, MX1, IFIT5, ISG15, IFI35, SAMHD1, IFIH1, ISG20, HERC5, CXCL10, OAS1, OAS2, TRIM14, XAF1, BCL2L1
R-HSA-1169410~Antiviral mechanism by IFN-stimulated genes	6	10.3	0.012	HERC5, OAS1, STAT1, OAS2, MX1, ISG15
R-HSA-877300~Interferon gamma signaling	5	13.4	0.020	VCAM1, OAS1, STAT1, OAS2, TRIM14
R-HSA-1169408~ISG15 antiviral mechanism	4	14.1	0.097	HERC5, STAT1, MX1, ISG15
R-HSA-9833110~RSV-host interactions	4	10.5	0.194	IFIH1, HERC5, OAS2, ISG15
R-HSA-936440~Negative regulators of DDX58/IFIH1 signaling	3	22.0	0.221	IFIH1, HERC5, ISG15
R-HSA-9820952~Respiratory Syncytial Virus Infection Pathway	4	8.6	0.270	IFIH1, HERC5, OAS2, ISG15
R-HSA-9833482~PKR-mediated signaling	3	10.3	0.664	HERC5, STAT1, ISG15
R-HSA-8983711~OAS antiviral response	2	57.1	0.664	OAS1, OAS2
R-HSA-9029558~NR1H2 & NR1H3 regulate gene expression linked to lipc	2	57.1	0.664	SREBF1, FASN
R-HSA-168928~DDX58/IFIH1-mediated induction of interferon-alpha/beta	3	9.4	0.708	IFIH1, HERC5, ISG15
R-HSA-6785807~Interleukin-4 and Interleukin-13 signaling	3	7.1	1.000	VCAM1, STAT1, BCL2L1
R-HSA-9679506~SARS-CoV Infections	5	3.1	1.000	IFIH1, STAT1, ISG15, PARP9, BCL2L1
R-HSA-9705671~SARS-CoV-2 activates/modulates innate and adaptive imr	3	6.1	1.000	IFIH1, STAT1, ISG15
R-HSA-9824446~Viral Infection Pathways	7	2.2	1.000	IFIH1, HERC5, STAT1, OAS2, ISG15, PARP9, BCL2L1

Pathways enriched from DEGs in macrophages

Term	Count	Fold Enric	FDR	Genes
R-HSA-1640170~Cell Cycle	83	3.7	0.000	ERCC6L, FEN1, DSCC1, NCAPG2, GMNN, HJURP, H2AC19, BUB1B, FOXM1, H2AC18, LMNB1, SYNE1, CDC20, TUBA1B, TUBA1A, PTTG1, TUBB3, EXO1, H2BC21, NUF2, RCC1, NEK2, MYBL2, FBXO5, TK1, GTSE1, RMI2, KIF23, ESCO2, KNL1, CDC25C, CDC25A, CCNA2, CCNE2, ESPL1, INCENP, MCM3, BIRC5, MCM4, MCM5, KIF2C, KIF20A, MCM2, BLM, PCNA, CDCA5, NCAPG, CDCA8, HMMR, PKMYT1, TYMS, CENPA, NCAPH, AURKB, AURKA, SKA2, CCNB2, CCNB1, CDC45, ORC1, E2F2, CLSPN, CDKN2D, PLK4, GINS1, GINS2, CDT1, CENPW, UBE2C, PLK1, MND1, CDC7, NDC80, ZWINT, CENPE, TPX2, KIF18A, RAD51, CDK1, CENPQ, SPC24, SPC25, MAD2L1

R-HSA-69278~Cell Cycle, Mitotic	73	4.0	0.000	ERCC6L, FEN1, NCAPG2, GMNN, H2AC19, BUB1B, FOXM1, H2AC18, LMNB1, CDC20, TUBA1B, TUBA1A, PTTG1, TUBB3, H2BC21, NUF2, RCC1, NEK2, MYBL2, FBXO5, TK1, GTSE1, KIF23, ESCO2, KNL1, CDC25C, CDC25A, CCNA2, CCNE2, ESPL1, INCENP, MCM3, BIRC5, MCM4, MCM5, KIF2C, KIF20A, MCM2, PCNA, CDCA5, NCAPG, CDCA8, HMMR, PKMYT1, TYMS, CENPA, NCAPH, AURKB, AURKA, SKA2, CCNB2, CCNB1, CDC45, ORC1, E2F2, CDKN2D, PLK4, GINS1, GINS2, CDT1, UBE2C, PLK1, CDC7, NDC80, ZWINT, CENPE, TPX2, KIF18A, CDK1, CENPQ, SPC24, SPC25, MAD2L1
R-HSA-69620~Cell Cycle Checkpoints	43	4.5	0.000	BLM, ERCC6L, CDCA8, BUB1B, PKMYT1, CENPA, AURKB, SKA2, CDC20, CCNB2, CCNB1, CDC45, ORC1, EXO1, H2BC21, NUF2, CLSPN, GTSE1, RMI2, UBE2C, PLK1, CDC7, KNL1, CDC25C, CDC25A, NDC80, ZWINT, CCNA2, CENPE, KIF18A, CCNE2, INCENP, MCM3, CDK1, BIRC5, MCM4, MCM5, KIF2C, CENPQ, SPC24, MCM2, MAD2L1, SPC25
R-HSA-2500257~Resolution of Sister Chromatid Cohesion	28	6.8	0.000	ERCC6L, CDCA5, CDCA8, BUB1B, CENPA, AURKB, SKA2, CDC20, CCNB2, TUBA1B, CCNB1, TUBA1A, TUBB3, NUF2, PLK1, KNL1, NDC80, ZWINT, CENPE, KIF18A, INCENP, CDK1, BIRC5, KIF2C, CENPQ, SPC24, MAD2L1, SPC25
R-HSA-68877~Mitotic Prometaphase	32	4.8	0.000	ERCC6L, CDCA5, NCAPG, CDCA8, BUB1B, CENPA, NCAPH, AURKB, SKA2, CDC20, CCNB2, TUBA1B, CCNB1, TUBA1A, TUBB3, NUF2, NEK2, PLK4, PLK1, KNL1, NDC80, ZWINT, CENPE, KIF18A, INCENP, CDK1, BIRC5, KIF2C, CENPQ, SPC24, MAD2L1, SPC25
R-HSA-2555396~Mitotic Metaphase and Anaphase	34	4.4	0.000	ERCC6L, CDCA5, CDCA8, BUB1B, CENPA, AURKB, SKA2, LMNB1, CDC20, CCNB2, TUBA1B, CCNB1, TUBA1A, PTTG1, TUBB3, NUF2, RCC1, FBXO5, UBE2C, PLK1, KNL1, NDC80, ZWINT, CENPE, KIF18A, ESPL1, INCENP, CDK1, BIRC5, KIF2C, CENPQ, SPC24, MAD2L1, SPC25
R-HSA-9648025~EML4 and NUDC in mitotic spindle formation	24	6.3	0.000	ERCC6L, PLK1, CDCA8, BUB1B, KNL1, CENPA, NDC80, ZWINT, AURKB, SKA2, CDC20, CENPE, TUBA1B, KIF18A, TUBA1A, INCENP, TUBB3, NUF2, BIRC5, KIF2C, CENPQ, SPC24, MAD2L1, SPC25
R-HSA-68882~Mitotic Anaphase	33	4.3	0.000	ERCC6L, CDCA5, CDCA8, BUB1B, CENPA, AURKB, SKA2, LMNB1, CDC20, CCNB2, TUBA1B, CCNB1, TUBA1A, PTTG1, TUBB3, NUF2, RCC1, UBE2C, PLK1, KNL1, NDC80, ZWINT, CENPE, KIF18A, ESPL1, INCENP, CDK1, BIRC5, KIF2C, CENPQ, SPC24, MAD2L1, SPC25
R-HSA-68886~M Phase	44	3.3	0.000	ERCC6L, CDCA5, NCAPG2, H2AC19, NCAPG, CDCA8, BUB1B, CENPA, NCAPH, AURKB, SKA2, H2AC18, LMNB1, CDC20, CCNB2, TUBA1B, CCNB1, TUBA1A, PTTG1, TUBB3, H2BC21, NUF2, RCC1, NEK2, FBXO5, PLK4, UBE2C, PLK1, KIF23, KNL1, NDC80, ZWINT, CENPE, KIF18A, ESPL1, INCENP, CDK1, BIRC5, KIF2C, KIF20A, CENPQ, SPC24, MAD2L1, SPC25
R-HSA-5663220~RHO GTPases Activate Formins	25	5.5	0.000	ERCC6L, CDCA8, BUB1B, CENPA, AURKB, SKA2, CDC20, TUBA1B, TUBA1A, TUBB3, NUF2, PLK1, KNL1, NDC80, ZWINT, CENPE, KIF18A, DIAPH3, INCENP, BIRC5, KIF2C, CENPQ, SPC24, MAD2L1, SPC25
R-HSA-141424~Amplification of signal from the kinetochores	21	6.7	0.000	ERCC6L, PLK1, CDCA8, BUB1B, KNL1, CENPA, NDC80, ZWINT, AURKB, SKA2, CDC20, CENPE, KIF18A, INCENP, NUF2, BIRC5, KIF2C, CENPQ, SPC24, MAD2L1, SPC25

R-HSA-141444~Amplification of signal from unattached kinetochores via a	21	6.7	0.000	ERCC6L, PLK1, CDCA8, BUB1B, KNL1, CENPA, NDC80, ZWINT, AURKB, SKA2, CDC20, CENPE, KIF18A, INCENP, NUF2, BIRC5, KIF2C, CENPQ, SPC24, MAD2L1, SPC25
R-HSA-69618~Mitotic Spindle Checkpoint	22	6.0	0.000	ERCC6L, UBE2C, PLK1, CDCA8, BUB1B, KNL1, CENPA, NDC80, ZWINT, AURKB, SKA2, CDC20, CENPE, KIF18A, INCENP, NUF2, BIRC5, KIF2C, CENPQ, SPC24, MAD2L1, SPC25
R-HSA-2467813~Separation of Sister Chromatids	28	4.5	0.000	ERCC6L, CDCA5, CDCA8, BUB1B, CENPA, AURKB, SKA2, CDC20, TUBA1B, TUBA1A, PTTG1, TUBB3, NUF2, UBE2C, PLK1, KNL1, NDC80, ZWINT, CENPE, KIF18A, ESPL1, INCENP, BIRC5, KIF2C, CENPQ, SPC24, MAD2L1, SPC25
R-HSA-983189~Kinesins	15	7.8	0.000	KIF23, KIF27, KIF15, CENPE, TUBA1B, KIF18A, TUBA1A, KIF18B, RACGAP1, TUBB3, KIFC1, KIF4A, KIF2C, KIF20A, KIF3C
R-HSA-195258~RHO GTPase Effectors	34	3.2	0.000	NCKAP1, ERCC6L, H2AC19, CDCA8, BUB1B, CENPA, IQGAP3, AURKB, SKA2, H2AC18, CDC20, TUBA1B, TUBA1A, TUBB3, H2BC21, NUF2, PLK1, KNL1, CDC25C, NDC80, ZWINT, CENPE, KIF18A, DIAPH3, PRC1, INCENP, NOXA1, BIRC5, KIF2C, CENPQ, SPC24, ROPN1, MAD2L1, SPC25
R-HSA-453279~Mitotic G1 phase and G1/S transition	21	4.3	0.000	CDKN2D, CDT1, PCNA, GMNN, CDC7, TYMS, CDC25A, CCNA2, CCNB1, CDC45, ORC1, CCNE2, MCM3, CDK1, MCM4, E2F2, MCM5, MYBL2, TK1, FBXO5, MCM2
R-HSA-156711~Polo-like kinase mediated events	8	15.4	0.000	CCNB2, CCNB1, PLK1, MYBL2, PKMYT1, FOXM1, CDC25C, CDC25A
R-HSA-69273~Cyclin A/B1/B2 associated events during G2/M transition	9	11.1	0.000	CCNA2, CCNB2, CCNB1, PLK1, CDK1, PKMYT1, FOXM1, CDC25C, CDC25A
R-HSA-176974~Unwinding of DNA	7	17.9	0.000	GINS1, GINS2, CDC45, MCM3, MCM4, MCM5, MCM2
R-HSA-69206~G1/S Transition	18	4.2	0.000	CDT1, PCNA, GMNN, CDC7, TYMS, CDC25A, CCNA2, CCNB1, CDC45, ORC1, CCNE2, MCM3, CDK1, MCM4, MCM5, TK1, FBXO5, MCM2
R-HSA-176187~Activation of ATR in response to replication stress	10	8.3	0.000	CDC45, ORC1, MCM3, MCM4, CDC7, MCM5, CLSPN, CDC25C, CDC25A, MCM2
R-HSA-6811434~COPI-dependent Golgi-to-ER retrograde traffic	15	4.7	0.000	KIF23, KIF27, KIF15, CENPE, TUBA1B, KIF18A, TUBA1A, KIF18B, RACGAP1, TUBB3, KIFC1, KIF4A, KIF2C, KIF20A, KIF3C
R-HSA-69190~DNA strand elongation	9	8.7	0.000	GINS1, GINS2, FEN1, PCNA, CDC45, MCM3, MCM4, MCM5, MCM2
R-HSA-69481~G2/M Checkpoints	19	3.5	0.000	BLM, RMI2, CDC7, PKMYT1, CDC25C, CDC25A, CCNB2, CCNB1, CDC45, ORC1, EXO1, H2BC21, MCM3, CDK1, MCM4, MCM5, CLSPN, GTSE1, MCM2
R-HSA-68962~Activation of the pre-replicative complex	9	8.4	0.000	CDT1, CDC45, ORC1, GMNN, MCM3, MCM4, CDC7, MCM5, MCM2
R-HSA-194315~Signaling by Rho GTPases	46	2.0	0.000	ARHGAP11A, NCKAP1, ERCC6L, H2AC19, ARHGAP18, CDCA8, BUB1B, SLC1A5, CENPA, IQGAP3, AURKB, SKA2, H2AC18, LMNB1, CDC20, TUBA1B, TUBA1A, RACGAP1, TUBB3, H2BC21, ARHGDIB, NUF2, TAGAP, PLK1, ARAP2, KNL1, CDC25C, NDC80, ZWINT, NET1, CENPE, ANLN, KIF18A, DEPDC1B, DIAPH3, PRC1, INCENP, NOXA1, BIRC5, KIF2C, CENPQ, SPC24, ROPN1, ARHGEF5, MAD2L1, SPC25
R-HSA-176417~Phosphorylation of Emi1	5	25.6	0.001	CDC20, CCNB1, PLK1, CDK1, FBXO5
R-HSA-9716542~Signaling by Rho GTPases, Miro GTPases and RHOBTE	46	2.0	0.001	ARHGAP11A, NCKAP1, ERCC6L, H2AC19, ARHGAP18, CDCA8, BUB1B, SLC1A5, CENPA, IQGAP3, AURKB, SKA2, H2AC18, LMNB1, CDC20, TUBA1B, TUBA1A, RACGAP1, TUBB3, H2BC21, ARHGDIB, NUF2, TAGAP, PLK1, ARAP2, KNL1, CDC25C, NDC80, ZWINT, NET1, CENPE, ANLN, KIF18A, DEPDC1B, DIAPH3, PRC1, INCENP, NOXA1, BIRC5, KIF2C, CENPQ, SPC24, ROPN1, ARHGEF5, MAD2L1, SPC25

R-HSA-69242~S Phase	18	3.4	0.001	GINS1, GINS2, CDT1, FEN1, PCNA, UBE2C, CDCA5, GMNN, ESCO2, CDC25A, CCNA2, CDC45, ORC1, CCNE2, MCM3, MCM4, MCM5, MCM2
R-HSA-174143~APC/C-mediated degradation of cell cycle proteins	13	4.6	0.001	UBE2C, PLK1, BUB1B, AURKB, AURKA, CCNA2, CDC20, CCNB1, PTTG1, CDK1, NEK2, FBXO5, MAD2L1
R-HSA-453276~Regulation of mitotic cell cycle	13	4.6	0.001	UBE2C, PLK1, BUB1B, AURKB, AURKA, CCNA2, CDC20, CCNB1, PTTG1, CDK1, NEK2, FBXO5, MAD2L1
R-HSA-69205~G1/S-Specific Transcription	8	8.8	0.001	CDT1, PCNA, CDC45, ORC1, CDK1, TK1, FBXO5, TYMS
R-HSA-69239~Synthesis of DNA	15	3.8	0.001	GINS1, GINS2, CDT1, FEN1, PCNA, UBE2C, GMNN, CCNA2, CDC45, ORC1, CCNE2, MCM3, MCM4, MCM5, MCM2
R-HSA-69306~DNA Replication	19	3.1	0.001	GINS1, GINS2, CDT1, FEN1, PCNA, UBE2C, GMNN, H2AC19, CDC7, H2AC18, CCNA2, CDC45, ORC1, CCNE2, H2BC21, MCM3, MCM4, MCM5, MCM2
R-HSA-69275~G2/M Transition	19	3.0	0.002	PLK4, PLK1, HMMR, PKMYT1, FOXM1, CDC25C, CDC25A, AURKA, CCNA2, CCNB2, TPX2, TUBA1B, CCNB1, TUBA1A, TUBB3, CDK1, NEK2, MYBL2, GTSE1
R-HSA-9648895~Response of EIF2AK1 (HRI) to heme deficiency	6	12.3	0.002	CEBPG, GRB10, ASNS, CHAC1, ATF5, ATF4
R-HSA-453274~Mitotic G2-G2/M phases	19	2.9	0.002	PLK4, PLK1, HMMR, PKMYT1, FOXM1, CDC25C, CDC25A, AURKA, CCNA2, CCNB2, TPX2, TUBA1B, CCNB1, TUBA1A, TUBB3, CDK1, NEK2, MYBL2, GTSE1
R-HSA-379716~Cytosolic tRNA aminoacylation	7	9.0	0.002	AARS1, SARS1, EPRS1, MARS1, IARS1, CARS1, WARS1
R-HSA-8856688~Golgi-to-ER retrograde transport	15	3.5	0.002	KIF23, KIF27, KIF15, CENPE, TUBA1B, KIF18A, TUBA1A, KIF18B, RACGAP1, TUBB3, KIFC1, KIF4A, KIF2C, KIF20A, KIF3C
R-HSA-983231~Factors involved in megakaryocyte development and platele	17	3.1	0.003	KIF23, KIF27, KIF15, CENPE, TUBA1B, KIF18A, TUBA1A, KIF18B, RACGAP1, TUBB3, KIFC1, KIF4A, MYB, KIF2C, KIF20A, KIF3C, PRKACB
R-HSA-909733~Interferon alpha/beta signaling	11	4.4	0.004	BST2, IFITM1, IRF7, IFI6, IFIT5, IRF8, ISG15, IFI35, XAF1, IRF9, OASL
R-HSA-109582~Hemostasis	38	1.9	0.006	ECM1, SERPINA1, SPARC, ITGAM, SLC7A11, SLC7A10, TFPI, TREM1, PIK3R6, KIF15, THBD, TUBA1B, TUBA1A, RACGAP1, TUBB3, MYB, KCNMB3, KIF3C, CD36, PRKACB, S100A10, SRGN, BRPF3, ITGA1, KIF23, GTPBP2, KIF27, F5, FGR, CENPE, PROCR, KIF18A, KIF18B, P2RX4, KIFC1, KIF4A, KIF2C, KIF20A
R-HSA-2514853~Condensation of Prometaphase Chromosomes	5	14.0	0.007	CCNB2, CCNB1, CDK1, NCAPG, NCAPH
R-HSA-69478~G2/M DNA replication checkpoint	4	24.6	0.007	CCNB2, CCNB1, CDK1, PKMYT1
R-HSA-176814~Activation of APC/C and APC/C:Cdc20 mediated degradat	10	4.0	0.016	CCNA2, CDC20, CCNB1, PTTG1, UBE2C, PLK1, CDK1, BUB1B, NEK2, MAD2L1
R-HSA-2980767~Activation of NIMA Kinases NEK9, NEK6, NEK7	4	17.6	0.022	CCNB2, CCNB1, PLK1, CDK1
R-HSA-1538133~G0 and Early G1	6	6.8	0.031	CCNA2, PCNA, CCNE2, CDK1, MYBL2, CDC25A
R-HSA-3700989~Transcriptional Regulation by TP53	24	2.0	0.032	FANCI, BLM, RMI2, PCNA, MT-CO1, SETD9, BRPF3, NLRC4, CDC25C, AURKB, AURKA, CCNA2, TPX2, CCNB1, CCNE2, EXO1, SESN2, CDK1, TCEA1, BIRC5, MT-CO2, MT-CO3, RFFL, TP73
R-HSA-73886~Chromosome Maintenance	13	2.9	0.038	BLM, FEN1, PCNA, CENPW, DSCC1, H2AC19, HJURP, KNL1, CENPA, H2AC18, CCNA2, H2BC21, CENPQ
R-HSA-2132295~MHC class II antigen presentation	12	3.0	0.040	CENPE, TUBA1B, KIF18A, TUBA1A, RACGAP1, TUBB3, KIF4A, KIF23, KIF2C, KIF20A, KIF3C, KIF15
R-HSA-379724~tRNA Aminoacylation	7	5.1	0.040	AARS1, SARS1, EPRS1, MARS1, IARS1, CARS1, WARS1

R-HSA-6804114~TP53 Regulates Transcription of Genes Involved in G2 C	5	8.5	0.042	CCNB1, PCNA, CDK1, CDC25C, AURKA
R-HSA-6811442~Intra-Golgi and retrograde Golgi-to-ER traffic	16	2.4	0.042	KIF23, KIF27, KIF15, CENPE, TUBA1B, KIF18A, TUBA1A, KIF18B, RACGAP1, MAN2A2, TUBB3, KIFC1, KIF4A, KIF2C, KIF20A, KIF3C
R-HSA-69052~Switching of origins to a post-replicative state	10	3.4	0.047	CCNA2, CDT1, ORC1, CCNE2, UBE2C, GMNN, MCM3, MCM4, MCM5, MCM2
R-HSA-176409~APC/C:Cdc20 mediated degradation of mitotic proteins	9	3.7	0.049	CCNA2, CDC20, CCNB1, PTTG1, UBE2C, CDK1, BUB1B, NEK2, MAD2L1
R-HSA-176408~Regulation of APC/C activators between G1/S and early an	9	3.5	0.072	CCNA2, CDC20, CCNB1, UBE2C, PLK1, CDK1, BUB1B, FBXO5, MAD2L1
R-HSA-6791312~TP53 Regulates Transcription of Cell Cycle Genes	7	4.5	0.072	CCNA2, CCNB1, PCNA, CCNE2, CDK1, CDC25C, AURKA
R-HSA-69002~DNA Replication Pre-Initiation	13	2.5	0.086	CDT1, UBE2C, GMNN, H2AC19, CDC7, H2AC18, CDC45, ORC1, H2BC21, MCM3, MCM4, MCM5, MCM2
R-HSA-913531~Interferon Signaling	18	2.1	0.106	IFITM1, IFI6, IFIT5, ISG15, IFI35, OASL, BST2, TUBA1B, TUBA1A, TUBB3, IRF7, CDK1, IRF8, XAF1, HSPA1B, IRF9, HSPA1A, TRIM34
R-HSA-179409~APC-Cdc20 mediated degradation of Nek2A	5	6.2	0.126	CDC20, UBE2C, BUB1B, NEK2, MAD2L1
R-HSA-8980692~RHOA GTPase cycle	12	2.5	0.128	ARHGAP11A, NET1, ANLN, DEPDC1B, DIAPH3, RACGAP1, TAGAP, ARHGDIB, ARAP2, ARHGAP18, IQGAP3, ARHGEF5
R-HSA-774815~Nucleosome assembly	8	3.4	0.136	CENPW, H2BC21, H2AC19, HJURP, KNL1, CENPA, CENPQ, H2AC18
R-HSA-606279~Deposition of new CENPA-containing nucleosomes at the	8	3.4	0.136	CENPW, H2BC21, H2AC19, HJURP, KNL1, CENPA, CENPQ, H2AC18
R-HSA-8854518~AURKA Activation by TPX2	8	3.4	0.136	PLK4, TPX2, TUBA1A, PLK1, CDK1, NEK2, HMMR, AURKA
R-HSA-162658~Golgi Cisternae Pericentriolar Stack Reorganization	4	8.8	0.137	CCNB2, CCNB1, PLK1, CDK1
R-HSA-8953897~Cellular responses to stimuli	40	1.5	0.139	SMARCD3, MT-CO1, EPAS1, CEBPG, H2AC19, SLC7A11, NPAS2, H2AC18, LMNB1, TUBA1B, TUBA1A, TUBB3, H2BC21, RPS6KA2, SESN2, GRB10, E2F2, NLRP3, CHAC1, TBL1X, CDKN2D, ABCC3, UBE2C, HSPA6, ASNS, WIPI1, CCNA2, CCNE2, ID1, MT1F, MT1G, MT-CO2, MT-CO3, ATF5, SQSTM1, HSPA1B, MT1E, ATF4, EZH2, HSPA1A
R-HSA-2995410~Nuclear Envelope (NE) Reassembly	8	3.3	0.150	CCNB2, TUBA1B, CCNB1, TUBA1A, TUBB3, CDK1, RCC1, LMNB1
R-HSA-8852276~The role of GTSE1 in G2/M progression after G2 checkp	8	3.2	0.170	CCNB2, TUBA1B, CCNB1, TUBA1A, TUBB3, PLK1, CDK1, GTSE1
R-HSA-73894~DNA Repair	20	1.8	0.176	FANCI, BLM, FEN1, RMI2, PCNA, H2AC19, ISG15, H2AC18, CCNA2, NEIL3, RAD51, EXO1, UBE2T, UVSSA, H2BC21, PCLAF, TCEA1, APBB1, CLSPN, DTL
R-HSA-4615885~SUMOylation of DNA replication proteins	6	4.1	0.199	PCNA, INCENP, CDCA8, BIRC5, AURKB, AURKA
R-HSA-5633007~Regulation of TP53 Activity	12	2.3	0.200	CCNA2, TPX2, BLM, RMI2, BRPF3, EXO1, SETD9, CDK1, RFFL, AURKB, AURKA, TP73
R-HSA-168256~Immune System	84	1.3	0.207	CD86, DET1, NCKAP1, IFITM1, IL1RN, SERPINA1, ITGAM, FBXO27, CCNF, IFIT5, PIK3CD, IFI35, TREM1, KIF15, LMNB1, OASL, CDC20, HK3, LGALS3, TUBA1B, FCGR3A, TUBA1A, TUBB3, RPS6KA2, FTH1, GRB10, MUC12, CD36, PRKACB, HAVCR2, HERC6, FBXW4, MYLIP, MMP2, KIF23, GAB2, PDCD1LG2, RNASE2, FBXO10, FGR, IRF7, IRF8, BIRC5, TLR8, KIF2C, KIF20A, SQSTM1, IRF9, IFI6, LILRA1, NLRC4, FBXO44, RASGRP3, VTN, RACGAP1, CCL4, S100A12, NLRP3, CD14, KIF3C, PAG1, ATP8B4, SIGLEC15, UBE2C, CCL20, HSPA6, GGH, ISG15, LILRB2, LYZ, BST2, CENPE, KIF18A, FABP5, KIF4A, CDK1, SIGLEC1, HPSE, XAF1, IL7R, ULBP1, HSPA1B, TRIM34, HSPA1A

R-HSA-1280218~Adaptive Immune System	37	1.5	0.207	CD86, DET1, IFITM1, FBXO27, CCNF, PIK3CD, LILRA1, TREM1, FBXO44, KIF15, RASGRP3, CDC20, TUBA1B, FCGR3A, TUBA1A, RACGAP1, TUBB3, KIF3C, CD14, CD36, PRKACB, PAG1, HERC6, FBXW4, MYLIP, UBE2C, KIF23, LILRB2, PDCD1LG2, FBXO10, CENPE, KIF18A, KIF4A, KIF2C, KIF20A, SIGLEC1, ULBP1
R-HSA-68867~Assembly of the pre-replicative complex	11	2.4	0.210	CDT1, ORC1, UBE2C, H2BC21, GMNN, H2AC19, MCM3, MCM4, MCM5, H2AC18, MCM2
R-HSA-9024446~NR1H2 and NR1H3-mediated signaling	6	3.9	0.221	SREBF1, MYLIP, SCD, APOC2, APOC1, TBL1X
R-HSA-352230~Amino acid transport across the plasma membrane	5	4.7	0.264	SLC38A1, SLC1A4, SLC7A11, SLC1A5, SLC7A10
R-HSA-2995383~Initiation of Nuclear Envelope (NE) Reformation	4	6.5	0.271	CCNB2, CCNB1, CDK1, LMNB1
R-HSA-176412~Phosphorylation of the APC/C	4	6.5	0.271	CCNB1, UBE2C, PLK1, CDK1
R-HSA-2565942~Regulation of PLK1 Activity at G2/M Transition	8	2.8	0.287	PLK4, CCNB2, CCNB1, TUBA1A, PLK1, CDK1, NEK2, AURKA
R-HSA-141430~Inactivation of APC/C via direct inhibition of the APC/C co	4	6.2	0.297	CDC20, UBE2C, BUB1B, MAD2L1
R-HSA-141405~Inhibition of the proteolytic activity of APC/C required for	4	6.2	0.297	CDC20, UBE2C, BUB1B, MAD2L1
R-HSA-9013026~RHOB GTPase cycle	7	3.1	0.297	NET1, ANLN, DEPDC1B, DIAPH3, RACGAP1, IQGAP3, ARHGEF5
R-HSA-68949~Orc1 removal from chromatin	7	3.0	0.312	CCNA2, CDT1, ORC1, MCM3, MCM4, MCM5, MCM2
R-HSA-162582~Signal Transduction	101	1.2	0.314	NPFFR2, CD86, NCKAP1, ERCC6L, SPARC, H2AC19, BUB1B, H2AC18, CDC20, RGS3, RGS1, TUBB3, RPS6KA2, ADORA3, STMN1, ARHGDIB, MYB, PDK4, GRB10, PDE8A, PRKACB, CMKLR1, MYLIP, KNL1, CDC25C, PYGO1, DEPDC1B, SQSTM1, ROPN1, NSMAF, EPAS1, SH2D2A, CDCA8, ARHGAP18, FPR3, SLC1A5, IQGAP3, PIK3R6, SKA2, RASGRP3, RACGAP1, PAG1, FAM83A, ARHGEF35, PLK1, ZWINT, NDC80, NET1, ANLN, KIF18A, DIAPH3, NOXA1, ID1, CDK1, EZH2, ARHGAP11A, GPSM2, HTR2C, PIK3CD, LMNB1, TUBA1B, TUBA1A, UCN2, H2BC21, TAGAP, NUF2, PDE4B, DLGAP5, TAS2R4, SREBF1, STRADB, MMP2, ARAP2, GAB2, PLCB4, INCENP, BIRC5, KIF2C, ARHGEF5, RFNG, CENPA, AURKB, CCL7, CCL4, SPP1, TBL1X, LAMB3, CCL20, CENPE, FABP5, PRC1, SCD, ADM2, APOC2, APOC1, TACC3, ACKR3, CENPQ, SPC24, SPC25, MAD2L1
R-HSA-5653656~Vesicle-mediated transport	32	1.5	0.330	SERPINA1, HIP1, SPARC, MIA2, KIF15, TUBA1B, ALS2CL, TUBA1A, RACGAP1, MAN2A2, TUBB3, FTH1, KIF3C, CD36, DENND2D, CD163, SGIP1, DENND2B, RAB3IP, SYTL1, KIF23, KIF27, F5, CENPE, MARCO, KIF18A, KIF18B, KIFC1, KIF4A, KIF2C, KIF20A, IL7R
R-HSA-2262752~Cellular responses to stress	37	1.4	0.330	SMARCD3, MT-CO1, EPAS1, CEBPG, H2AC19, SLC7A11, NPAS2, H2AC18, LMNB1, TUBA1B, TUBA1A, TUBB3, H2BC21, RPS6KA2, SESN2, GRB10, E2F2, NLRP3, CHAC1, TBL1X, CDKN2D, ABCC3, UBE2C, HSPA6, ASNS, WIPI1, CCNA2, CCNE2, ID1, MT-CO2, MT-CO3, ATF5, SQSTM1, HSPA1B, ATF4, EZH2, HSPA1A
R-HSA-179419~APC:Cdc20 mediated degradation of cell cycle proteins pri	7	2.9	0.332	CCNA2, CDC20, UBE2C, CDK1, BUB1B, NEK2, MAD2L1
R-HSA-2299718~Condensation of Prophase Chromosomes	7	2.9	0.332	CCNB1, H2BC21, PLK1, NCAPG2, H2AC19, CDK1, H2AC18
R-HSA-113507~E2F-enabled inhibition of pre-replication complex formatio	3	10.3	0.342	CCNB1, ORC1, CDK1
R-HSA-9013106~RHOC GTPase cycle	7	2.9	0.342	ANLN, DEPDC1B, DIAPH3, RACGAP1, ARHGAP18, IQGAP3, ARHGEF5
R-HSA-9013423~RAC3 GTPase cycle	8	2.6	0.342	NCKAP1, DEPDC1B, DIAPH3, RACGAP1, NOXA1, ARHGDIB, ARAP2, SLC1A5
R-HSA-9833482~PKR-mediated signaling	7	2.9	0.356	TUBA1B, TUBA1A, TUBB3, CDK1, ISG15, HSPA1B, HSPA1A
R-HSA-174048~APC/C:Cdc20 mediated degradation of Cyclin B	4	5.4	0.378	CDC20, CCNB1, UBE2C, CDK1

R-HSA-9709603~Impaired BRCA2 binding to PALB2	4	5.1	0.418	BLM, RMI2, RAD51, EXO1
R-HSA-9701193~Defective homologous recombination repair (HRR) due tc	4	4.9	0.440	BLM, RMI2, RAD51, EXO1
R-HSA-9704331~Defective HDR through Homologous Recombination Rep	4	4.9	0.440	BLM, RMI2, RAD51, EXO1
R-HSA-9704646~Defective HDR through Homologous Recombination Rep	4	4.9	0.440	BLM, RMI2, RAD51, EXO1
R-HSA-9701192~Defective homologous recombination repair (HRR) due tc	4	4.9	0.440	BLM, RMI2, RAD51, EXO1
R-HSA-2173782~Binding and Uptake of Ligands by Scavenger Receptors	5	3.7	0.440	MARCO, CD163, SPARC, FTH1, CD36
R-HSA-2426168~Activation of gene expression by SREBF (SREBP)	5	3.7	0.440	SREBF1, SMARCD3, SCD, ELOVL6, TBL1X
R-HSA-5661231~Metallothioneins bind metals	3	8.4	0.448	MT1F, MT1G, MT1E
R-HSA-1280215~Cytokine Signaling in Immune system	35	1.4	0.483	CD86, IFITM1, IL1RN, ITGAM, IFIT5, IFI6, PIK3CD, IFI35, LMNB1, OASL, TUBA1B, TUBA1A, TUBB3, RPS6KA2, CCL4, GRB10, S100A12, CD36, HAVCR2, CCL20, MMP2, ISG15, GAB2, BST2, IRF7, CDK1, IRF8, BIRC5, XAF1, IL7R, SQSTM1, IRF9, HSPA1B, TRIM34, HSPA1A
R-HSA-2559583~Cellular Senescence	12	1.9	0.487	CDKN2D, CCNA2, CCNE2, UBE2C, RPS6KA2, H2BC21, ID1, H2AC19, E2F2, H2AC18, LMNB1, EZH2
R-HSA-1368108~BMAL1:CLOCK,NPAS2 activates circadian gene expres	4	4.6	0.503	SMARCD3, NAMPT, TBL1X, NPAS2
R-HSA-5693554~Resolution of D-loop Structures through Synthesis-Deper	4	4.6	0.503	BLM, RMI2, RAD51, EXO1
R-HSA-9707564~Cytoprotection by HMOX1	6	2.9	0.503	SMARCD3, MT-CO1, NLRP3, MT-CO2, MT-CO3, TBL1X
R-HSA-168330~Viral RNP Complexes in the Host Cell Nucleus	2	30.8	0.558	HSPA1B, HSPA1A
R-HSA-68881~Mitotic Metaphase/Anaphase Transition	2	30.8	0.558	PLK1, FBXO5
R-HSA-75035~Chk1/Chk2(Cds1) mediated inactivation of Cyclin B:Cdk1 c	3	7.1	0.558	CCNB1, CDK1, CDC25C
R-HSA-68884~Mitotic Telophase/Cytokinesis	3	7.1	0.558	PLK1, KIF23, KIF20A
R-HSA-437239~Recycling pathway of L1	5	3.3	0.558	TUBA1B, TUBA1A, TUBB3, RPS6KA2, KIF4A
R-HSA-157579~Telomere Maintenance	8	2.2	0.608	CCNA2, BLM, FEN1, PCNA, DSCC1, H2BC21, H2AC19, H2AC18
R-HSA-5660526~Response to metal ions	3	6.6	0.622	MT1F, MT1G, MT1E
R-HSA-196807~Nicotinate metabolism	4	4.0	0.645	PARP6, NMRK1, NAMPT, PARP9
R-HSA-6804756~Regulation of TP53 Activity through Phosphorylation	7	2.3	0.645	CCNA2, TPX2, BLM, RMI2, EXO1, AURKB, AURKA
R-HSA-9013149~RAC1 GTPase cycle	11	1.8	0.645	NCKAP1, DEPDC1B, DIAPH3, RACGAP1, TAGAP, NOXA1, ARHGDIB, ARAP2, SLC1A5, IQGAP3, ARHGEF5
R-HSA-5693538~Homology Directed Repair	9	2.0	0.648	CCNA2, BLM, FEN1, RMI2, RAD51, PCNA, EXO1, H2BC21, CLSPN
R-HSA-9675135~Diseases of DNA repair	5	3.0	0.648	BLM, RMI2, NEIL3, RAD51, EXO1
R-HSA-180786~Extension of Telomeres	5	3.0	0.648	CCNA2, BLM, FEN1, PCNA, DSCC1
R-HSA-4419969~Depolymerization of the Nuclear Lamina	3	6.2	0.648	CCNB1, CDK1, LMNB1
R-HSA-174184~Cdc20:Phospho-APC/C mediated degradation of Cyclin A	6	2.6	0.648	CCNA2, CDC20, UBE2C, CDK1, BUB1B, MAD2L1
R-HSA-5626467~RHO GTPases activate IQGAPs	4	3.8	0.648	TUBA1B, TUBA1A, TUBB3, IQGAP3
R-HSA-69473~G2/M DNA damage checkpoint	7	2.3	0.648	BLM, CCNB1, RMI2, EXO1, H2BC21, CDK1, CDC25C
R-HSA-380320~Recruitment of NuMA to mitotic centrosomes	7	2.3	0.648	PLK4, TUBA1B, TUBA1A, TUBB3, PLK1, CDK1, NEK2
R-HSA-1989781~PPARA activates gene expression	8	2.1	0.648	SREBF1, SMARCD3, ACSL1, G0S2, PLIN2, CD36, TBL1X, NPAS2
R-HSA-1500620~Meiosis	8	2.1	0.648	BLM, RAD51, H2BC21, H2AC19, MND1, H2AC18, SYNE1, LMNB1
R-HSA-2980766~Nuclear Envelope Breakdown	5	3.0	0.648	CCNB2, CCNB1, PLK1, CDK1, LMNB1

R-HSA-174178~APC/C:Cdh1 mediated degradation of Cdc20 and other AP	6	2.5	0.648	CDC20, PTTG1, UBE2C, PLK1, AURKB, AURKA
R-HSA-68875~Mitotic Prophase	9	2.0	0.648	CCNB2, CCNB1, H2BC21, PLK1, NCAPG2, H2AC19, CDK1, H2AC18, LMNB1
R-HSA-199991~Membrane Trafficking	28	1.4	0.648	SERPINA1, HIP1, MIA2, KIF15, TUBA1B, ALS2CL, TUBA1A, RACGAP1, MAN2A2, TUBB3, FTH1, KIF3C, DENND2D, SGIP1, DENND2B, RAB3IP, SYTL1, KIF23, KIF27, F5, CENPE, KIF18A, KIF18B, KIFC1, KIF4A, KIF2C, KIF20A, IL7R
R-HSA-400206~Regulation of lipid metabolism by PPARalpha	8	2.1	0.668	SREBF1, SMARCD3, ACSL1, G0S2, PLIN2, CD36, TBL1X, NPAS2
R-HSA-1362300~Transcription of E2F targets under negative control by p1(	3	5.8	0.674	CCNA2, CDK1, MYBL2
R-HSA-170145~Phosphorylation of proteins involved in the G2/M transitio:	2	20.5	0.675	CCNA2, CDK1
R-HSA-5693532~DNA Double-Strand Break Repair	10	1.8	0.678	CCNA2, BLM, FEN1, RMI2, RAD51, PCNA, EXO1, H2BC21, APBB1, CLSPN
R-HSA-174417~Telomere C-strand (Lagging Strand) Synthesis	4	3.6	0.678	BLM, FEN1, PCNA, DSCC1
R-HSA-5693568~Resolution of D-loop Structures through Holliday Junctio	4	3.6	0.678	BLM, RMI2, RAD51, EXO1

Red indicates FDR < 0.05.

Supplementary Table 7. Transcription factors predicted by iRegulon

Transcription factors predicted by DEGs upregulated in HLF cells

Rank	Track id	AUC	NES	ClusterCode	Transcription factor	Target genes
1	GSM1208780_batch2_chrom1_LoVo_NKX2.2_PassedQC_peaks_hg19	0.161	5.054	T1	NKX2.2	PSAT1,ASNS,FKBP14
2	wgEncodeHaibTfbsA549MaxV0422111PkRep2.broadPeak.gz	0.150	4.651	T2	MAX	PSAT1,SYCE2
3	wgEncodeSydhTfbsHepg2CebpbForsklnStdPk.narrowPeak.gz	0.146	4.532	T3	CEBPB	PSAT1,SLC7A2
4	wgEncodeHaibTfbsK562Stat5asc74442V0422111PkRep1.broadPeak.gz	0.138	4.249	T4	STAT5A	RELN,SLC7A2
5	wgEncodeHaibTfbsK562Trim28sc81411V0422111PkRep2.broadPeak.gz	0.133	4.074	T5	TRIM28	RELN,SLC7A2
6	wgEncodeSydhTfbsK562Gata1bIggmusPk.narrowPeak.gz	0.132	4.050	T6	GATA1	SLC7A2,ABLIM3,RELN,PAPPA2,GABRG1,ABCA10,SYCE2
7	wgEncodeSydhTfbsH1hescUsf2IggrabPk.narrowPeak.gz	0.132	4.038	T7	USF2	PAPPA2,RELN
8	wgEncodeSydhTfbsHelas3Prdm19115IggrabPk.narrowPeak.gz	0.132	4.034	T8	PRDM1	ABLIM3,SLC7A2,SLIT2,ABCA10,PSAT1
9	GSM1208731_batch2_chrom1_LoVo_E2F8_PassedQC_peaks_hg19	0.129	3.926	T9	E2F8	PSAT1,ASNS
10	wgEncodeHaibTfbsK562Stat5asc74442V0422111PkRep2.broadPeak.gz	0.129	3.914	T4	STAT5A	RELN,SLC7A2
11	wgEncodeHaibTfbsK562Gata2sc267Pcr1xPkRep2.broadPeak.gz	0.128	3.902	T10	GATA2	RELN,SLC7A2
12	wgEncodeSydhTfbsPbdefetalGata1UcdPk.narrowPeak.gz	0.128	3.898	T6	GATA1	GPR156,SLC7A2,RELN,ASNS,SLIT2,ABLIM3,GABRG1
13	wgEncodeUchicagoTfbsK562Egata2ControlPk.narrowPeak.gz	0.121	3.639	T10	GATA2	RELN,SLC7A2,SYCE2
14	wgEncodeHaibTfbsK562Tead4sc101184V0422111PkRep2.broadPeak.gz	0.117	3.528	T11	TEAD4	SLC7A2,RELN
15	wgEncodeHaibTfbsHct116Rad21V0422111PkRep1.broadPeak.gz	0.113	3.392	T12	RAD21	ABCA10,GPR156
16	wgEncodeSydhTfbsHelas3CfosStdPk.narrowPeak.gz	0.113	3.376	T13	FOS	SERPINB2,ABLIM3,FKBP14,PSAT1
17	wgEncodeSydhTfbsK562Irf1Ifna6hStdPk.narrowPeak.gz	0.111	3.317	T14	IRF1	RELN,ASNS
18	wgEncodeHaibTfbsGm12878Rad21V0416101PkRep1.broadPeak.gz	0.106	3.125	T12	RAD21	ABCA10,GPR156
19	wgEncodeSydhTfbsHuvecCfosUcdPk.narrowPeak.gz	0.105	3.106	T13	FOS	ABLIM3,SERPINB2,SLC7A2
20	wgEncodeSydhTfbsHelas3CmycStdPk.narrowPeak.gz	0.105	3.086	T15	MYC	SERPINB2,ABLIM3

Transcription factors predicted by DEGs downregulated in HLF cells

Rank	Track id	AUC	NES	ClusterCode	Transcription factor	Target genes
1	wgEncodeSydhTfbsK562Stat2Ifna6hStdPk.narrowPeak.gz	0.464	14.397	T1	STAT2	PARP9,IFIT5,MX1,OAS2,IFITM3,EPSTI1,PLSCR1,HERC5,XAF1,OAS1,IFI44L,ISG15,IFIH1,HSH2D,STAT1,CMPK2,SP110,IFI35,TMEM140,LAP3,SAMHD1,ISG20,TRIM14
2	wgEncodeSydhTfbsK562Stat1Ifna6hStdPk.narrowPeak.gz	0.448	13.877	T2	STAT1	PARP9,OAS2,MX1,IFIT5,EPSTI1,ISG15,PLSCR1,OAS1,LAP3,TRIM14,IFITM3,STAT1,XAF1,IFI44L,IFIH1,TMEM140,SP110,HSH2D,CMPK2,IFI35,SAMHD1,ISG20
3	wgEncodeSydhTfbsK562Stat2Ifna30StdPk.narrowPeak.gz	0.391	12.013	T1	STAT2	MX1,PARP9,EPSTI1,SP110,STAT1,IFIT5,IFIH1,PLSCR1,LAP3,ISG15,CMPK2,OAS1,IFITM3,TMEM140,XAF1,IFI35,OAS2,TRIM14,SAMHD1
4	wgEncodeSydhTfbsK562Stat1Ifna30StdPk.narrowPeak.gz	0.350	10.697	T2	STAT1	PARP9,MX1,STAT1,PLSCR1,CMPK2,ISG15,SP110,LAP3,IFIT5,EPSTI1,IFIH1,TMEM140,OAS1,IFITM3,IFI35,TRIM14,SAMHD1
5	wgEncodeSydhTfbsK562Irf1Ifna30StdPk.narrowPeak.gz	0.198	5.755	T3	IRF1	MX1,PARP9,IFIH1,IFI35,TMEM140,STAT1,ISG15,SP110,SAMHD1,IFIT5,XAF1,IFITM3,PLSCR1,LPCAT3,CMPK2,ANKRD37
6	wgEncodeSydhTfbsK562Stat1Ifng6hStdPk.narrowPeak.gz	0.144	4.011	T2	STAT1	PARP9,STAT1,LAP3,EPSTI1,CXCL10,TMEM140,PLSCR1,E2F1,IFI44L,IFI35

Transcription factors predicted by DEGs upregulated in macrophages

Rank	Track id	AUC	NES	ClusterCode	Transcription factor	Target genes

1	GSM1208731_batch2_chrom1_LoVo_E2F8_PassedQC_peaks_hg19	0.064	5.122	T1	E2F8	NFE2L1,RNF187,PSAT1,ASNS,SESN2,SLC7A11,CCPG1,TCEA1,SLC1A5,BEST1,CHAC1,GPT2,ULBP1,ZNF181,IFRD1,NCKAP1,NUPR1,PCK2,GTPBP2,SLC38A1,MTHFD2,WIPI1,XPOT,MKNK2,PHGDH,TSEN15,GRB10,INHBE
2	wgEncodeHaibTfbsK562Cebpbsc150V0422111PkRep2.broadPeak.gz	0.058	4.519	T2	CEBPB	CLGN,RFFL,SLC48A1,GRB10,SMARCD3,BEST1,FAM83A,EPAS1,TBL1X,ULBP1,INHBE,PYCR1,TNNC1,ATF5,NCKAP1,SLC38A1,MKNK2,XPOT,MTHFD2,PHGDH,RNF187,SLC7A11,NFE2L1,DHRS11,GTPBP2,RAB3IP,SESN2,CEBPG,ASNS,FTH1,CHAC1,HIP1,ZFAND3,TSEN15,GTF2IRD2B,GAB2,CCPG1,SLC1A4,SLC22A15,CBS,SQSTM1,ZNF181,SLC1A5,RPS6KA2,PNPLA1,MAP1B,TCEA1,NPFFR2,RUFY3,P2RX4,PAG1
3	wgEncodeSydhTfbsH1hescCebpbIggrabPk.narrowPeak.gz	0.054	4.080	T2	CEBPB	TMEM182,TNIP3,TBX15,SYBU,CBS,CHAC1,ULBP1,TNNC1,PYCR1,SESN2,MKNK2,TESK1,GPT2,FAM83A,PCDH1,ASNS,CACNA1F,PLCB4,MAP1B,SLC38A1,SLC1A5,INHBE,CLGN,ABCC3,NFE2L1,ZBED3,SQSTM1,MTHFD2,EPAS1,ELOVL6,TTLL4,ZFAND3,SLC7A11,PHGDH,GRB10,SNN,CYP4V2,PCDHAC2,BEST1,SLC48A1,PSAT1,GAB2,UNC13A,PYGO1,NUPR1,MAP7,XPOT,RNF187,ZFP41,NAMPT,MT1F,PCK2,ATF5,TBL1X,PPIL6,ADM2,SPIRE1,SMARCD3,ZDHHC1,GPCPD1,SNHG7,HIP1,ZNF853,GDF1,TSEN15,SLC1A4,P2RX4,SPATA18,UNC5B,NCKAP1,STC2,PRSS27,NPAS2,SLC7A10,SEL1L3,SLC2A10,RPS6KA2,MCOLN3,DET1,CEBPG,TMEM150A,DCAF10,RUFY3,SERPINB7,ALDH1L2,ERMAP,SPEG
4	wgEncodeHaibTfbsEcc1Cebpbsc150V0422111PkRep1.broadPeak.gz	0.054	4.007	T2	CEBPB	CHAC1,SLC48A1,CLGN,TNNC1,FAM83A,SLC38A1,EPAS1,MKNK2,TBL1X,PYCR1,RFFL,SLC7A11,CEBPG,RAB3IP,ALDH1L2,ZBED3,JDP2,SQSTM1,ABCC3,ZFAND3,PLCB4,PSAT1,ASNS,GRB10,SESN2,SPATA18,XPOT,INHBE,ULBP1,RPS6KA2,STRBP,CLIP4,BEST1,SLC1A4,FTH1,SLC1A5,NR1D2,MTHFD2,CBS,TSEN15,NCKAP1,SYBU,CYP4V2,DHRS12,SEL1L3,PHGDH,GPT2,IFRD1,PER3,MT1F,PNPLA1,RNF187,STC2,SMARCD3,AKAP5,DCAF10,FBXO27,P2RX4,NPL,UNC5B,ADM2
5	GSM1208598_batch1_chrom1_LoVo_CEBPB_PassedQC_peaks_hg19	0.053	3.977	T2	CEBPB	SLC38A1,MKNK2,XPOT,ZBED3,TNNC1,LGALS3,RNF187,ASNS,SESN2,CCPG1,EPAS1,CHAC1,RAB3IP,GRB10,SLC7A11,PSAT1,RFFL,FAM83A,ABCC3,IFRD1,SQSTM1,BEST1,GPT2,GTPBP2
6	wgEncodeHaibTfbsK562Cebpbsc150V0422111PkRep1.broadPeak.gz	0.053	3.964	T2	CEBPB	INHBE,CLGN,FAM83A,SLC7A11,BEST1,RFFL,TBL1X,GRB10,SLC38A1,TNNC1,SLC48A1,MKNK2,ULBP1,PYCR1,XPOT,MAN2A2,SESN2,NCKAP1,ASNS,CHAC1,DHRS11,EPAS1,SMARCD3,FTH1,GTPBP2,RNF187,NFE2L1,SEL1L3,ZNF181,MTHFD2,RAB3IP,SQSTM1,SLC1A5,CCPG1,TSEN15,ZFAND3,ATF5,PHGDH,CEBPG,ERMAP,NR1D2,SLC38A6,TLX2,CBS,TCEA1,P2RX4,HIP1,PCK2,PNPLA1,ELOVL6,KCNMB3,SLC1A4,FECH,TESK1,NR4A3

7	wgEncodeHaibTfbsHct116Cebpbsc150V0422111PkRep1.broadPeak.gz	0.050982	3.71627	T2	CEBPB	RFFL,FAM83A,CLGN,CLIP4,SLC7A11,TNNC1,CHAC1,RNF187,TBL1X,FLYWCH1,SLC22A15,NCKAP1,BEST1,SESN2,SLFN5,GTPBP2,MKNK2,XPOT,SLC38A1,NFE2L1,SLC48A1,TCEA1,MT1F,PYCR1,ULBP1,RAB3IP,SIPA1L2,CCPG1,EPAS1,INHBE,RPS6KA2,ZFAND3,ABCC3,FTH1
8	wgEncodeSydhTfbsH1hescBach1sc14700IggrabPk.narrowPeak.gz	0.0503215	3.64311	T3	BACH1	EPAS1,SLC30A1,GRB10,TESK1,CACNA1F,SQSTM1,SPIRE1,ULBP1,D2HGDH,FECH,SLC48A1,GTF2IRD2B,SNHG7,NAMPT,MKNK2,LRRC24,SLC7A11,FTH1,NPAS2,IFRD1,TMEM150A,PHF1,ERMAP,RNF187,SEL1L3,ACSL1,RFFL
9	wgEncodeSydhTfbsImr90CebpbIggrabPk.narrowPeak.gz	0.0497167	3.57612	T2	CEBPB	GPT2,SLC1A5,PRSS27,NUPR1,PNPLA1,BEST1,SLC7A10,CLGN,TNNC1,MTHFD2,CHAC1,PYCR1,SLC1A4,ELOVL6,PHGDH,TBL1X,SESN2,MKNK2,GAB2,NCKAP1,DENND2D,INHBE,ABCC3,ASNS,NFE2L1,PSAT1,EPAS1,PLCB4,RFFL,SLC38A1,CLIP4,GRB10,TNIP3,TTLL4,SLC48A1,CEBPG,SLC7A11,FLYWCH1,RNF187,PCDHAC2
10	GSM1208709_batch2_chrom1_LoVo_AEBP2_PassedQC_peaks_hg19	0.0493274	3.533	T4	AEBP2	MKNK2,PSAT1,FTH1,PCK2,CHAC1,ZFAND3,CCPG1,ARVCF,NUPR1,XPOT,GPT2,TCEA1,SESN2,BEST1,NFE2L1,ADM2,CEBPG,PHGDH,RNF187,C1QTNF6,MT1E,GTPBP2,MUC12,UNC5B,SMARCD3,SEL1L3,DHRS12,FBXW4
11	wgEncodeHaibTfbsA549Cebpbsc150V0422111PkRep1.broadPeak.gz	0.0492787	3.52761	T2	CEBPB	RFFL,ABCC3,EPAS1,TBL1X,CLGN,SLC7A11,PYCR1,CHAC1,SLC48A1,CLIP4,SLC38A1,ZBED3,TNNC1,FTH1,SLC1A5,GRB10,NCKAP1,SQSTM1,MKNK2,FAM83A,PLCB4,CEBPG,CAPN5,PSAT1,NUPR1,XPOT,ULBP1,BEST1,MTHFD2,RAB3IP,SDSL,FBXO27,DHRS12,NR1D2,ASNS,INHBE,ZFAND3,CBS,MUC12,ZNF181,TCEA1,GAB2,C1QTNF6,TESK1,IFRD1,KCTD15,AKAP5,KIF27,PCDHAC2,JDP2,TSEN15,TNIP3,SLC1A4,SMARCD3,ELOVL6,PYGO1,SESN2,NFE2L1,HIP1,PNPLA1,PCK2,PDE8A,BIRC7,NR4A3,MAP7,SIPA1L2,MT1F,STC2,ERMAP,TUBE1,TLX2,PDK4,CTNNA3,MAP1B,P2RX4,FLYWCH1,ZNF425,HEMK1,PER3,CCPG1,SLFN5,DTNB,BEND6,ALDH1L2
12	wgEncodeHaibTfbsA549Cebpbsc150V0422111PkRep2.broadPeak.gz	0.0484374	3.43443	T2	CEBPB	RFFL,SLC7A11,CLGN,CHAC1,SLC48A1,EPAS1,CLIP4,FAM83A,SLC38A1,GRB10,TBL1X,ABCC3,SQSTM1,FTH1,TNNC1,SLC1A5,INHBE,ZBED3,PLCB4,NCKAP1,MKNK2,PYCR1,NUPR1,RAB3IP,XPOT,TSEN15,KIF27,CBS,PNPLA1,MT1F,BEST1,TNIP3,PSAT1,ZFAND3,CEBPG,DHRS12,ULBP1,ELOVL6,SESN2,TESK1,AKAP5,MTHFD2,PDE8A,SMARCD3,PYGO1,SDSL,MUC12,SLC1A4,PCK2,BIRC7,HIP1,FBXO27,NR4A3,ASNS,PCDHAC2,SLC2A10,ZNF181

13	wgEncodeSydhTfbsHelas3CebpbIggrabPk.narrowPeak.gz	0.0476379	3.34588	T2	CEBPB	STRC,TRIM16L,TNNC1,SMARCD3,SQSTM1,RILPL1,ADM2,PER3,PYCR1,CLGN,ASNS,ZNF425,SLC38A1,SLC1A4,HIP1,MKNK2,NFE2L1,BEST1,DENND2D,ULBP1,INHBE,MT1F,JDP2,CEBPG,EPAS1,GAB2,P2RX4,PCDHAC2,GRB10,CHAC1,FLYWCH1,KCTD15,SLC7A10,CCDC112,SIPA1L2,PCK2,ELOVL6,SLC1A5,ZDHHC1,PSAT1,NUPR1,RNF187,GPT2,UNC13A,TBL1X,SLC22A15,MTHFD2,RAB3IP,NPAS2,PNPLA1,SEL1L3,SLC48A1,LGALS3,ZBED3,FGR,STC2,FTH1,NBPF1,FAM83A,RFFL,ABCC3,XPOT,DCAF10,MYO15B,NR1D2,GTPBP2
14	wgEncodeSydhTfbsK562Nfe2StdPk.narrowPeak.gz	0.0469844	3.27349	T5	NFE2	ERMAP,IFRD1,SLC48A1,FTH1,DET1,BEND6,CCPG1,SEL1L3,NR1D2,GAB2,SLC1A5,SMARCD3,SQSTM1,RFFL,SLC7A11
15	wgEncodeHaibTfbsEcc1Cebpbsc150V0422111PkRep2.broadPeak.gz	0.0447527	3.02631	T2	CEBPB	SLC48A1,CLGN,SLC7A11,FAM83A,RFFL,CHAC1,SLC38A1,SESN2,TNNC1,GRB10,RAB3IP,EPAS1,ALDH1L2,MKNK2,TBL1X,INHBE,ZBED3,NCKAP1,BEST1,SMARCD3,PLCB4,ZFAND3,FBXO27,CBS,ABCC3,JDP2,FTH1,SEL1L3,MT1F,ULBP1,XPOT,PNPLA1,PYCR1,SQSTM1,PSAT1,AKAP5,RPS6KA2,SPATA18,PHGDH,NUPR1,DHRS12,CLIP4,STRBP,NFE2L1,GPT2,MTHFD2,KIF27,SLC1A5,ASNS,ERMAP,RNF187,TSEN15,NPL,RUFY3,STC2,TNIP3,NLRC4

Transcription factors predicted by DEGs downregulated in macrophages

Rank	Track id	AUC	NES	ClusterCode	Transcription factor	Target genes
1	wgEncodeSydhTfbsHelas3E2f4StdPk.narrowPeak.gz	0.244247	11.5994	T1	E2F4	SPC25,DIAPH3,KIF20A,ESCO2,CENPW,KIF2C,NUF2,CDC25C,AURKB,ZWINT,MAD2L1,MCM3,PBK,CDC7,NDC80,UBE2T,ARHGAP11A,NEIL3,KIF18B,CDCA5,NCAPH,ATAD2,KIF15,PLK1,CDKN2D,TK1,CDC45,NEK2,SPC24,FOXM1,CDK1,KIF4A,PCNA,RAD54B,MELK,GTSE1,IQGAP3,SHCBP1,RAD54L,BUB1B,DTL,ANLN,MYBL2,BLM,SPAG5,CDCA3,CDT1,KIF18A,DLGAP5,HJURP,DEPDC1B,BIRC5,NCAPG2,TCF19,PRC1,HELLS,EXO1,TTK,NCAPG,INCENP,CDCA8,ESPL1,TPX2,ASF1B,TROAP,GGH,UBE2C,FEN1,FANCI,MCM4,WDR76,UHRF1,SKA2,CENPQ,GINS1,NET1,CENPA,KIF23,MND1,CLSPN,GINS2,NUSAP1,HMGB3,DSCC1,EZH2,ORC1,RACGAP1,TUBA1B,FBXO5,CCNA2,RCC1,TRIP13,RAD51,GMNN,CDC25A,KIFC1,TYMS,PLK4,LMNB1,ZNF367,MCM2,RGS3,AURKA,CDC20,CCNE2,MXD3,PKMYT1,E2F2,CORO7,ERCC6L,CDKN3

2	GSM1208691_batch1_chrom1_LoVo_TFDP1_PassedQC_peaks_hg19	0.202441	9.43286	T2	TFDP1	SKA2,CDK1,MCM5,NDC80,KIF23,PRC1,EXO1,NEK2,CDC25A,KIF18B,CDKN2D,FBXO5,PLK4,CDC7,FEN1,FANCI,INCENP,CDCA8,AURKB,ZWINT,NUF2,CDCA5,IQGAP3,DIAPH3,ESCO2,CLSPN,SPC24,ASF1B,CDC25C,TUBA1B,MCM3,BLM,PCNA,KIF2C,PBK,TK1,CDCA3,ATAD2,SPC25,BUB1B,HJURP,DTL,RAD51,GMNN,NCAPG,NUSAP1,KIF18A,SPAG5,TROAP,DLGAP5,UBE2C,UBE2T,CCNA2,NEIL3,CENPW,PLK1,MAD2L1,RAD54B,RACGAP1,CCNB1,CENPA,CDKN3,RAD54L,MXD3,E2F2,TYMS,KIF15,WDR76,ESPL1,SNRNP25,CCNB2,GINS1,CENPQ,BIRC5,GTSE1,KIF20A,CCNE2,TTK,SLC25A19,NET1,CDC20,ORC1,MELK,LMNB1,NCAPG2,KIF4A,SHCBP1,TPX2,ARHGAP11A,ZNF367,HMGB3,ERCC6L
3	wgEncodeSydhTfbsGm12878E2f4IggmusPk.narrowPeak.gz	0.191442	8.86286	T1	E2F4	ARHGAP11A,NCAPG2,IQGAP3,BUB1B,CDC20,ANLN,MYBL2,SPC24,TRIP13,DIAPH3,DEPDC1B,CDC25C,TROAP,RAD54L,AURKB,TACC3,NEK2,GGH,CDCA8,KIF4A,SPAG5,KIF20A,PRC1,HMGB3,FOXM1,KIF18B,GINS2,DLGAP5,BIRC5,ASF1B,KIF23,ESCO2,DSCC1,KIF2C,TTK,INCENP,CDCA5,PLK1,TK1,KIF15,HERC6,CDKN3,NCAPG,UBE2C,GTSE1,SPC25,CCNB2,CDKN2D,UHRF1,UBE2T,MND1,BLM,CDK1,CDC25A,CD86,PLK4,FEN1,ATAD2,NEIL3,FBXO5,CLSPN,NCAPH,SHCBP1,EXO1,PBK,ZWINT,MAD2L1,NDC80,OASL,ERCC6L,ZNF367,RAD51,CCNB1,MCM5,CENPW,CENPA,PKMYT1,CDCA3,CCNA2,MXD3,KIF18A,NUF2,GPSM2,TCF19,MELK,CKS2,SNRNP25,HJURP,TYMS,ESPL1,NUSAP1,RACGAP1,NET1,RCC1,MCM3,PCNA,MCM4,CDC7,FANCI,WDR76,CCNE2,E2F2,KIFC1,RAD54B,CENPQ,ID1,TPX2,HMMR,CDT1,TAL1,MCM2,SKA2,DTL,LMNB1,CCNF,RGS3,EZH2,TUBA1A,PTTG1
4	wgEncodeSydhTfbsK562E2f4UcdPk.narrowPeak.gz	0.190607	8.81955	T1	E2F4	DIAPH3,FOXM1,NCAPG2,BIRC5,TK1,NEIL3,NCAPG,IQGAP3,TRIP13,GINS2,TACC3,EZH2,CDT1,MND1,KIF15,MCM2,AURKB,BUB1B,ANLN,SHCBP1,WDR76,ZWINT,CDCA5,UBE2T,CDK1,NUF2,ESCO2,ATAD2,ZNF367,MCM4,CDC25A,HMGB3,GTSE1,PBK,MYBL2,CENPA,UHRF1,INCENP,MID1IP1,ASF1B,MELK,CDC25C,HJURP,SPC25,TCF19,CENPW,KIF20A,NEK2,PKMYT1,ARHGAP11A,PRC1,CDC7,NCAPH,DEPDC1B,RAD54L,CLSPN,KIF4A,CCNA2,KIF23,EXO1,PLK4,CORO7,MAD2L1,DSCC1,NUSAP1,MCM5,CDKN2D,SNRNP25,BLM,PCNA,KIF2C,KIF18B,KIFC1,FBXO5,MXD3,KIF18A,SCD,CDKN3,NET1,DTL,CDCA3,RAD51,TYMS,STMN1,LMNB1,GINS1,GGH,PLK1,MCM3,AQP1,RACGAP1,RCC1,CCNF,FANCI,DLGAP5,TUBA1B,GMNN,ESPL1,ERCC6L

5	wgEncodeHaibTfbsEcc1Foxm1sc502V0422111PkRep2.broadPeak.gz	0.18165	8.35538	T3	FOXM1	CCNB1,CDK1,GPSM2,CDKN3,UBE2C,CENPA,TROAP,GTSE1,CCNA2,TPX2,CCNF,LMNB1,MXD3,CCNB2,PRC1,PLK1,SKA2,KIF2C,NCAPH,RACGAP1,NUF2,KIF18B,PTTG1,KIF23,KIF20A,HJURP,KIF18A,HMMR,INCENP,RGS3,NUSAP1,CD36,UBE2T,CDKN2D,ESPL1,KIF4A,AURKA,UHRF1,NEK2,KIFC1,TTK,CDC20,AURKB,NEIL3,ANLN,FBXO5,TK1,MAD2L1,S100A10,ATAD2,DLGAP5,BIRC5,CDCA8,E2F2,ASF1B,NDC80,KIF15,EZH2,ID1,NCAPG,PBK,DIAPH3,CDC25C,LAMB3,TACC3,CENPE,G0S2,SPAG5,ESCO2,PCNA,HMGB3,TYMS
6	wgEncodeHaibTfbsEcc1Foxm1sc502V0422111PkRep1.broadPeak.gz	0.16689	7.5905	T3	FOXM1	GPSM2,CDK1,CCNB1,CDKN3,CCNA2,TROAP,UBE2C,CENPA,TPX2,SKA2,RACGAP1,LMNB1,PTTG1,HJURP,CCNB2,NUSAP1,NUF2,CCNF,GTSE1,PLK1,PRC1,KIF23,INCENP,NEK2,KIF2C,KIF20A,UBE2T,KIF18A,MXD3,AURKA,NCAPH,KIF18B,CDKN2D,CD36,KIF4A,HMMR,RGS3,CDC20,AURKB,NDC80,TTK,ESPL1,ANLN,E2F2,DIAPH3,KIFC1,MAD2L1,KIF15,SPAG5,NEIL3,S100A10,ID1,ATAD2,FBXO5,EZH2,UHRF1,DEPDC1B,LAMB3,SPC25,ASF1B,BUB1B,DLGAP5,BIRC5,CENPE,G0S2,PBK,CDCA8,PCNA,CDC25C,TACC3,CKS2,PLK4,TK1
7	wgEncodeHaibTfbsSknshFoxm1sc502V0422111PkRep2.broadPeak.gz	0.165501	7.51852	T3	FOXM1	GPSM2,CCNA2,CDKN3,CENPA,CCNB1,CDK1,GTSE1,CCNF,RASGRP3,LMNB1,HJURP,CDKN2D,PTTG1,UBE2C,NUF2,KIF20A,TPX2,PLK1,KIF23,TROAP,PRC1,NEK2,CCNB2,KIF18B,RACGAP1,INCENP,EZH2,SKA2,KIF2C,MXD3,UBE2T,KIF18A,NCAPH,PDCD1LG2,ESPL1,KIF15,AURKA,HMMR,PBK,KIF4A,BUB1B,TTK,KIFC1,NUSAP1,BIRC5,CDC20,ARHGAP11A,NEIL3,DLGAP5,MMP2,SIGLEC1,ZWINT,IFI35
8	wgEncodeHaibTfbsMcf7Foxm1sc502V0422111PkRep1.broadPeak.gz	0.157616	7.10985	T3	FOXM1	CDKN3,CDK1,GPSM2,CCNB1,UBE2C,CENPA,LMNB1,SKA2,KIF23,CCNF,TROAP,PTTG1,RACGAP1,NUF2,CCNA2,CDKN2D,PRC1,HJURP,KIF20A,NCAPH,ADORA3,BIRC5,CCNB2,GTSE1,TPX2,KIF18B,MXD3,RGS3,PLK1,KIF2C,KIF18A,UBE2T,FBXO5,INCENP,ATAD2,KIFC1,ANLN,NEK2,ESPL1,HMMR,EZH2,TACC3,AURKA,AURKB,TTK,PBK,SPAG5,NUSAP1,ID1,DEPDC1B,HMGB3,UHRF1,MYB,NEIL3,SMPDL3A,KIF4A,DLGAP5,CDC25C,NCAPG2,CD36,CDCA8,FANCI
9	wgEncodeHaibTfbsPanc1Sin3ak20V0416101PkRep2.broadPeak.gz	0.144428	6.4264	T4	SIN3A	ANLN,ID1,KIF2C,FANCI,DTL,NEK2,NUF2,BLM,FBXO5,TPX2,IQGAP3,RAD51,TTK,CCNB2,CDC20,CDCA5,CDK1,EXO1,AURKA,HELLS,SHCBP1,ORC1,MCM4,RAD54L,BUB1B,CCNB1,CENPQ,KIF23,HMMR,CCNA2,CCNE2,BIRC5,PRC1,UBE2C,CLSPN,ATAD2,KIF18B,KIF20A,PLK4,AURKB,KIF18A,CENPW,NCAPH,NUSAP1,KIF4A,DSCC1,TK1,KIFC1,GGH,NET1,PCNA,TROAP,MAD2L1,ZWINT,CDCA8,PLK1,HJURP,CDC25C,DLGAP5,NCAPG,TCF19,CDC25A,CDC45,TRIP13,HSPA1B,RCC1,RAD54B,SPAG5,MELK,CDCA3,ERCC6L,LMNB1,GINS1,CDKN2D,ASF1B,GTSE1,MYBL2,CENPA,NEIL3,SLC25A19

10	wgEncodeHaibTfbsSknshSin3ak20V0416101PkRep1.broadPeak.gz	0.141161	6.25714	T4	SIN3A	FAM72B,KIF2C,FAM72D,SKA2,NCAPG,CDC20,NUF2,KIF20A,SHCBP1,IQGAP3,MAD2L1,GGH,TK1,KIF18B,NEK2,NCAPG2,ESCO2,BIRC5,DLGAP5,BUB1B,CCNB1,HJURP,GTSE1,TROAP,CCNA2,NET1,CLSPN,MND1,CDCA8,RCC1,KIF15,FBXO5,CENPA,TACC3,CCNE2,RAD51,KIF4A,NEIL3,CDCA5,AURKB,EXO1,PLK1,NUSAP1,FANCI,MYBL2,PBK,ARHGAP11A,RAD54L,PCNA,BLM,CENPW,ANLN,UBE2C,CDCA3,KIF18A,TUBA1B,RAD54B,MCM5,DTL,CDC25A,CDK1,ESPL1,KIF23,LMNB1,RACGAP1,TCF19,GINS1,CKS2,MXD3,TTK,RASGRP3,NDC80,CENPQ,CDC45,SLC25A19,UHRF1,KIFC1,TYMS,ZNF367,TUBA1A,CDKN2D,SPC24,ZWINT,UBE2T,DIAPH3,MCM2,AURKA,NCAPH,CDC25C,PARP9,PLK4,ORC1,SPARC,HMMR,CENPE,ERCC6L,TUBB3,LIMA1,PRC1,DSCC1,STMN1,CCNB2,HELLS
11	wgEncodeSydhTfbsMcf10aesE2f4TamHvdPk.narrowPeak.gz	0.118446	5.07993	T1	E2F4	ZNF367,GINS2,TCF19,NCAPG2,CDT1,TACC3,ASF1B,MYBL2,TYMS,TROAP,HMGB3,FAM72D,BIRC5,ARHGAP11A,PLK1,FOXM1,TK1,CDCA5,CDCA8,CDKN3,CLSPN,CENPA,NUSAP1,CORO7,PRC1,UBE2C,MCM2,NCAPH,FADS2,IRF7,CDKN2D,ERCC6L,ANLN,CDC25A,MCM5,GTSE1,RAD51,E2F2,ESPL1,CDCA3,FAM72B,EZH2,SKA2,MCM4,FANCI,UHRF1,AURKB,NET1,KIF4A,PKMYT1,CCNF,RAD54L,CKS2,RCC1,CDC25C,GMNN,CDC20,AURKA,BLM,MELK,CDC7,DLGAP5,RACGAP1,CCNB1,SHCBP1,ATAD2,INCENP,NCAPG,KIF15,UBE2T,LMNB1,PCNA,MXD3,TUBA1B,SPC25,NEIL3,TRIP13,MND1,MCM3,PBK,FEN1,ZWINT,KIF2C,CENPW,KIFC1,DIAPH3,KIF23,KIF18B,WDR76,GPSM2,SPAG5,TPX2,PLK4,NEK2,FBXO5,DSCC1,MAD2L1,SREBF1,KIF20A,GINS1,IQGAP3,ID1,CCNB2,CDC45,DEPDC1B,ORC1,MID1IP1,CENPQ,GGH,NDC80
12	wgEncodeHaibTfbsHepg2Mybl2sc81192V0422111PkRep1.broadPeak.gz	0.109618	4.62246	T5	MYBL2	ID1,SKA2,AURKA,EZH2,CKS2,PLK1,HMMR,SERPINA1,TUBA1B,TPX2,CENPA,CDKN2D,PRC1,CCNB1,LMNB1,NUSAP1,FBXO5,KIFC1,HJURP,TYMS,RACGAP1,BIRC5,UBE2C,PCNA,NCAPG,BUB1B,ANLN,KIF20A,INCENP,KIF15,NDC80,TUBB3,FAM72D,KIF18B,CDC20,CCNA2,CDK1,GTSE1,TK1,PBK,CENPE,AURKB,CCL20,ASF1B,ARHGAP11A,UBE2T,NCAPH,NEK2,CDCA8,ATAD2,PSRC1,FAM72B,NUF2,DEPDC1B,PLK4,DLGAP5,MCM4,MXD3,KIF2C,IL1RN,GMNN,CDCA3,UHRF1,MAD2L1,KIF23,HSPA1B,SPC25,RGS3,DTL,CDKN3,ARHGAP18,CDC25C,CCNB2,LYZ,HSPA6,TTK
13	GSM1208730_batch2_chrom1_LoVo_E2F7_PassedQC_peaks_hg19	0.109471	4.61484	T6	E2F7	TYMS,MCM5,E2F2,WDR76,MCM3,ZNF367,RAD51,MCM4,PCNA,ATAD2,TK1,DTL,CDC25A,CDC45,MCM2,LMNB1,HELLS,RAD54L,GMNN,ASF1B,GINS2,MXD3,ORC1,EXO1,SKA2,FANCI,NUSAP1,CDC7,CCNE2,STMN1,BLM,PRC1,CDC25C,FBXO5,DSCC1

14	wgEncodeHaibTfbsMcf7Foxm1sc502V0422111PkRep2.broadPeak.gz	0.105781	4.42362	T3	FOXM1	CDKN3,CDK1,GPSM2,UBE2C,SKA2,CCNB1,CENPA,KIF23,CCNA2,ADORA3,TPX2,LMNB1,CD36,TROAP,CCNF,PRC1,S100A10,NUF2,KIF20A,NEK2,RACGAP1,PTTG1,HJURP,MXD3,NCAPH,KIF18B,FBXO5,CDKN2D,ID1,PLK1,SMPDL3A,IRF8,UBE2T,INCENP,CDC20,GTSE1
15	wgEncodeHaibTfbsSknshFoxm1sc502V0422111PkRep1.broadPeak.gz	0.0855078	3.37297	T3	FOXM1	RASGRP3,CDK1,LMNB1,GPSM2,CCNB1,CCNF,UBE2C,NEK2,GTSE1,TROAP,CDKN3,CENPA,CCNA2,PTTG1,CDKN2D,NCAPH,HJURP,CCNB2,MYLIP,TPX2,PDCD1LG2,SKA2
16	wgEncodeSydhTfbsMcf7Hae2f1UcdPk.narrowPeak.gz	0.0854902	3.37206	T7	E2F1	UHRF1,CDK1,DTL,RAD54L,MCM3,PBK,PLK4,WDR76,ZWINT,UBE2T,NCAPG,CDC7,PCNA,MCM4,PRC1,TP73,ASF1B,CLSPN,TCF19,CDC25A,CDC45,KIF18B,MCM2,KIF15,CCNE2,NEK2,CDKN2D,NUSAP1,GMNN,NDC80,FEN1,FANCI,GINS1,FBXO5,ESCO2,ORC1,TYMS,EZH2,SKA2,SREBF1,ATAD2,SPAG5,PARP9,EXO1,KIFC1,BLM,SCD,GGH,CDC25C,LMNB1,INCENP,KIF2C,CDT1,E2F2,RCC1,NET1,ADORA3,TUBA1B,MCM5,SPC25,CENPQ,KIF4A,GTSE1,CORO7,GPSM2,KIF18A,RAD54B,SPC24,SLC25A19,DSCC1,RACGAP1,DIAPH3,NCAPH,HJURP,FPR3,ZNF367,MND1,HELLS,NCAPG2,BUB1B,MYB,PKMYT1,AURKB,NUF2,RGS3,HSPA6,ANLN,IQGAP3,MYBL2,NEIL3,SHROOM2,CDCA8,CHRNA5,AURKA
17	wgEncodeHaibTfbsMcf7Sin3ak20V0422111PkRep2.broadPeak.gz	0.0814472	3.16254	T4	SIN3A	SKA2,ATAD2,CCNA2,CDC25C,AURKB,NEIL3,CCNE2,TUBA1B,TTK,ZNF367,FANCI,UBE2C,PLK4,FBXO5,PLK1,BLM,ESCO2,CDKN2D,RAD51,DEPDC1B,CDC20,NET1,MXD3,MCM4,MYB,TUBB3,KIF20A,CCNB1,CDK1,DIAPH3,KIF23,ADORA3,DSCC1,TYMS,NUF2,AURKA,PCNA,HSPA1B,BIRC5,ID1,CCNB2,TRIP13,CD36,ANLN,KIF4A,CDCA5,MYBL2,NEK2,RAD54B,KIF2C,BUB1B,EXO1,NCAPG2,NUSAP1,GINS1,ASF1B,TROAP,MAD2L1,LMNB1,RAD54L,SPARC,CKS2,KIF18A,KIF18B,PRC1,TPX2,IQGAP3,CDCA8,CENPW,GGH,PBK,PKMYT1,S100A10,CLSPN,HJURP,HSPA1A,DTL,RGS3,INCENP,STMN1,NCAPH,WDR76,UBE2T,CDC45,CDC25A,CENPA,MCM3,NCAPG,TDRD9,TUBA1A,GMNN,TACC3,TK1,DLGAP5,FEN1,SLC25A19,E2F2,CENPQ,SPC24,HMMR,SHCBP1,FXYD2,CENPE,NDC80,MYLIP,PTTG1,SREBF1,ZWINT,TCF19,PSRC1,IRF8,MCM2,RACGAP1,KIFC1,SCD,MND1,RCC1,MID1IP1,ORC1,ESPL1,CDC7,CDKN3
18	wgEncodeHaibTfbsHepg2Sin3ak20Pcr1xPkRep2.broadPeak.gz	0.0802232	3.0991	T4	SIN3A	HSPA1B,HSPA1A,FAM72D,ARHGAP11A,FAM72B,FBXO5,SHCBP1,CDC25A,NEK2,CCNA2,MAD2L1,CCNE2,NCAPG,PCNA,TRIP13,CCNB1,ANLN,NUF2,TACC3,CLSPN,RAD51,PLK1,KIF20A,SKA2,FANCI,CDC20,MYBL2,AURKA,HJURP,FOXM1,KIF18A,RAD54L,CCNB2,KIF15,CKS2,CDKN2D,ATAD2,NCAPG2,KIFC1,NEIL3,PLK4,TUBB3,BLM,PBK,BUB1B,NCAPH,ESCO2,KIF4A,SREBF1,MXD3,CDCA5,ASF1B,KIF18B,NUSAP1,MND1,ID1,IQGAP3,DLGAP5,GINS1,GMNN,CENPQ,AURKB,TROAP,BIRC5,TTK,CDCA8,PRC1,TCF19,DTL,NET1,DIAPH3,KIF2C,FEN1,CDK1,CDC25C,KIF23,TPX2

Supplementary Table 8. Transcription factor predicted by Enrichr

Transcription factors predicted by DEGs upregulated in HLF cells

Term	Overlap	P-value	Adjusted P-value	Old P-value	Old Adjusted P-value	Odds Ratio	Combined Score	Genes
AR CHEA	5/1095	0.0006	0.0206	0	0	9.63	70.77	ABCA10;RELN;ABLIM3;PSAT1;PAPPA2
SUZ12 CHEA	4/1684	0.0253	0.4042	0	0	4.36	16.03	RELN;SLIT2;GPR156;SLC7A2
FOS ENCODE	2/637	0.0716	0.5545	0	0	5.08	13.39	FKBP14;PSAT1
SUZ12 ENCODE	1/105	0.0711	0.5545	0	0	14.71	38.88	SLIT2
ZC3H11A ENCODE	1/129	0.0866	0.5545	0	0	11.93	29.19	ABLIM3
BCL3 ENCODE	1/273	0.1751	0.5881	0	0	5.58	9.71	SYCE2
PBX3 ENCODE	2/1269	0.2217	0.5881	0	0	2.46	3.71	SYCE2;FKBP14
SALL4 CHEA	1/355	0.2218	0.5881	0	0	4.27	6.42	ASNS
SMC3 ENCODE	2/1181	0.1987	0.5881	0	0	2.66	4.30	ABLIM3;GPR156
STAT3 CHEA	1/177	0.1171	0.5881	0	0	8.66	18.57	ASNS

Transcription factors predicted by DEGs downregulated in HLF cells

Term	Overlap	P-value	Adjusted P-value	Old P-value	Old Adjusted P-value	Odds Ratio	Combined Score	Genes
IRF1 ENCODE	8/263	0.0000	0.0000	0	0	15.45	238.89	ANKRD37;PLSCR1;STAT1;MX1;CMPK2;ISG15;IFI35;PARP9
IRF8 CHEA	5/121	0.0000	0.0004	0	0	19.88	228.19	IFIH1;ISG20;CD74;EPSTI1;IFI35
STAT3 ENCODE	7/725	0.0016	0.0430	0	0	4.57	29.41	HERC5;ISG20;PLSCR1;RRM2;SP110;STAT1;TMEM140
VDR CHEA	2/76	0.0144	0.2872	0	0	11.68	49.56	SP110;EPSTI1
NELFE ENCODE	3/234	0.0186	0.2973	0	0	5.69	22.68	RRM2;SNHG12;FASN
SALL4 CHEA	3/355	0.0534	0.7117	0	0	3.71	10.88	FASN;E2F1;PRSS23
E2F6 ENCODE	12/3245	0.0781	0.8140	0	0	1.72	4.40	SREBF1;ANKRD37;PLSCR1;RRM2;SNHG12;STAT1;FASN;E2F1;TRIM14;CMPK2;LAP3;PARP9
POU5F1 CHEA	2/261	0.1297	0.8140	0	0	3.31	6.75	PLSCR1;FASN
PPARG CHEA	3/535	0.1368	0.8140	0	0	2.43	4.84	LPCAT3;TMEM140;BCL2L1
RELA ENCODE	3/484	0.1099	0.8140	0	0	2.70	5.96	IFIH1;CD74;STAT1

Transcription factors predicted by DEGs upregulated in macrophages

Term	Overlap	P-value	Adjusted P-value	Old P-value	Old Adjusted P-value	Odds Ratio	Combined Score	Genes

CEBPB ENCODE	7/144	0.0033	0.1882	0	0	3.84	21.89	XPOT;TNNC1;PYCR1;CHAC1;SLC7A11;TBL1X;GTPBP2
ZBTB7A ENCODE	44/2184	0.0038	0.1882	0	0	1.61	8.98	NCKAP1;ZFAND3;PHF1;TESK1;PRSS27;SLC1A4;AKAP5;RFNG;SENP6;CBS;FTH1;CYP4V2;MKNK2;ZNF425;D2HGDH;TBL1X;DCAF10;PRKACB;PDE8A;SH3GLB1;HEMK1;UNC13A;BRPF3;PYCR1;ELOVL6;SLC30A1;GTPBP2;NBPF1;KIF27;P2RX4;XPOT;MYO15B;RILPL1;CCPG1;MAP1B;UVSSA;NOXA1;CCSER2;TCEA1;FLYWCH1;SQSTM1;KCTD15;ATF4;PPIL6
E2F6 ENCODE	50/3245	0.1577	1.0000	0	0	1.19	2.19	DHRS12;SETD9;PHF1;FBXO27;TESK1;ALDH1L2;CLGN;PTER;NAMPT;PDE8A;CCDC112;FBXW4;PARP6;SLC38A1;GPT2;VPS13C;WDR31;GAB2;GTPBP2;GPCPD1;NBPF1;KIF27;MTHFD2;RILPL1;ATF5;SQSTM1;FTCDNL1;ATF4;EPAS1;NPL;SLC1A5;CBS;STC2;MAP7;ZNF425;DCAF10;PCDH1;PCK2;DTNB;PYCR1;NR1D2;SNHG7;TDRD3;PER3;MYO15B;PSAT1;TCEA1;ULBP1;NFE2L1;PPIL6
ESR1 CHEA	5/154	0.0566	1.0000	0	0	2.50	7.18	CYP27A1;C1QTNF6;CAPN5;PDK4;GAB2
NFE2L2 CHEA	18/1022	0.1445	1.0000	0	0	1.34	2.60	SLC48A1;GRIA2;UNC5B;GPT2;SLC41A2;ARAP2;NR1D2;TUBE1;SLC30A1;SLC1A4;SLC7A11;SIPA1L2;GPCPD1;FAM210B;FTH1;STC2;GRB10;SQSTM1
RUNX1 CHEA	23/1294	0.1018	1.0000	0	0	1.36	3.12	CCDC112;DENND2D;SH3GLB1;SLC38A1;UNC13A;DTNB;ACSL1;TSEN15;STRBP;ICA1;ELOVL6;FGR;LGALS3;MTHFD2;CCPG1;RPS6KA2;FTH1;TNIP3;MKNK2;GRB10;TBL1X;NFE2L1;NSMAF
SALL4 CHEA	7/355	0.2005	1.0000	0	0	1.49	2.40	UNC5B;RUFY3;SESN2;ASNS;ELOVL6;PDE8A;NSMAF
TCF3 CHEA	17/1006	0.1943	1.0000	0	0	1.28	2.10	UNC5B;FBXO27;SLC41A2;ASNS;ELOVL6;SLC1A5;TDRD3;SEL1L3;KLF8;SPIRE1;PLIN2;TBL1X;RFFL;PRKACB;PDE8A;ZBED3;NSMAF
TCF3 ENCODE	15/840	0.1591	1.0000	0	0	1.36	2.50	SH3GLB1;FBXW4;SLC48A1;ACSL1;NR1D2;TDRD3;MAN2A2;NAMPT;CHAC1;TBL1X;PDE8A;SQSTM1;NFE2L1;ATF4;RNF187
TP53 CHEA	7/319	0.1382	1.0000	0	0	1.67	3.30	FBXW4;SMARCD3;UNC5B;VPS13C;SESN2;SLC30A1;SPATA18

Transcription factors predicted by DEGs downregulated in macrophages

Term	Overlap	P-value	Adjusted P-value	Old P-value	Old Adjusted P-value	Odds Ratio	Combined Score	Genes
E2F4 ENCODE	103/710	0.0000	0.0000	0	0	17.33	2972.50	ERCC6L;DSCC1;CCNF;GMNN;HJURP;BUB1B;CDC20;NUSAP1;NEK2;FBXO5;GTSE1;ESCO2;CDC25C;WDR76;CDC25A;NEIL3;DEPDC1B;MELK;CCNE2;KIF20A;ASF1B;BLM;CDCA3;CDCA5;TROAP;CDCA8;NCAPG;PKMYT1;IQGAP3;NCAPH;SKA2;RACGAP1;ORC1;CLSPN;PLK4;FANCI;CDT1;PLK1;CDC7;NDC80;NET1;TPX2;KIF18A;DIAPH3;KIF18B;UBE2T;KIF4A;CDK1;MXD3;EZH2;ARHGAP11A;FEN1;NCAPG2;FOXM1;KIF15;LMNB1;TUBA1B;EXO1;NUF2;PBK;MYBL2;TK1;ZNF367;DLGAP5;HELLS;RMI2;KIF23;CCNA2;ESPL1;INCENP;SLC25A19;MCM3;MCM4;BIRC5;KIF2C;MCM5;DTL;MCM2;PCNA;UHRF1;TTK;TYMS;AURKB;RAD54B;AURKA;CDC45;RAD54L;E2F2;GINS1;GINS2;CENPW;SPAG5;ATAD2;MND1;SHCBP1;RAD51;PRC1;TRIP13;CENPQ;SPC24;CDKN3;MAD2L1;SPC25

FOXM1 ENCODE	30/95	0.0000	0.0000	0	0	34.87	2530.14	GPSM2;TROAP;CCNF;HJURP;CENPA;NCAPH;LMNB1;SKA2;CDC20;CCNB2;CCNB1;PTTG1;RACGAP1;NUF2;NEK2;GTSE1;CDKN2D;UBE2C;PLK1;KIF23;CCNA2;TPX2;KIF18B;INCENP;PRC1;UBE2T;CDK1;KIF20A;MXD3;CDKN3
SIN3A ENCODE	60/1131	0.0000	0.0000	0	0	4.54	187.95	DSCC1;HJURP;BUB1B;KIF15;LMNB1;CDC20;TUBA1B;EXO1;NUF2;PBK;NEK2;FBXO5;GTSE1;ZNF367;DLGAP5;HELLS;KIF23;ESCO2;CDC25C;CDC25A;CCNA2;CCNE2;CKS2;MCM3;MCM4;BIRC5;KIF2C;KIF20A;DTL;BLM;CDCA5;CDCA8;NCAPG;HMMR;IQGAP3;NCAPH;AURKB;SKA2;AURKA;CCNB2;CCNB1;ORC1;CLSPN;PLK4;CENPW;PLK1;ATAD2;SHCBP1;TPX2;ANLN;CENPE;KIF18A;KIF18B;RAD51;ID1;TACC3;CDK1;TRIP13;CENPQ;MAD2L1
NFYA ENCODE	72/2250	0.0000	0.0000	0	0	2.66	62.84	CCNF;GMNN;HJURP;HMGB3;CDC20;NUSAP1;NEK2;FBXO5;TK1;GTSE1;ZNF367;DLGAP5;RMI2;KIF23;ESCO2;CDC25C;CDC25A;CCNA2;DEPDC1B;CCNE2;ESPL1;INCENP;SLC25A19;CKS2;MCM3;MCM4;MCM5;KIF20A;SNRNP25;DTL;FAM72B;ASF1B;BLM;PCNA;CDCA3;UHRF1;TROAP;CDCA8;TTK;HMMR;CENPA;AURKB;SKA2;AURKA;CCNB2;CCNB1;CDC45;RAD54L;CLSPN;PLK4;CDKN2D;GINS1;ATAD2;SHCBP1;ZWINT;NET1;TPX2;KIF18A;DIAPH3;KIF18B;SCD;PRC1;UBE2T;ID1;CDK1;CORO7;CENPQ;SPC24;MXD3;EZH2;CDKN3;SPC25
NFYB ENCODE	99/3715	0.0000	0.0000	0	0	2.31	51.96	ERCC6L;CCNF;GMNN;HJURP;CDC20;TUBB3;STMN1;NUSAP1;NEK2;FBXO5;GTSE1;MYLIP;ESCO2;CDC25C;CDC25A;DEPDC1B;CCNE2;KIF20A;SNRNP25;ASF1B;BLM;CDCA3;TROAP;CDCA8;ARHGAP18;HMMR;SKA2;CCNB2;CCNB1;RACGAP1;CLSPN;PLK4;UBE2C;PLK1;CDC7;ZWINT;BST2;NET1;TPX2;KIF18A;DIAPH3;KIF18B;UBE2T;KIF4A;ID1;CDK1;CORO7;MXD3;EZH2;GPSM2;NCAPG2;HMGB3;LMNB1;TK1;ZNF367;DLGAP5;RMI2;SHROOM2;KIF23;CCNA2;DEPDC7;PSRC1;ESPL1;INCENP;SLC25A19;CKS2;MCM3;MCM4;MCM5;FAM72D;DTL;FAM72B;MCM2;PCNA;UHRF1;IFI6;TTK;CENPA;AURKB;RAD54B;AURKA;CDC45;RAD54L;CDKN2D;GINS1;GINS2;SPAG5;MID1IP1;ATAD2;SHCBP1;CENPE;SCD;PRC1;APOC1;TACC3;CENPQ;SPC24;CDKN3;SPC25
E2F1 CHEA	34/859	0.0000	0.0000	0	0	3.04	48.55	ARHGAP11A;PCNA;CDCA5;GMNN;BUB1B;PKMYT1;AURKB;SKA2;LMNB1;AURKA;CCNB2;TUBA1B;EXO1;TK1;CLSPN;ZNF367;DLGAP5;S100A10;FANCI;CDT1;GINS2;RMI2;UBE2C;ATAD2;MND1;SHCBP1;CDC25A;NDC80;KIF18A;ESPL1;SLC25A19;SPC24;EZH2;MCM2
E2F6 ENCODE	66/3245	0.0022	0.0316	0	0	1.53	9.36	FEN1;ERCC6L;DSCC1;CCNF;HILPDA;GMNN;KIF15;LMNB1;TUBA1B;STMN1;MYB;NUF2;PBK;MYBL2;FBXO5;TK1;ZNF367;SREBF1;RMI2;KIF23;ESCO2;PARP9;WDR76;CDC25A;DEPDC1B;DEPDC7;CCNE2;SLC25A19;IRF7;MCM3;MCM4;BIRC5;SNRNP25;DTL;ASF1B;MCM2;BLM;CDCA3;CHRNA5;UHRF1;NCAPG;TYMS;CENPA;NCAPH;AURKB;SKA2;RAD54B;CDC45;RAD54L;E2F2;CLSPN;CDKN2D;GINS1;FANCI;CDT1;GINS2;ATAD2;CDC7;MND1;ANLN;RAD51;FABP5;CDK1;TRIP13;MXD3;EZH2

KLF4 CHEA	26/987	0.0025	0.0316	0	0	1.92	11.51	IFITM1;PCNA;UHRF1;DSCC1;SKA2;TUBA1B;TUBB3;STMN1;MYB;NUF2;SPP1;NEK2;MYBL2;ZNF367;TCF19;ANLN;DEPDC7;FABP5;APOC1;BIRC5;ACKR3;TRIP13;NBL1;CENPQ;DTL;IRF9
IRF3 ENCODE	19/663	0.0039	0.0435	0	0	2.08	11.53	BLM;CDCA3;UBE2C;CENPA;SKA2;AURKA;TPX2;INCENP;SLC25A19;MCM3;RAD54L;MCM5;FBXO5;FAM72D;DLGAP5;SPC24;ASF1B;CDKN3;SPC25
SPI1 CHEA	26/1056	0.0060	0.0611	0	0	1.79	9.12	TRAF3IP3;CDCA5;CELF2;PIK3CD;RAD54B;RGS1;ADORA3;TAGAP;CCL4;STK38L;CD14;FANCI;SP110;IPCEF1;KIF18B;RAD51;INCENP;KIFC1;SLC25A19;CKS2;TACC3;BIRC5;S100A4;DTL;MAD2L1;TRIM34

Red indicates adjusted P-value < 0.05
