# Supplementary material for: High‐Throughput Heterospheroid‐Based Screening Identifies Drugs That Reprogram Tumor‐Associated Macrophages
Source: J Immunol Res. 2026 Apr 20;2026:2889509. doi: 10.1155/jimr/2889509 (PMC13581048; doi:10.1155/jimr/2889509)
Supplement: Supplementary file 2 [file JIMR-2026-2889509-s002.pdf]

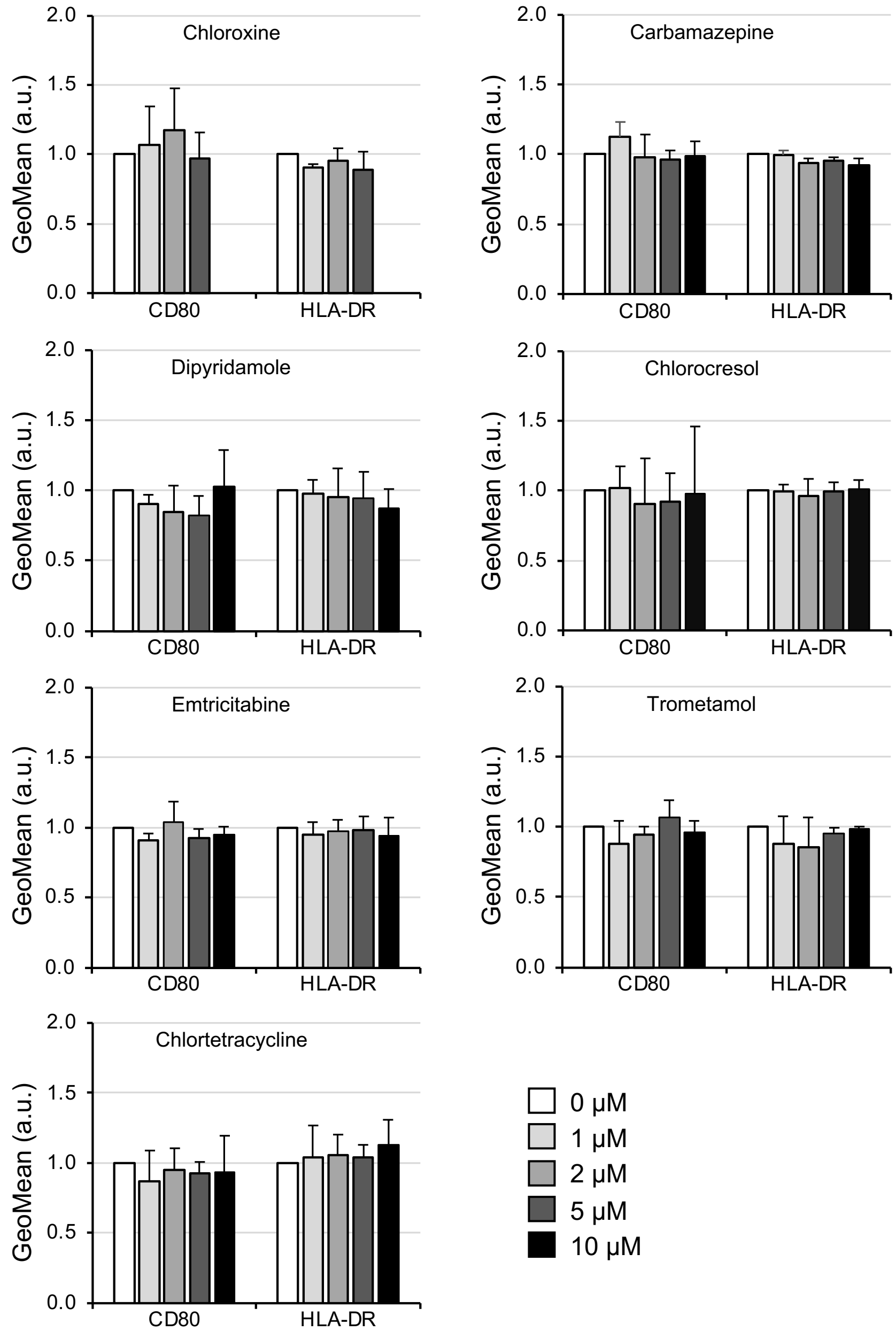

s

Supplementary Figure 1. Screening of TAM activators from an FDA-approved drug library. CD80 and HLA-DR expressions on THP-1-derived macrophages in heterospheroids with HLF cells. The heterospheroids were incubated with the indicated concentrations of each drug for 3 days. No significant difference was observed vs. 0 μM, Dunnett's test (*n* = 3).

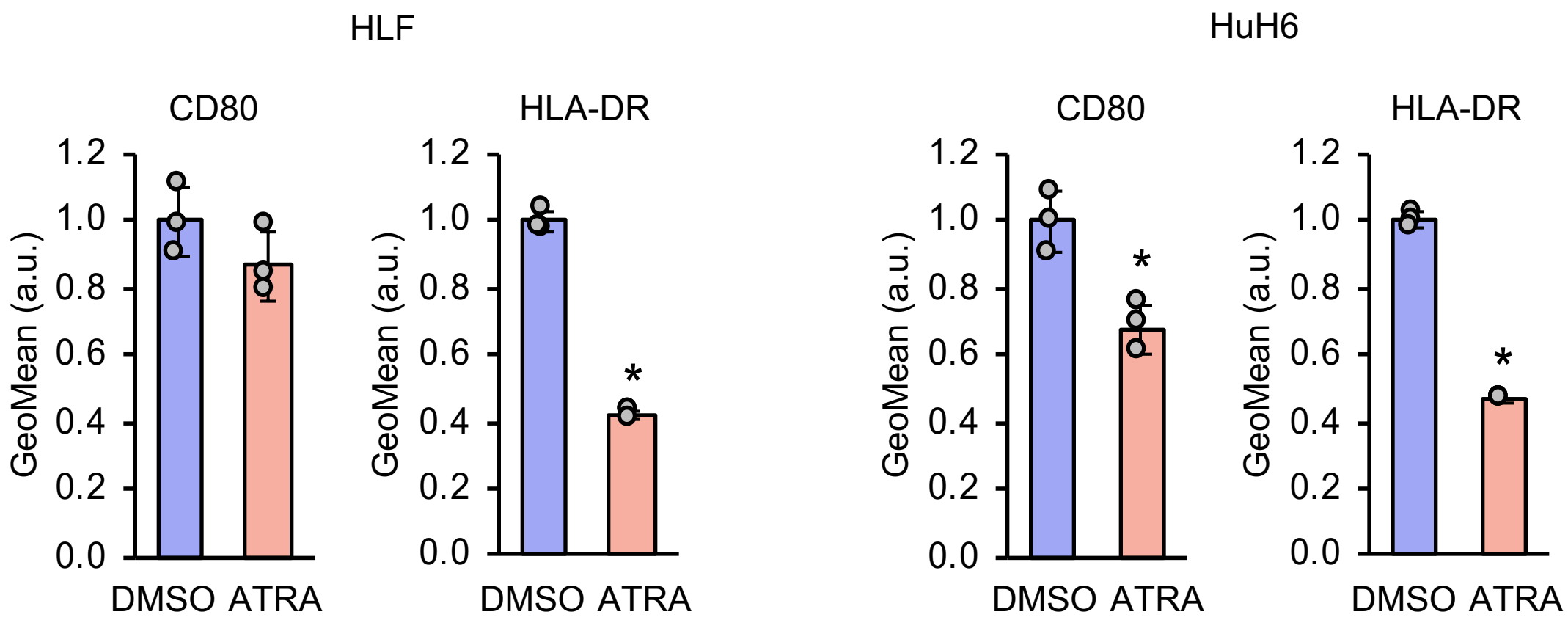

Supplementary Figure 2. Downregulation of M1 markers by all-*trans*-retinoic acid (ATRA).

CD80 and HLA-DR expressions on THP-1-derived macrophages in heterospheroids with HLF or HuH6 cells. The heterospheroids were incubated with 10 μM of ATRA for 3 days. *, $P < 0.05$ vs. DMSO, Student's *t*-test ($n = 3$).

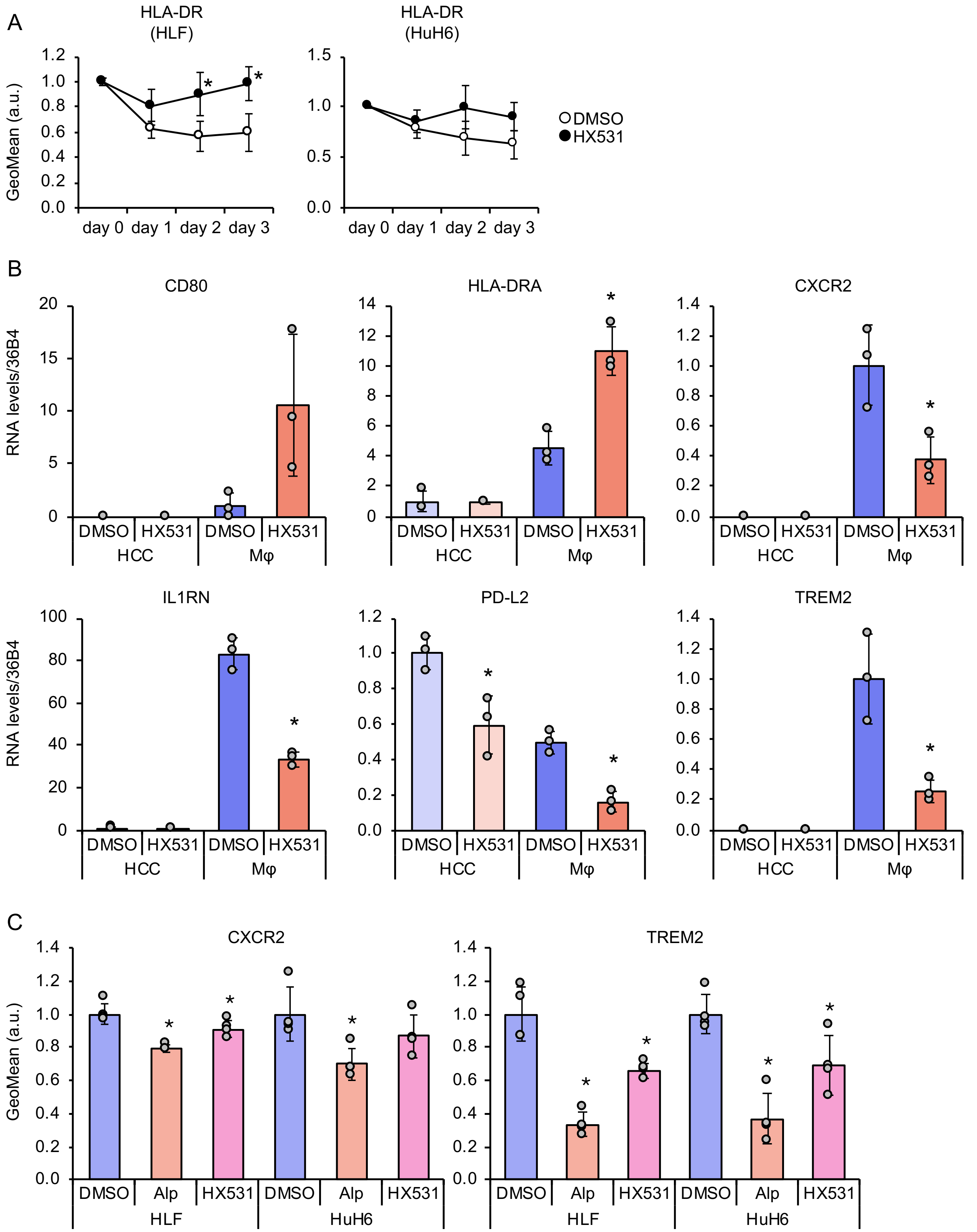

Supplementary Figure 3. RT-qPCR analysis of heterospheroids treated with HX531. (A) HLA-DR expressions on THP-1-derived macrophages in heterospheroids with HLF or HuH6 cells. The

heterospheroids were incubated with DMSO or 2 μM of ATRA for the indicated days. *, $P < 0.05$ vs. DMSO, Student's *t*-test ($n = 3$). (B) Validation of RNA-seq analysis. MRNA were recovered from THP-1-derived macrophages and HLF cells in heterospheroids treated with DMSO or 1 μM HX531 for 2 days, and subjected to RT-qPCR. *, $P < 0.05$ vs. DMSO, Student's *t*-test ($n = 3$). (C) Cell surface CXCR2 and TREM2 expressions on THP-1-derived macrophages in heterospheroids with HLF or HuH6 cells. The heterospheroids were incubated with DMSO, 2 μM alprostadil (Alp), or 1 μM HX531 for 3 days. *, $P < 0.05$ vs. DMSO, Dunnett's test ($n = 4$).

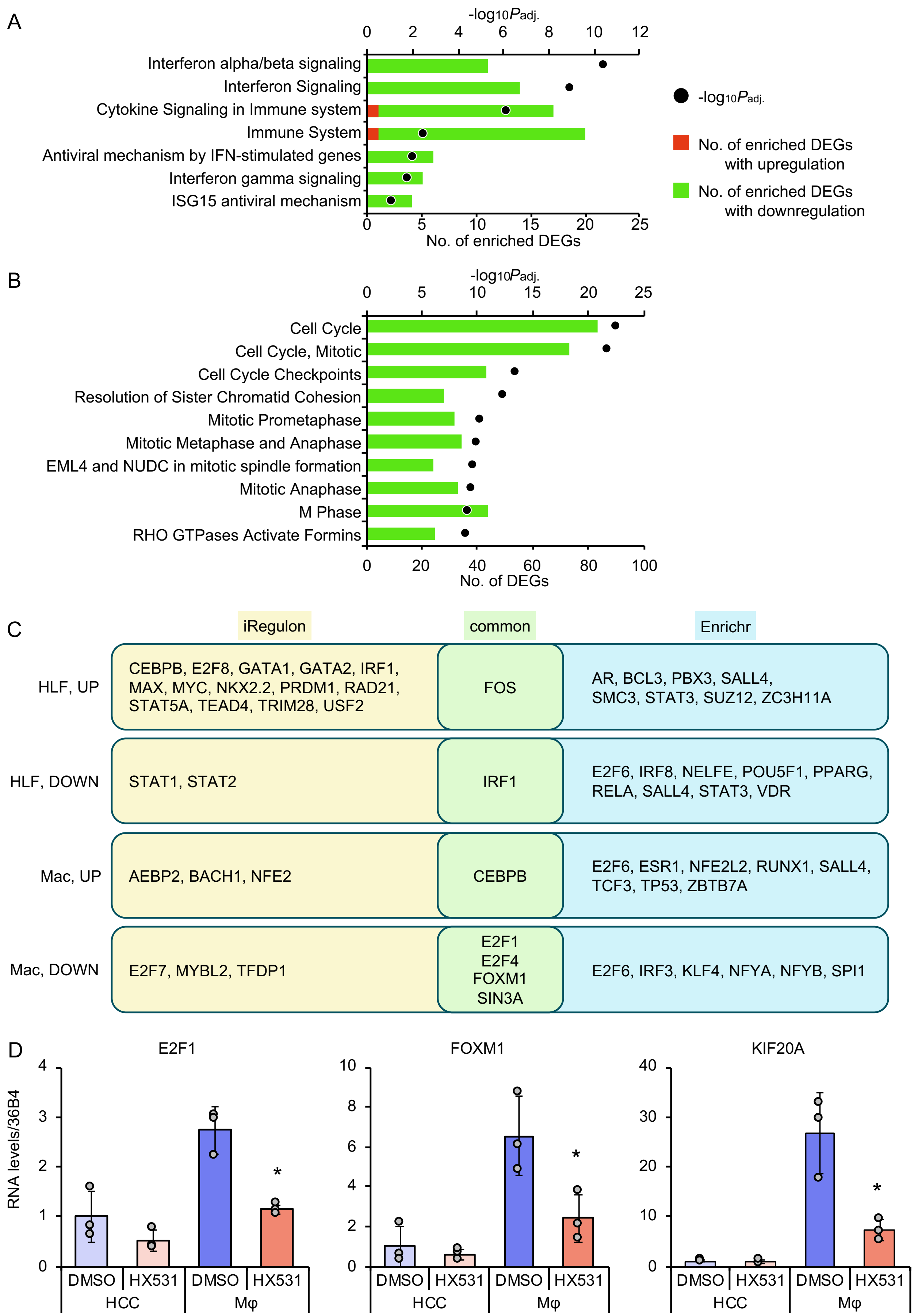
A
-log10Padj.
0 2 4 6 8 10 12
Interferon alpha/beta signaling
Interferon Signaling
Cytokine Signaling in Immune system
Immune System
Antiviral mechanism by IFN-stimulated genes
Interferon gamma signaling
ISG15 antiviral mechanism
0 5 10 15 20 25
No. of enriched DEGs
-log10Padj.
No. of enriched DEGs with upregulation
No. of enriched DEGs with downregulation
B
-log10Padj.
0 5 10 15 20 25
Cell Cycle
Cell Cycle, Mitotic
Cell Cycle Checkpoints
Resolution of Sister Chromatid Cohesion
Mitotic Prometaphase
Mitotic Metaphase and Anaphase
EML4 and NUDC in mitotic spindle formation
Mitotic Anaphase
M Phase
RHO GTPases Activate Formins
0 20 40 60 80 100
No. of DEGs
C
iRegulon
common
Enrichr
HLF, UP
CEBPB, E2F8, GATA1, GATA2, IRF1, MAX, MYC, NKX2.2, PRDM1, RAD21, STAT5A, TEAD4, TRIM28, USF2
FOS
AR, BCL3, PBX3, SALL4, SMC3, STAT3, SUZ12, ZC3H11A
HLF, DOWN
STAT1, STAT2
IRF1
E2F6, IRF8, NELFE, POU5F1, PPARG, RELA, SALL4, STAT3, VDR
Mac, UP
AEBP2, BACH1, NFE2
CEBPB
E2F6, ESR1, NFE2L2, RUNX1, SALL4, TCF3, TP53, ZBTB7A
Mac, DOWN
E2F7, MYBL2, TFDP1
E2F1
E2F4
FOXM1
SIN3A
E2F6, IRF3, KLF4, NFYA, NFYB, SPI1
D
E2F1
FOXM1
KIF20A
RNA levels/36B4
0 1 2 3 4
0 2 4 6 8 10
0 10 20 30 40
*
DMSO HX531 DMSO HX531
HCC
Mφ

Supplementary Figure 4. Pathway analysis of heterospheroids treated with HX531. (A,B) Reactome analyses of DEGs in HLF cells (A) and macrophages (B). (C) iRegulon and Enrichr analyses of DEGs in HLF cells and macrophages. (D) Validation of iRegulon and Enrichr analyses. MRNA were recovered from THP-1-derived macrophages and HLF cells in heterospheroids treated with DMSO or 1 μM HX531 for 2 days, and subjected to RT-qPCR. *, $P < 0.05$ vs. DMSO, Student's *t*-test ($n = 3$).

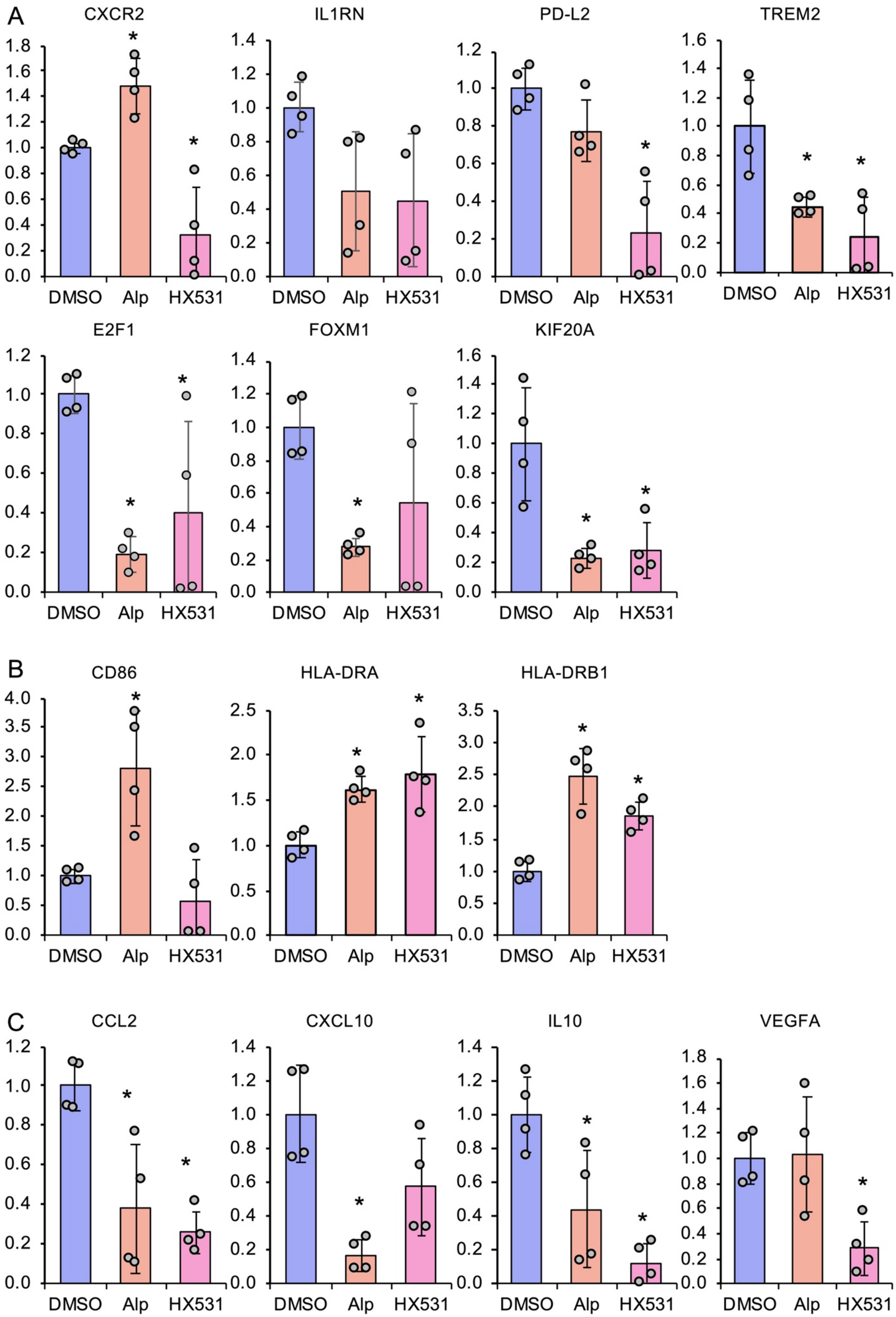
A
CXCR2
IL1RN
PD-L2
TREM2
E2F1
FOXM1
KIF20A
B
CD86
HLA-DRA
HLA-DRB1
C
CCL2
CXCL10
IL10
VEGFA
DMSO
Alp
HX531

Supplementary Figure 5. Direct effects of alprostadil and HX531 on macrophage polarity signatures. The mRNA expression of marker genes for M2 (A, C) and M1 (B) macrophage phenotypes. MRNAs were recovered from THP-1-derived macrophages in a planar monoculture treated with DMSO, 2 μM PGE1, or 1 μM HX531 for 2 days, and subjected to RT-qPCR. *, $P < 0.05$ vs. DMSO, Dunnett's test ($n = 4$).

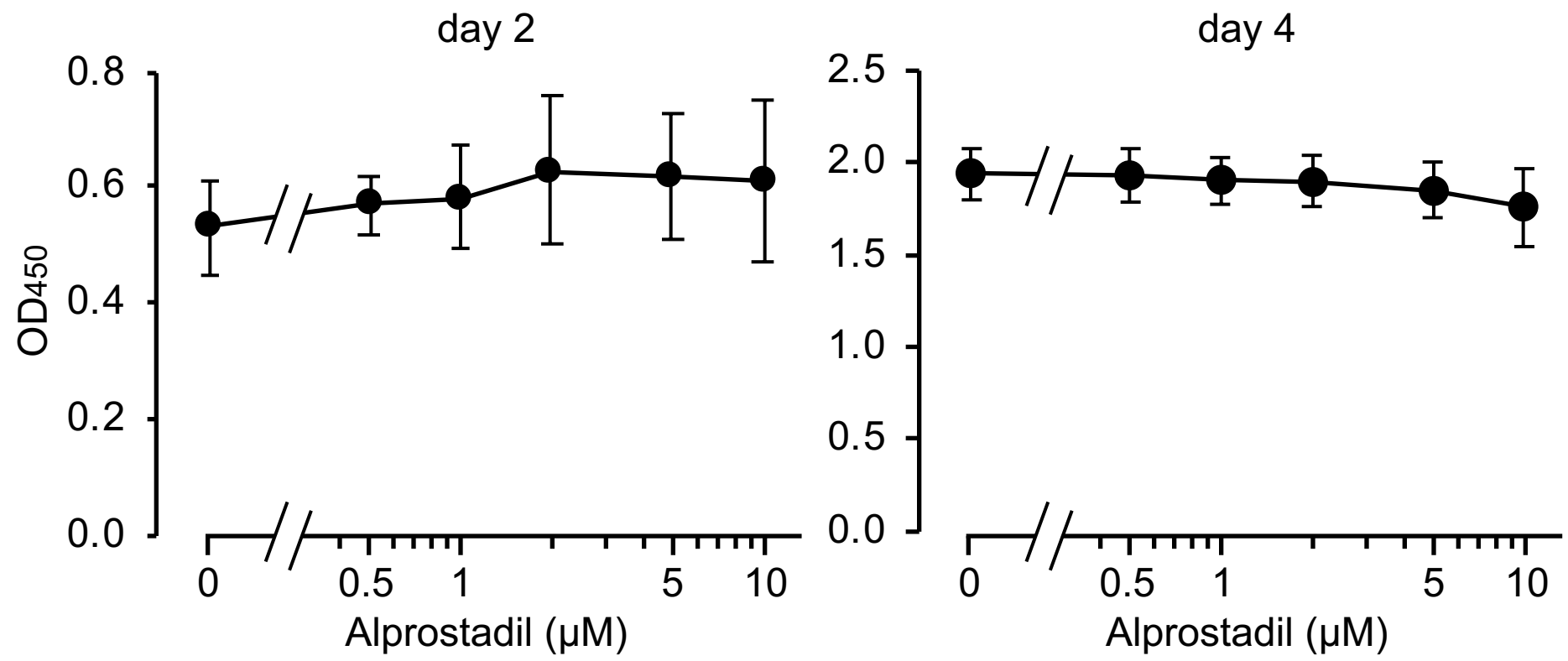

Supplementary Figure 6. The direct effects of alprostadil on the viability of Hepa 1-6 cells.

Hepa 1-6 cells were treated with the indicated concentrations ranging from 0 to 10 µM of alprostadil for 2 and 4 days. There were no significant differences in cell viability at any concentrations tested, as indicated by $P > 0.05$ vs. 0 µM, Dunnett's test ($n = 4$).

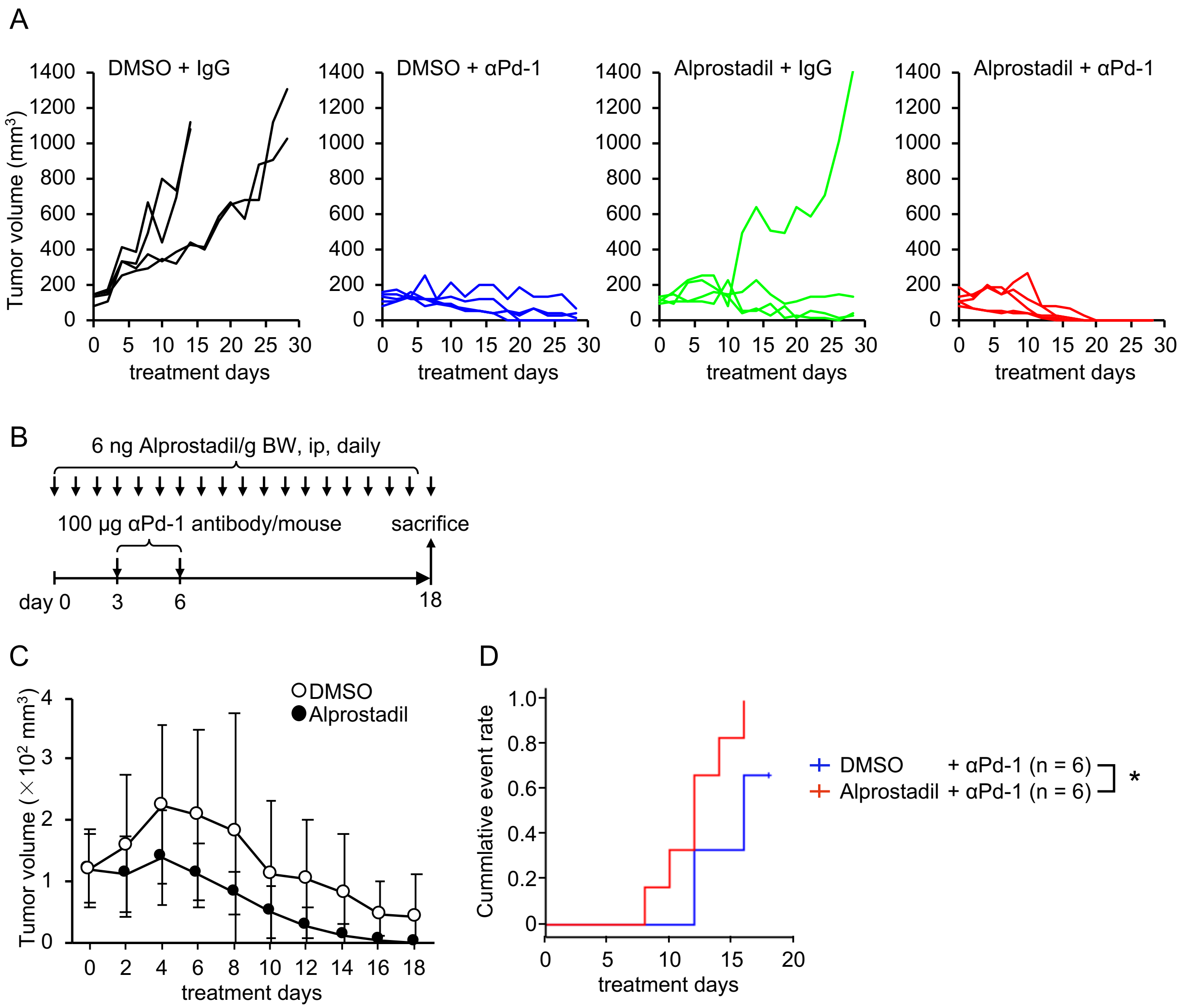

Supplementary Figure 7. *In vivo* efficacy of alprostadil. (A) Tumor volume changes in each mouse treated with the combination of alprostadil and anti-Pd-1 antibody, as shown in Figure 7G. (B) Treatment schedule of combination of alprostadil and a reduced total dose of anti-Pd-1 antibody. (C, D) Tumor volume changes (C) and cumulative tumor free rate (D) of mice treated with the combination of alprostadil and anti-Pd-1 antibody, as shown in Figure S6B. *, $P < 0.05$, log-rank test ($n = 6$).

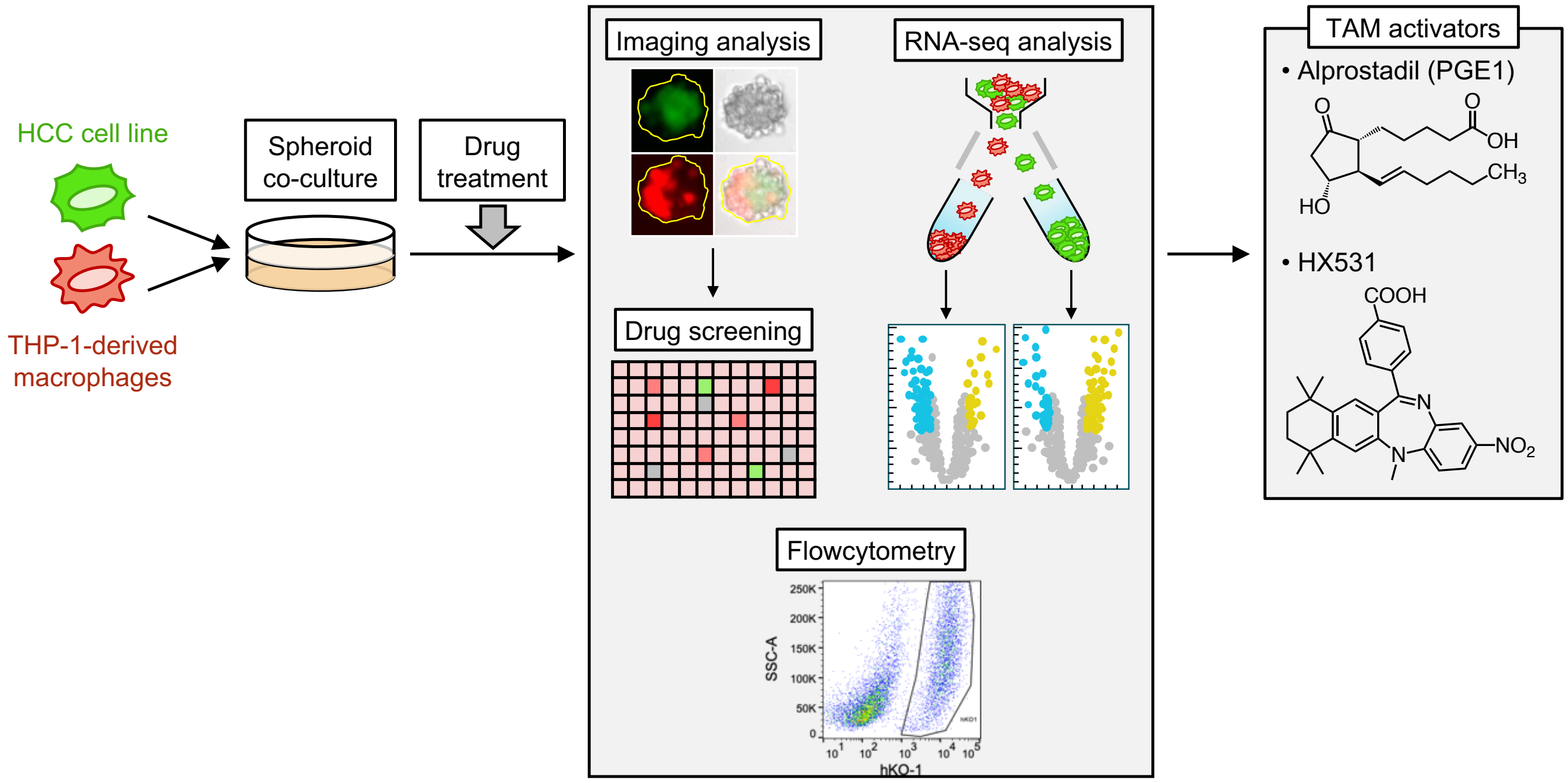

Supplementary Figure 8. Heterospheroids offer a new platform for oncoimmunological research and development.
